# How does progressivity impact tax morale? Experimental evidence across developing countries^☆^

**DOI:** 10.1016/j.jdeveco.2024.103398

**Published:** 2025-01

**Authors:** Christopher Hoy

**Affiliations:** World Bank, United States of America

**Keywords:** Political economy, Public finance, Redistribution, Tax compliance, Randomized experiment

## Abstract

This paper examines how the progressivity of taxes and government transfers impacts tax morale through a randomized survey experiment with over 30,000 respondents across eight developing countries. Respondents increased (decreased) their tax morale when they received accurate information that taxes in their country are progressive (not progressive). These effects were predominantly driven by respondents in cases where the information they received was counter to their prior beliefs and/or consistent with their preferences. These results suggest changes in policies that increase (decrease) the progressivity of tax systems may also lead to increases (decreases) in tax compliance.

## Highlights

• Tax morale increases when people receive information that taxes are progressive.
• Tax morale decreases when people receive information that taxes are not progressive.
• Most people prefer taxes to be progressive, even those who believe they are rich.
• Increasing tax progressivity may lead to increases in tax compliance.

## 1 Introduction

People’s willingness to pay tax has traditionally been conceptualized as a trade-off between the possibility of punishment for non-compliance compared to the cost of complying (Allingham and Sandmo, 1972); however, in recent years, there has been growing recognition of other factors that may influence people’s “tax morale” (Luttmer and Singhal, 2014, Slemrod, 2019, Antinyan and Asatryan, 2019).^1^ The extent to which governments redistribute income from rich to poor households through the tax and transfer system has been proposed as one of these factors (Prichard et al., 2019, Prichard et al., 2022). This is because most people prefer to live in societies with lower levels of inequality than what they perceive to exist (World Values Survey (WVS), 2022, Alesina and Giuliano, 2011), and many are supportive of greater government-led income redistribution (Pew Research Center, 2019, Alesina and Angeletos, 2005, Hoy and Mager, 2021a). As such, people may have higher tax morale when they believe the tax and transfer system is progressive (i.e., richer households pay a relatively higher share of their income in tax and poorer households receive a relatively higher share of their income in government transfers),^2^ and lower tax morale when they believe it is not progressive. However, to date, there has been an absence of empirical evidence about whether it is actually the case that progressivity influences people’s tax morale across developing countries.

I fill this important gap in knowledge by conducting a randomized survey experiment with over 30,000 respondents that is broadly representative of the population with internet access in eight countries (Colombia, Ghana, Indonesia, Jordan, Mexico, Sri Lanka, South Africa and Tanzania). This diverse set of countries makes up around 10 percent of the developing world’s population, is spread across Latin America, Africa, Asia and the Middle East, and had GNI per capita ranging from USD1,080 to USD8,480 (Atlas Method) in 2020 (World Bank, 2021). I exploit the fact that the progressivity of tax and transfer systems varies considerably across these countries (Commitment to Equity (CEQ) Institute, 2021). Respondents in each country were randomly allocated to receive accurate information about the progressivity of taxes (“taxes treatment”), government transfers (“transfers treatment”) or both (“combined treatment”) in their country, or to a control group that received no information. This information was sourced from a recently released database (hereafter the “CEQ database”) that uses a standardized approach across countries to monitor progress towards Sustainable Development Goal target 10.4 about increasing the redistributive impact of fiscal policy (Lustig et al., 2020). The progressivity of taxes and government transfers is measured using the Commitment to Equity (CEQ) Institute methodology^3^ and is based on recent Household Income and Expenditure Surveys that provide detailed information about *actual* income and consumption patterns of a nationally representative sample of households (Commitment to Equity (CEQ) Institute, 2021). As such the information treatments provided information about the de facto/effective (as opposed to de jure/statutory) rate of progressivity of taxes and transfers. People’s tax morale is measured using standardized questions from cross-country survey instruments (e.g., the Afrobarometer).^4^ I illustrate the channels through which information impacts people’s tax morale by examining heterogeneous treatment effects based on people’s prior beliefs and existing preferences. I compare these empirical findings to a modified version of (Allingham and Sandmo, 1972) seminal theory of what drives tax compliance that integrates (Alesina and Giuliano, 2011) workhorse model about how beliefs and preferences about inequality influence people’s utility.

The overall findings of the randomized survey experiment illustrate that people’s tax morale is influenced by whether there is progressivity in the tax and transfer system. Respondents who received the taxes treatment in the four countries for which taxes were progressive (Colombia, Ghana, Mexico and Tanzania) reported higher tax morale. In contrast, respondents who received the taxes treatment in the four countries for which taxes were not progressive (Indonesia, Jordan, Sri Lanka and South Africa) reported lower tax morale. The negative treatment effect on tax morale in Indonesia, Jordan, Sri Lanka, and South Africa was larger than the positive treatment effect on tax morale in Colombia, Ghana, Mexico, and Tanzania.^5^ These results are robust to a series of checks (such as comparing the results across treatments and removing respondents who took too little time or too long to complete the survey). The order of magnitude of the impact of the taxes treatment was in line with seminal cross-country randomized survey experiments (Alesina et al., 2018, Alesina et al., 2022). However, a clear limitation of this kind of study is that actual tax compliance behavior is not measured, although survey measures of tax morale are a plausible, but far from perfect, proxy for compliance (e.g., see discussion in Besley et al., 2021 and Luttmer and Singhal, 2014).

The overall treatment effects were predominantly driven by respondents in cases where the information they received was counter to their prior beliefs and/or in line with their preferences. These results are consistent with the conceptual framework that shows how prior beliefs and existing preferences about progressivity in the tax and transfer system are likely to impact people’s tax morale. Respondents who stated before the treatment that they prefer progressivity in the tax system and received accurate information that this was actually the case (i.e., those in Colombia, Ghana, Mexico and Tanzania) reported higher tax morale. Respondents who thought the tax system was progressive but received accurate information that it was not progressive (i.e., those in Indonesia, Jordan, Sri Lanka and South Africa) reported lower tax morale. There were similar, albeit weaker, results from the combined treatment (i.e., respondents who received information that the system was progressive (not progressive) reported higher (lower) tax morale) and no statistically significant effects on people’s tax morale from the transfers treatment. There were no notable trends in terms of heterogeneous treatment effects across other dimensions that were included in the pre-analysis plan (e.g., by respondents’ perceived place in the national income distribution). As such, these results suggest that people’s beliefs about and preferences for the progressivity of taxes matter more – in terms of driving tax morale responses to information about progressivity – than more traditional factors related to individual incentives, such as their position in the income distribution.

The findings from this study shed light on how changes in tax policy and/or administration that influence the de facto progressivity of taxes and transfers may influence people’s tax morale. Specifically, the results suggest that efforts to improve a country’s fiscal position by increasing (decreasing) equity in the tax and transfer system may also *have an additional benefit* (*potentially backfire*) by increasing (decreasing) people’s tax morale. This can be illustrated through the following stylized examples. Consider a tax policy reform that required richer households to pay more tax and which, by doing so, would make the tax and transfer system more progressive (e.g., an increase in the top marginal income tax rate). A consequence of this reform is that many taxpayers may be more likely to comply, especially if they prefer greater progressivity. Therefore, the improvement in total tax revenue collected could be greater than just the additional revenue that was intended to be gathered from richer households. Another illustrative example is a tax administration change that reduces the progressivity of the tax system, such as by disproportionately increasing enforcement efforts on smaller taxpayers (e.g., increasing revenue targets for the amount that inspectors are meant to collect from smaller firms). This approach could undermine many people’s tax morale and consequently not improve the fiscal position of the country as much as what was intended. In the extreme case, it could be possible that any expected increase in revenue would be entirely offset by falls in compliance. These stylized examples show how the findings from this study are relevant for policymakers in developing countries, especially as governments are making many changes to their tax and transfer systems as they face growing debt levels (World Bank, 2022a). In addition, the results show that even in the absence of changes in progressivity, communicating to taxpayers about the progressive aspects of the tax system in their country would appear to be a way to boost compliance.

This study makes several contributions to two broad strands of the existing literature. The first strand this study contributes to is concerning how people’s perceptions shape their preferences regarding tax and transfer policies (Gimpelson and Treisman, 2018, Hauser and Norton, 2017). Seminal work on this topic has been conducted in recent years using large-scale, randomized survey experiments in the United States and Western Europe examining a range of topics, such as inequality (Kuziemko et al., 2015, Bublitz, 2022), social mobility (Alesina et al., 2018) and immigration (Alesina et al., 2022). A common thread in these studies is that, in general, most people have a poor understanding of the economic circumstances in their country (e.g., about the level of inequality, see Norton and Ariely, 2011) and they have tested what happens to people’s general policy preferences when they are provided with accurate information. This study extends this literature in three ways. Firstly, this study is one of the first to test how accurate information about existing policies (specifically the progressivity of taxes and government transfers), as opposed to existing circumstances (e.g., the level of inequality), shifts people’s preferences. In other words, I directly alter people’s beliefs about the role the government currently plays in distributing resources in their country and see how people respond (in terms of their tax morale), as opposed to examining how people’s views change about what the role of the government should be once they are aware of the actual circumstances in their country. Secondly, this randomized survey experiment is one of the first to focus on measuring a specific policy area (people’s tax morale), which is an important way people relate to their government, as opposed to general preferences (e.g., support for redistribution). This allows for direct policy implications to emerge from this work. Thirdly, I conduct one of the first and largest randomized survey experiments in this literature in developing countries (the previously largest study was in five middle-income countries by Hoy and Mager, 2021a) and collect data that is representative of the internet population within each country. Consequently, the results provide rigorous insights for a much wider population and arguably have far greater external validity than previous work in these settings.

The second strand of the literature is in relation to a growing body of research about how tax morale impacts tax compliance. Examples of this work in high-income countries include how social norms (Hallsworth, 2014, De Neve et al., 2021), the provision of public goods (Giaccobasso et al., 2022) and a positive outlook on the government (Cullen et al., 2021) influence tax compliance. The aspects of tax morale that matter the most in terms of compliance in developing countries is still unclear (Prichard et al., 2019, Prichard et al., 2022). Outside of Latin America, there has been only a small number of randomized field experiments in developing countries examining the motivations underpinning tax morale, such as a sense of obligation to contribute to public goods or keep up with social norms (Shimeles et al., 2017, Mascagni and Nell, 2022, Collin et al., 2021, Hoy et al., 2021). This study contributes to this field by being the first to examine causally how progressivity (or lack thereof) in the tax and transfer system impacts people’s tax morale across countries. The link between progressivity and tax compliance is widely thought to be first order (e.g., Saez and Zucman, 2020), however, the empirical evidence is very limited (an exception is Besley et al. (2021) who illustrate using an event study that the perceived regressivity of the poll tax in the United Kingdom triggered evasion). The pre-registered, randomized survey experiment in this study was designed to specifically identify how progressivity influences people’s tax morale, which has been a challenge in prior work that relies on administrative data where people’s perceptions are not able to be captured. In this study, the channels driving the treatment effects are isolated by capturing people’s prior beliefs and existing preferences, as well as comparing across countries and treatments. It only became feasible to study this question across developing countries because of the recent release of the CEQ database, which measures the progressivity of taxes and transfers in a standardized way (Lustig et al., 2020). To the best of my knowledge, the closest example of related work is by Stantcheva (2021), who conducts randomized survey experiments focusing on income and estate taxes in the United States and uses detailed educational videos as information treatments to show redistributive considerations influence respondents’ preferences more than efficiency considerations.

This paper is structured as follows. Section 2 provides a conceptual framework and the hypotheses that flow from the theory. Section 3 describes the methodology, including the sample selection, survey design, and provides details about the setting. Section 4 presents the main descriptive and experimental findings. Section 5 discusses the implications of these findings from a theoretical and policy perspective.

## 2 Conceptual framework and hypotheses

### 2.1 Conceptual framework

Traditionally, tax compliance has been conceptualized as a trade-off between the punishment they face from being caught for non-compliance compared to the cost of complying (Allingham and Sandmo, 1972).^6^ This is shown formally in the utility functions below whereby $y_{i}$ is an individual’s household income before tax, d is the probability of being detected as non-compliant, $p_{i}$ is a fixed amount that represents the punishment a taxpayer will face if found to be non-compliant and $t_{i}$ is a fixed amount that represents a taxpayer’s tax obligation. In this simple version of the Allingham and Sandmo (1972) model, taxpayers make a binary decision as to whether or not they will comply (i.e., the extensive margin), as opposed to the amount to which they will comply (i.e., the intensive margin). In recent years this model of tax compliance has been extended to include other factors that drive compliance beyond enforcement and punishment, such as people’s desire to keep in line with social norms (Hallsworth, 2014, Slemrod, 2019, Antinyan and Asatryan, 2019). As such, the traditional model of tax compliance has been broadened to incorporate what is typically referred to as tax morale (Luttmer and Singhal, 2014). Prichard et al. (2019) suggest that other than enforcement, issues to do with the facilitation of tax payments and trust in the tax system impact tax compliance. They further hypothesize that trust in the tax system is built on four related concepts of equity, reciprocity, accountability and fairness. Formally, tax morale can be expressed as the utility gain an individual receives from paying tax $a_{i}$. As such, for a single point in time, an individual’s utility from complying with taxes ($U_{ci}$) and from not complying ($U_{ni}$) can be expressed as:

(1) $$U_{ci}=y_{i}-t_{i}+a_{i}$$

and

(2) $$U_{ni}=y_{i}-dp_{i},$$

According to this model, taxpayers comply if $U_{ci}$ $>U_{ni}$, which requires that:

(3) $$t_{i}<dp_{i}+a_{i}$$

I extend this basic model by decomposing motivations underpinning tax morale (shown as $a_{i}$ in the model above) to specifically identify the influence of “equity” (Prichard et al., 2019). By doing so I separate equity from other motivations underpinning tax morale (shown as $b_{i}$ in the revised model below). Equity, more precisely articulated as vertical equity by Prichard et al. (2019), is considered to be a driver of tax compliance because many people would prefer lower levels of inequality in their country and consequently are supportive of the role taxes and transfers can play in redistributing resources from rich to poor (World Values Survey (WVS), 2022). This is formally integrated into the model by drawing on the “workhorse” utility function by Alesina and Giuliano (2011) that shows how differences between actual and preferred levels of inequality $\left(Q-Q_{i}^{*}\right)$ impact people’s utility (the weight an individual places on deviations from their ideal level of inequality is captured in the term $\gamma_{i}$). The revised model of people’s utility from paying tax can be expressed as follows:

(4) $$U_{ci}=y_{i}-t_{i}+b_{i}-\gamma_{i}{\left(Q-Q_{i}^{*}\right)}^{2}$$

I dis-aggregate this utility function further by continuing to draw on Alesina and Giuliano’s (2011) seminal work as they argue that people’s utility is largely (if not exclusively) influenced by differences between actual and preferred levels of inequality that are due to factors outside an individual’s control $\left(Q^{l}-Q_{i}^{l*}\right)$, as opposed to overall levels of inequality $\left(Q-Q_{i}^{*}\right)$. I identify that one of the key determinants of inequality outside an individual’s control is the degree of progressivity in the tax and transfer system in their country. I reflect this in the model with the term $\left(Q^{t}-Q_{i}^{t*}\right)$, whereby $Q^{t}$ is the level of progressivity in the tax and transfer system, $Q_{i}^{t*}$ is people’s preferred levels of progressivity in the tax and transfer system, and $\gamma_{i}^{t}$ reflects the weighting people place on this (all other differences in inequality are captured in the terms denoted with o). As such, holding everything else constant, people who prefer the existing level of progressivity in the tax and transfer system will be more willing to comply with taxes than those who do not. Consequently, the revised model of people’s utility from paying tax can be expressed as follows:

(5) $$U_{ci}=y_{i}-t_{i}+b_{i}-\gamma_{i}^{t}{\left(Q^{t}-Q_{i}^{t*}\right)}^{2}-\gamma_{i}^{o}{\left(Q^{o}-Q_{i}^{o*}\right)}^{2}$$

The final substantive modification I make is to incorporate the fact that it is people’s beliefs about how taxes and transfers are distributed, as opposed to what is the case, that will influence their tax morale. Previous research has shown that people tend to have a poor understanding of both the level of inequality in their country and their position in the national income distribution (see, for example, Hoy and Mager, 2021a, Hoy and Mager, 2021b) and there is evidence from the United States to suggest these misperceptions also extend to tax policies (Stantcheva, 2021). Consequently, I rewrite the utility function to factor in that people’s tax morale will be influenced by the extent to which they believe the tax and transfer system is progressive $\left(Q_{b_{i}}^{t}\right)$:

(6) $$U_{ci}=y_{i}-t_{i}+b_{i}-\gamma_{i}^{t}{\left(Q_{bi}^{t}-Q_{i}^{t*}\right)}^{2}-\gamma_{i}^{o}{\left(Q_{bi}^{o}-Q_{i}^{o*}\right)}^{2}$$

This utility function provides guidance as to how people’s utility ($U_{ci}$) will be influenced by accurate information (I) about the progressivity of taxes and/or transfers in their country ($Q^{t}$). In other words, it is possible to make predictions about how people’s utility from paying taxes varies when they have accurate information (i.e., $U_{ci}|I$). The two main dimensions in which heterogeneity would be expected are in terms of people’s prior beliefs and existing preferences about the progressivity of tax and transfer policies (captured formally as ($Q^{t}-Q_{bi}^{t}$) and ($Q^{t}-Q_{i}^{t*}$) respectively). These dimensions form the basis of the primary hypotheses that are discussed in the following subsection.

### 2.2 Hypotheses

Three groups of primary hypotheses emerge from the conceptual framework. Group A of Hypotheses is based on a key implication from the theory and existing empirical literature suggesting that progressivity (a lack of progressivity) in the tax and transfer system will, on average, lead to higher (lower) levels of tax morale. Group B of Hypotheses summarizes how people’s tax morale is likely to vary by their prior beliefs about the progressivity of the tax and transfer system. Group C of Hypotheses outlines how people’s tax morale is expected to vary by their preferences for progressivity in the tax and transfer system. All of these hypotheses were pre-registered on the American Economic Association RCT Registry before field work commenced (Hoy, 2022).

**Group A – People’s tax morale varies by the degree of progressivity in the tax and transfer system**

Hyopthesis A1 Informing people that the distribution of taxes and/or transfers is progressive, will increase their tax morale.

Hyopthesis A2 Informing people that the distribution of taxes and/or transfers is not progressive, will decrease their tax morale.

**Group B – People’s tax morale varies by their prior beliefs about the progressivity of the tax and transfer system**

Hyopthesis B1 Informing people that the distribution of taxes and/or transfers is progressive when they thought it was not progressive, will increase their tax morale.

Hyopthesis B2 Informing people that the distribution of taxes and/or transfers is not progressive when they thought it was progressive, will decrease their tax morale.

**Group C – People’s tax morale varies by their preferences for the progressivity of the tax and transfer system**

Hyopthesis C1 Informing people that the distribution of taxes and/or transfers is progressive when they prefer it to be progressive, will increase their tax morale.

Hyopthesis C2 Informing people that the distribution of taxes and/or transfers is not progressive when they prefer it to be progressive, will decrease their tax morale.

These hypotheses do not focus on differences between how the treatments may impact tax morale, but ex-ante it is conceivable differences would exist. As noted in the pre-analysis plan, survey respondents may be more likely to respond to the taxes treatment than to the transfers treatment for several reasons. Firstly, on average, the share of household income collected in taxes is much higher than what is provided in transfers, which means people may be more concerned about how taxes are distributed compared to transfers. Secondly, there is reason to believe that “loss aversion” could exist where people’s utility is more likely to be influenced by “losing” from paying tax than by “gaining” from receiving a transfer. Thirdly, people’s awareness of when they pay taxes may be higher than their awareness about when they receive a transfer. For example, people are likely to be more conscious of paying income tax compared to receiving a subsidy for their fuel consumption, and consequently, this could make them more responsive to information about who pays taxes as opposed to who receives transfers.

## 3 Methodology

### 3.1 Setting of the study

This study focuses on eight countries (Colombia, Ghana, Indonesia, Jordan, Mexico, Sri Lanka, South Africa, and Tanzania) where the extent of progressivity in the tax and transfer system varies considerably. The difference between the gross and net GINI index ranges from around half a percentage point in Sri Lanka to almost ten percentage points in South Africa (see Figure A1). These countries were selected for several reasons (see Appendix A), the most important of which is the availability of standardized, cross-country comparable data about the progressivity of taxes and government transfers. The largest effort that has been made to collect and disseminate this data has been through the Commitment to Equity (CEQ) Institute at Tulane University, which is headed by Nora Lustig (Commitment to Equity (CEQ) Institute, 2021). Importantly, this CEQ data takes into account the actual behavior of households and consequently presents the de facto distribution of taxes and transfers. As such, this factors in the degree of tax compliance and the accuracy of targeting of transfers. These estimates are based on standardized household income and expenditure surveys, and in 2020, a cross-country database that provided dis-aggregated information in a standardized way for many countries was publicly released as part of monitoring progress towards Sustainable Development Goal (SDG) 10.4 (Lustig et al., 2020).

**Fig. 1:**
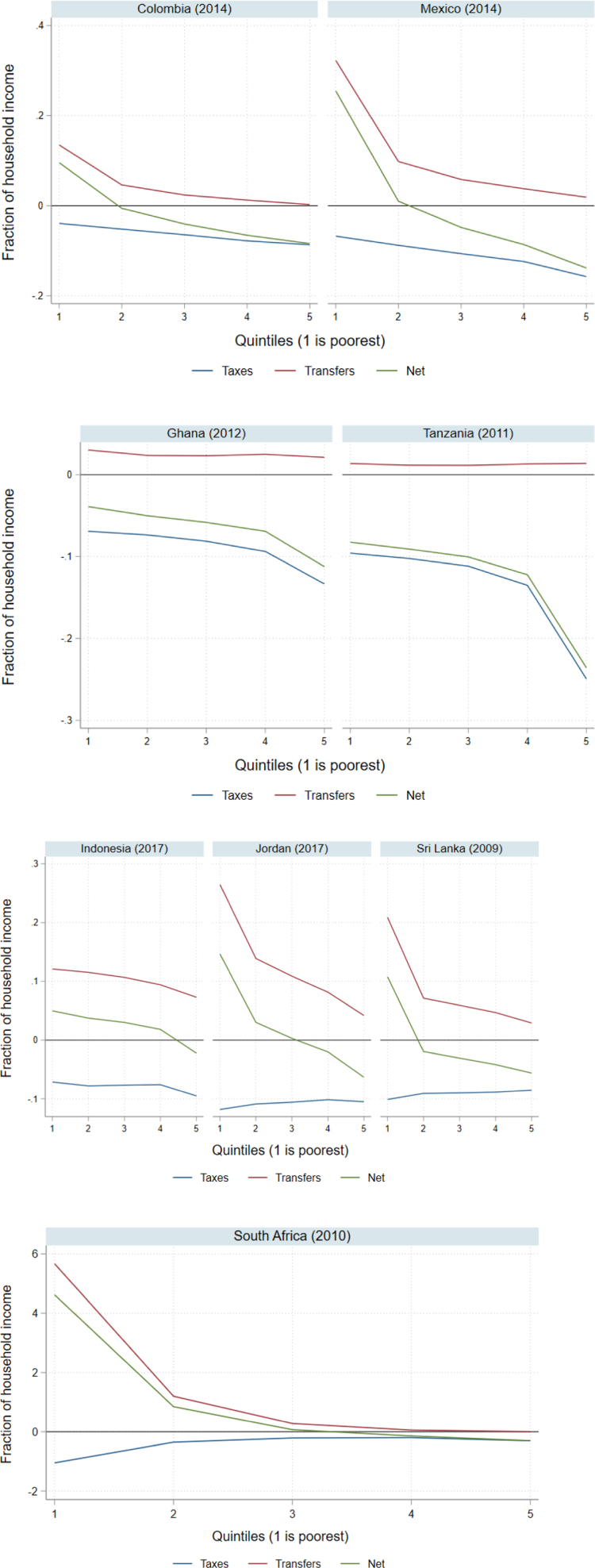
Taxes and government transfers (both direct and indirect) as a fraction of household income. Note: This figure shows the average fraction of household income for each quintile in each country that is directly or indirectly paid in taxes or received in government transfers as well as the net impact of taxes and transfers on household income. For presentational purposes, this figure shows the distribution of taxes and/or transfers across quintiles (whereas the CEQ database focuses on deciles) and combines both direct and indirect taxes and government transfers. Taxes are displayed as negative because they reduce household income. In South Africa, taxes and transfers as a fraction of household income is greater than 1 for the poorest quintile. This is possible because household consumption is higher than household income for the these households.

Source: CEQ, 2021

The tax system is progressive in four of the eight countries (Colombia, Mexico, Ghana and Tanzania) and the transfer system is progressive in six of the eight countries (Colombia, Mexico, Indonesia, Jordan, Sri Lanka and South Africa).^7^ The net impact of taxes and transfers is “weakly” progressive in all countries; however, in Ghana and Tanzania, the net impact is negative across all quintiles (i.e., all households pay more in tax than they receive in transfers). As such, for this study, the net impact of taxes and transfers is only considered to be progressive in the six countries (Colombia, Mexico, Indonesia, Jordan, Sri Lanka, and South Africa) where poorer households receive more in transfers than they pay in taxes. In summary, there are three groups of countries, one where both taxes and transfers are progressive (Colombia and Mexico), another where taxes are progressive and transfers are not (Ghana and Tanzania), and a final group where taxes are not progressive and transfers are (Indonesia, Jordan, Sri Lanka and South Africa) (see Fig. 1).

### 3.2 Sample selection and sample size

The randomized survey experiment collected a broadly representative sample of the population with internet access in each country during the first three months of 2022 using an internationally respected online survey firm, RIWI (see Appendix A for details about the survey methodology). Alesina et al., 2018, Alesina et al., 2022 also use online randomized survey experiments of a representative sample of internet users in their seminal work in the United States and Western Europe. A key difference is that in high-income countries, internet access is nearly universal, whereas in the developing countries in this study, the internet penetration rate varies from 20 to 67 percent of the total population (World Bank, 2021). To address concerns about how representative the sample is of the total population, throughout the body of the paper I weight the descriptive and experimental results by the age and gender of the total population. As a robustness check, I present the sample average treatment effects (i.e., the unweighted findings) in Appendix B (see Table A3 in Appendix B). In general, the effects are almost identical.

The sample size in each of the eight countries is at least 3600 respondents who completed the survey. In total, over 30,000 respondents participated in the randomized survey experiment. As per the pre-registration, the sample size of at least 900 respondents in each treatment and the control group in each country was determined based on having adequate statistical power to detect heterogeneous treatment effects between respondents depending on their prior beliefs and existing preferences for progressivity. This sample size is also consistent with similar cross-country survey experiments (e.g., Alesina et al., 2022) and best practices in the existing literature (Haaland et al., 2023).

### 3.3 Survey design

The survey consisted of two sections and the treatments were provided in between the two sections of the survey. The first collected people’s demographic characteristics as well as prior beliefs and existing preferences. In this section, respondents were asked to state where they perceived their household to be in the national income distribution (as opposed to reporting household income) as typically people’s perception of their relative position is more strongly correlated with their policy preferences (Hoy and Mager, 2021a). The second section included questions about people’s tax morale. The survey was designed to be quite focused and brief, which enabled the median respondent to complete the entire survey in less than 11 min. The survey instrument in English is provided in full in Appendix C and the exact treatments in each country are provided in Appendix D.^8^

To maximize the likelihood that respondents would provide honest answers, at the start of the survey they were informed that the answers they provide would be restricted to a team of independent, non-partisan researchers and that they would remain entirely anonymous. Ensuring respondent anonymity was an essential part of the study as this is a sensitive topic (respondents were effectively being asked to indirectly self-report their criminal behavior). This meant that no identifying information whatsoever was collected. As a result, it was not possible to conduct a follow-up survey as this would have required respondents to provide details about how to be contacted and consequently reveal their identity. This trade-off between maintaining anonymity versus being able to recontact respondents for follow-up surveys is unavoidable. Given that similar, cross-country randomized survey experiments have consistently shown that follow-up surveys detect persistent treatment effects, there is no reason to believe that the same would not occur in this case.

#### 3.3.1 Questions measuring people’s tax morale

People’s tax morale was measured using standardized questions from cross-country surveys (e.g., Afrobarometer) as well as drawing on the experience of previous survey instruments focusing on measuring tax morale in developing countries (e.g., those referred to in Prichard et al., 2019). There is no single “ideal” question on this complex topic. To the best of my knowledge, there is yet to be a study that systematically uses tax administrative data alongside survey data to measure which exact questions better correlate with actual tax compliance behavior. Rather there is a general acceptance in the literature that a multifaceted conceptualization of tax morale (which can be measured by survey questions) provides a plausible, but far from perfect, proxy for compliance (e.g., see discussion in Besley et al., 2021 and Luttmer and Singhal, 2014). As a result, five questions focusing on slightly different ways of measuring people’s tax morale were used in this survey experiment. This ensured that if a treatment effect was detected across most or even all of these questions, there would be more reason to believe that progressivity impacts tax morale in general.^9^

Consistent with best practices in the literature, I aggregate the five measures of tax morale into an index (whereby each question receives equal weighting). This is the identical approach to what was used in related randomized survey experiments by Alesina et al. (2018) and Karadja et al. (2017). Specifically, I create a “Tax Morale” Index, which is an unweighted average of the Z-scores of all five outcome variables, oriented so that a higher index means more tax morale. The answers to each of the tax morale questions and the “Tax Morale” Index are presented in the tables of results. The focus of the discussion of the results is on the general pattern across the various measures of tax morale, summarized in the tax morale index, as opposed to an in-depth analysis of differences between each of the questions. This is primarily because it is not obvious any question better reflects people’s tax morale more than another, which is why as was stated clearly in the pre-registration of this study, I do not focus on differences between specific outcome variables.

#### 3.3.2 Treatments

The treatments were designed to provide people with accurate information about the progressivity of taxes and/or government transfers in their country. This means that the treatments provided either positive or negative information about progressivity depending on the actual circumstances in their country.^10^ Specifically, the treatments provided an indication of whether taxes and/or transfers were progressive in their country but did not explicitly provide information about the level of taxes and transfers as a percentage of household income. This is because it would not be possible to isolate the channels through which the treatments were impacting people’s tax morale if the information was provided about both the progressivity and level of taxes and transfers.^11^ It is theoretically possible that a respondent who is very well informed about their own experience with taxes and/or transfers and their position in the income distribution may interpret the relative magnitude of the bars in the treatment image to “back out” the exact level of taxes and transfers. However, this seems very unlikely given the treatments provided information about the total tax burden (i.e., not just personal income taxes), total transfer benefit, or net impact of fiscal policies, which are not straightforward for an individual to calculate. In addition, most respondents were unable to accurately select their position in the income distribution (consistent with Hoy and Mager, 2021a), which means it is highly improbable that they could correctly back out the exact level of taxes and transfers as a percentage of household income.

Survey respondents in each country were randomly allocated either to one of three treatment groups or to a control group that received no information (i.e., the multiple treatment arms were exclusive of one another). The first treatment (hereafter the “taxes treatment”) provided information from the CEQ database about the distribution of taxes (both direct and indirect taxes, such as income tax and value-added tax) in their country. The second treatment (hereafter the “transfers treatment”) provided information from the CEQ database about the distribution of government transfers (both direct and indirect transfers, such as cash payments and energy subsidies). The third treatment (hereafter the “combined treatment”) provided information from the CEQ database about the net effect of the distribution of taxes and government transfers.

### 3.4 Empirical analysis

I conducted a randomized survey experiment to test the impact of accurate information about the distribution of taxes, government transfers or both on people’s tax morale. Randomization allows for the impact of the treatments to be determined by comparing differences in mean outcomes between the control group and treatment groups. The randomization process was stratified by the age and sex of respondents. The balance tables for each country based on all answers provided before the treatment are in Appendix B (see Tables A6–A8), including measures of both individual and joint significance (i.e., both t-statistics for every variable and an f-statistic across all variables within a given country).

The main results of the survey experiment are based on pooled Ordinary Least Squares (OLS) regressions with country fixed effects across all countries for which the treatment is in the same direction. This approach is in line with what was undertaken by Alesina et al., 2018, Alesina et al., 2022. For example, respondents across the four countries for which taxes were progressive (Colombia, Ghana, Mexico, and Tanzania) are pooled together, and respondents across the four countries for which taxes were not progressive (Indonesia, Jordan, Sri Lanka, and South Africa) are pooled together. As the groups of countries for which the treatment was in the same direction varied across the treatments (e.g., four countries had progressive taxes, while 6 countries had progressive transfers), the main regression analysis was conducted solely between a specific treatment group and the control group. Specifically, an OLS regression in the form of a linear probability model was conducted by creating a dummy variable for each outcome of interest (see Section 3.2.2 and Appendix A for details) and a dummy variable for a specific treatment group that takes on the value one if the respondent belongs to the specific treatment group and the value zero if the respondent belongs to the control group. This can be expressed formally as follows:

(7) $$Y_{ij}=\beta_{0}+\beta_{1}T+X_{i}\gamma +\theta_{j}+\epsilon_{ij}$$

where i denotes individuals, j denotes countries, $\beta_{1}$ captures the average difference between respondents in a specific treatment group (T) and the control group about the outcome of interest (Y). Further, $X_{i}$ is a vector of variables that controls for potential imbalances in background characteristics (age, gender, location, education level, device type, employment status, perceived relative income) between treatment and control groups, $\theta_{j}$ captures country level fixed effects, $\epsilon_{ij}$ is the model error term (clustered at the country level) and $\beta_{0}$ is the intercept.

As per the pre-registration, heterogeneous effects of the treatments are explored by conducting the regression analysis outlined in Eq. (7) on subsets of respondents based on their responses provided before the treatments. Specifically, Group B of hypotheses is tested by conducting the regression analysis outlined in Eq. (7) on respondents who believed the tax system was progressive and then separately reproducing this analysis on respondents who did not believe the tax system was progressive. Group C of Hypotheses is tested by conducting the regression analysis outlined in Eq. (7) on respondents who prefer the tax system to be progressive and then separately reproducing this analysis on respondents who do not prefer the tax system to be progressive.

**Fig. 2:**
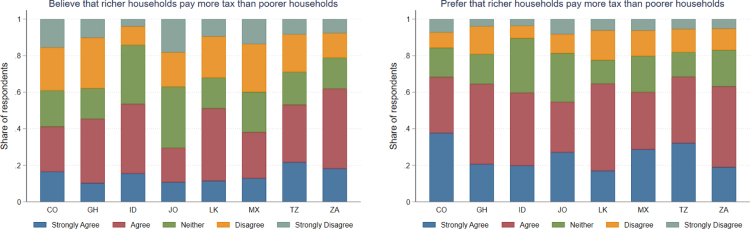
Beliefs and preferences regarding the progressivity of taxes across countries. Note: This figure shows the share of respondents stating they had a prior belief and/or an existing preference that the tax system is progressive in their country. *CO:* Colombia. *GH:* Ghana. *ID:* Indonesia. *JO:* Jordan. *LK:* Sri Lanka. *MX:* Mexico. *TZ:* Tanzania. *ZA:* South Africa. Beliefs about progressivity are based on Q8, which asks respondents whether they believe that richer households pay a higher share of their income in tax than poorer households. Preferences about progressivity are based on Q9, which asks respondents whether they think that richer households should pay a higher share of their income in tax than poorer households.

## 4 Findings

### 4.1 Descriptive findings

On average, almost two-thirds of respondents across the eight countries in this study stated that they prefer richer households to pay a higher share of their income in tax than poorer households, but less than half stated that they believed this was currently the case. In addition, another 15 to 30 percent of respondents stated that they neither agreed nor disagreed with these statements. Fig. 2 shows for each country the share of respondents who stated they currently believe the tax system is progressive and those who would prefer this to be the case (this question was asked to all respondents before the treatments). Across countries, around one-third of respondents stated a difference between what they believe to be the case and what they would prefer to exist. Respondents in Jordan were the least likely to believe that the tax system was progressive (30 percent), and those in South Africa were the most likely (62 percent). Across these eight countries, people’s beliefs and preferences about whether the tax system was progressive were largely unrelated to what is the case. Believing the tax system was progressive was positively associated with having lower levels of education, preferring lower inequality and perceiving one’s household as a net contributor to the tax and transfer system (see multivariate regression analysis in Table A11 in Appendix B). These descriptive trends suggest people have a limited understanding of how progressive the tax system is in their country, but regardless of people’s beliefs, a sizable majority of people would prefer to have a progressive tax system.

Preferences for progressive taxes and transfers varied somewhat, but not dramatically, across respondents based on where they perceived themselves to be in the national income distribution. On average, richer respondents were less supportive of progressive taxation by five to ten percentage points, but still, in all countries, in every quintile, more respondents agreed than disagreed that richer households should pay a higher share of their income in tax than poorer households (see Figure A2 in Appendix B).^12^ Multivariate regression analysis controlling for other background characteristics also shows that perceiving oneself as rich is somewhat negatively associated with supporting progressive taxes in each country (see Table A12 in the Appendix). Preferring lower inequality, paying a larger share of household income in tax and perceiving one’s household as a net contributor to the tax and transfer system were also positively associated with preferring progressive taxes (see Table A12 in the Appendix). Support for progressive transfers was higher and more consistent across the income distribution in each country (see Figure A3 in Appendix B). Collectively, these results imply that there is only limited hostility towards progressive taxes and transfers among people who perceive themselves to be rich. On the other hand, there is far from universal support for progressive taxes and transfers among people who perceive themselves to be poor.^13^ Further descriptive results are presented in Appendix A.

**Table 1:** Overall effects of the treatments.

	Direct	Punishable	Important	Right to Tax	Don’t Refuse	INDEX
	b/se/p	b/se/p	b/se/p	b/se/p	b/se/p	b/se/p
Taxes (Progressive)	0.008	0.022	0.023**	0.016	0.013	0.036**
	(0.01)	(0.02)	(0.01)	(0.01)	(0.01)	(0.01)
p-value	0.594	0.272	0.032	0.140	0.255	0.030
Control group mean	0.569	0.421	0.773	0.596	0.441	0
Observations	7605	7605	7605	7605	7605	7605

Taxes (Not Progressive)	−0.022	−0.022*	−0.012	−0.027	−0.028	−0.048**
	(0.01)	(0.01)	(0.02)	(0.01)	(0.02)	(0.01)
p-value	0.213	0.061	0.517	0.131	0.328	0.011
Control group mean	0.513	0.354	0.692	0.488	0.327	0
Observations	7435	7435	7435	7435	7435	7435

Transfers (Progressive)	−0.006	0.009	0.016	−0.002	0.004	0.009
	(0.01)	(0.01)	(0.01)	(0.02)	(0.01)	(0.02)
p-value	0.569	0.461	0.259	0.914	0.611	0.653
Control group mean	0.536	0.373	0.685	0.475	0.328	0
Observations	11318	11318	11318	11318	11318	11318

Transfers (Not Progressive)	−0.014	0.007	0.002	−0.002	−0.008	0.000
	(0.01)	(0.01)	(0.01)	(0.03)	(0.01)	(0.01)
p-value	0.358	0.652	0.853	0.966	0.423	0.976
Control group mean	0.556	0.434	0.889	0.767	0.571	0
Observations	3810	3810	3810	3810	3810	3810

Combined (Progressive)	0.000	0.008	0.021**	0.020	0.012*	0.025*
	(0.01)	(0.01)	(0.01)	(0.01)	(0.00)	(0.01)
p-value	0.997	0.479	0.049	0.101	0.052	0.098
Control group mean	0.536	0.373	0.686	0.475	0.328	0
Observations	11066	11066	11066	11066	11066	11066

Combined (Not Progressive)	−0.011	−0.024	0.012	0.009	−0.021	−0.010
	(0.02)	(0.01)	(0.01)	(0.03)	(0.02)	(0.02)
p-value	0.714	0.217	0.354	0.806	0.405	0.732
Control group mean	0.556	0.433	0.890	0.766	0.569	0
Observations	3769	3769	3769	3769	3769	3769

Note: This table shows the overall impact of each of the treatments relative to the control group, where countries are pooled based on whether the tax and/or transfer system is progressive. This table is based on Eq. (7) in Section 3 of the paper. Robust standard errors are in brackets. * p<0.1, ** p<0.05, *** p<0.01. *Direct:* Based on Q14, which asks whether respondents would not pay tax if they knew they would not get caught (variable takes value of 0 if they select “Strongly Agree” or ‘Agree” and 1 otherwise). *Punishable:* Based on Q15, which asks respondents their views about people not paying tax (variable takes value of 1 if they select “This is wrong and punishable” and 0 otherwise). *Important:* Based on Q16, which asks respondents whether it is important for people to pay tax (variable takes value of 1 if they select “Strongly Agree” or “Agree” and 0 otherwise). *Right to Tax:* Based on Q17, which asks respondents whether the government always has a right to make people pay tax (variable takes value of 1 if they select “Strongly Agree” or “Agree” and 0 otherwise). *Do not Refuse:* Based on Q18, which asks whether people should refuse to pay taxes until they receive more government transfers (variable takes value of 1 if they select “Strongly Disagree” or “Disagree” and 1 otherwise). *INDEX:* An unweighted average of the Z-scores of all five outcome variables, oriented so that a higher index means higher tax morale.

### 4.2 Main experimental results

#### 4.2.1 Overall effects of each of the treatments

The overall impact of the taxes treatment illustrates that people’s tax morale is influenced by whether or not the tax system is progressive (this is consistent with Hyopthesis A1, Hyopthesis A2). Table 1 shows that respondents who received the taxes treatment in the four countries for which taxes were progressive (Colombia, Ghana, Mexico, and Tanzania) reported higher tax morale. In contrast, respondents who received the taxes treatment in the four countries for which taxes were not progressive (Indonesia, Jordan, Sri Lanka, and South Africa) reported lower tax morale. The negative treatment effect on tax morale in Indonesia, Jordan, Sri Lanka, and South Africa was larger than the positive treatment effect on tax morale in Colombia, Ghana, Mexico, and Tanzania.^14^ The results of the taxes treatment across each of the outcome variables were in the order of one to three percentage points, and the tax morale index for the tax treatment group was 0.036 (0.048) standard deviations higher (lower) in countries where taxes were progressive (not progressive). Weaker (and often insignificant) results were attained from the combined treatment, although the point estimates were still consistent with respondents who received information that the system was progressive (not progressive) reporting higher (lower) tax morale. The transfers treatment had negligible impact on respondents’ tax morale, and, consequently the remaining presentation of the results in the body of the paper does not focus on this treatment (see Table A13 in the Appendix for a presentation of the heterogeneous effects of the transfers treatment).

**Fig. 3:**
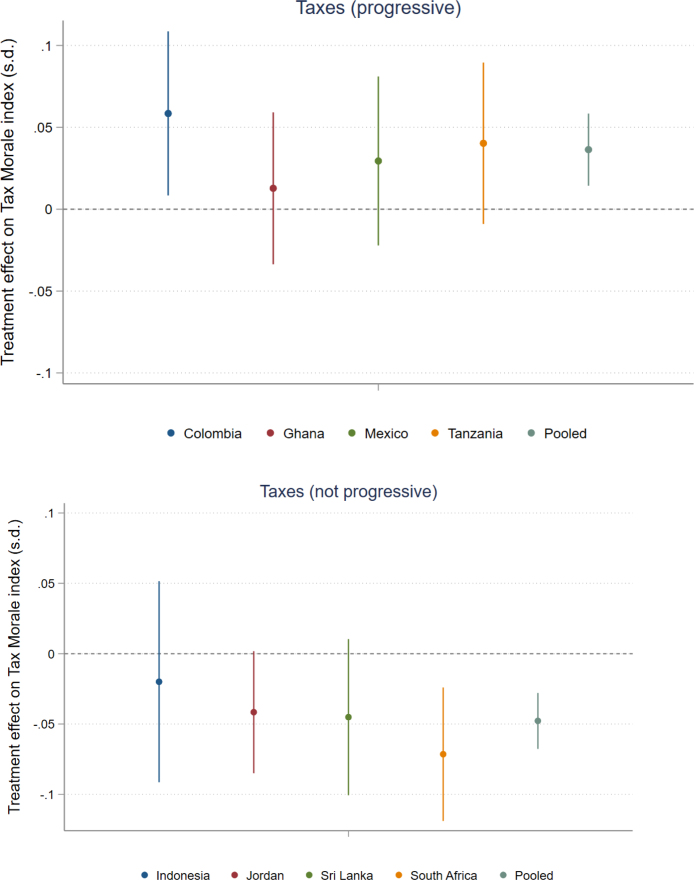
Overall impact of the tax treatment in each country. Note: This figure shows the overall impact of the tax treatment in each country. These results are based on Eq. (7) in Section 3 of the paper, however country fixed effects are not included. 90 percent confidence intervals are displayed in this figure. *INDEX:* An unweighted average of the Z-scores of all five outcome variables, oriented so that a higher index means more Tax morale.

The main findings of the taxes treatment did not vary greatly across countries, which means the pooled regression results discussed above are not driven by a small number of countries. Fig. 3 shows how the overall treatment effects were somewhat similar across countries based on the tax morale index. The exceptions are Ghana and Indonesia, where the treatment effects were smaller, but the point estimates are still in the same direction. These country-level results further illustrate the robustness of the main finding of this study, which is that a desire for progressive taxes is linked to people’s tax morale (see country-level results for each treatment in Table A14 in Appendix B).

#### 4.2.2 Heterogeneous effects of the taxes treatment based on prior beliefs and existing preferences

The impact of the taxes treatment varied based on people’s prior beliefs. Table 2 shows that the overall negative effect of the taxes treatment in countries where the tax system was not progressive is almost entirely driven by respondents who held a prior belief that the tax system was progressive. The tax morale index for the taxes treatment group was 0.083 standard deviations lower among this subset of respondents. As such, the taxes treatment appears to be correcting people’s prior beliefs, and this impacts their tax morale in the expected direction in this instance (i.e., consistent with Hypothesis B2 in Section 2). However, there was no clear evidence in favor of Hypothesis B1 for the taxes treatment, as respondents who held a prior belief that the tax system was not progressive were not significantly more willing to pay tax when they were informed that taxes were progressive.

The impact of the taxes treatment also varied by people’s existing preferences. Table 2 shows that the overall effects of the taxes treatment were almost entirely driven by respondents who held an existing preference for progressivity. The tax morale index for the taxes treatment group in countries where taxes were progressive (not progressive) was 0.050 (0.066) standard deviations higher (lower) among this subset of respondents. As such, the taxes treatment appears to be impacting people’s tax morale in the expected direction in these instances (i.e., consistent with Hyopthesis C1, Hyopthesis C2 in Section 2). There were no notable trends in terms of heterogeneous effects from the taxes treatment across other dimensions that were included in the pre-analysis plan (e.g., by respondents’ perceived place in the national income distribution). These results are discussed further in Appendix A.

**Table 2:** Heterogeneous effects of the taxes treatment based on prior beliefs and existing preferences.

	Direct	Punishable	Important	Right to Tax	Do not Refuse	INDEX
	b/se	b/se	b/se	b/se	b/se	b/se
**Panel A - Respondents in countries where taxes were progressive**						
Believe progressive × Treated	0.017	0.037**	0.008	0.016	0.039	0.053
	(0.01)	(0.01)	(0.01)	(0.03)	(0.02)	(0.03)
Believe not progressive × Treated	0.002	0.011	0.033	0.013	−0.007	0.022
	(0.02)	(0.02)	(0.01)	(0.02)	(0.02)	(0.02)
p-value difference	0.376	0.253	0.423	0.950	0.294	0.553
Observations	7605	7605	7605	7605	7605	7605
Prefer progressive × Treated	0.019	0.030	0.026*	0.014	0.025	0.050***
	(0.01)	(0.02)	(0.01)	(0.02)	(0.01)	(0.01)
Prefer not progressive × Treated	−0.013	0.005	0.010	0.013	−0.007	0.004
	(0.02)	(0.02)	(0.02)	(0.01)	(0.01)	(0.02)
p-value difference	0.051	0.217	0.590	0.946	0.036	0.153
Observations	7605	7605	7605	7605	7605	7605

**Panel B - Respondents in countries where taxes were not progressive**						
Believe progressive × Treated	−0.020	−0.047**	−0.039	−0.063**	−0.027	−0.083**
	(0.03)	(0.01)	(0.02)	(0.01)	(0.04)	(0.02)
Believe not progressive × Treated	−0.024	0.001	0.012	0.005	−0.030	−0.016
	(0.02)	(0.01)	(0.02)	(0.02)	(0.01)	(0.02)
p-value difference	0.915	0.010	0.096	0.010	0.908	0.110
Observations	7435	7435	7435	7435	7435	7435
Prefer progressive × Treated	−0.023	−0.039***	−0.030	−0.046*	−0.018	−0.066***
	(0.02)	(0.00)	(0.02)	(0.02)	(0.03)	(0.01)
Prefer not progressive × Treated	−0.021	0.005	0.012	−0.003	−0.043	−0.021
	(0.03)	(0.01)	(0.02)	(0.02)	(0.02)	(0.03)
p-value difference	0.954	0.028	0.167	0.247	0.410	0.348
Observations	7435	7435	7435	7435	7435	7435

Note: This table shows the heterogeneous effects of the taxes treatment based on respondents prior beliefs about and existing preferences regarding whether taxes were progressive, where countries are pooled based on whether or not the tax is actually progressive. This table is based on Eq. (7) in Section 3 of the paper, except the regression analysis is conducted separately for respondents based on their prior beliefs and existing preferences. Robust standard errors are in brackets. * p<0.1, ** p<0.05, *** p<0.01. Beliefs about progressivity are based on Q8, which asks respondents whether they believe that richer households pay a higher share of their income in tax than poorer households. Preferences about progressivity are based on Q9, which asks respondents whether they think that richer households should pay a higher share of their income in tax than poorer households. See the notes to Table 1 for further variable definitions.

**Table 3:** Heterogeneous effects of the combined treatment based on prior beliefs and existing preferences.

	Direct	Punishable	Important	Right to Tax	Do not Refuse	INDEX
	b/se	b/se	b/se	b/se	b/se	b/se
**Panel A - Respondents in countries where combined effect of taxes and transfers was progressive**						
Believe progressive × Treated	−0.003	−0.003	−0.005	−0.012	0.015	−0.004
	(0.02)	(0.01)	(0.01)	(0.01)	(0.01)	(0.01)
Believe not progressive × Treated	0.002	0.016	0.042***	0.048**	0.009	0.049**
	(0.02)	(0.02)	(0.01)	(0.02)	(0.01)	(0.02)
p-value difference	0.848	0.316	0.003	0.011	0.804	0.038
Observations	11066	11066	11066	11066	11066	11066
Prefer progressive × Treated	0.010	0.002	0.007	0.017	0.027*	0.027**
	(0.01)	(0.01)	(0.00)	(0.02)	(0.01)	(0.01)
Prefer not progressive × Treated	−0.016	0.017	0.043*	0.026	−0.014	0.023
	(0.02)	(0.02)	(0.02)	(0.02)	(0.02)	(0.04)
p-value difference	0.348	0.557	0.137	0.749	0.285	0.929
Observations	11066	11066	11066	11066	11066	11066

**Panel B - Respondents in countries where combined effect of taxes and transfers was not progressive**						
Believe progressive × Treated	−0.035	−0.048	0.010	0.026	−0.040	−0.030
	(0.01)	(0.02)	(0.00)	(0.02)	(0.03)	(0.02)
Believe not progressive × Treated	0.015	0.002	0.010	−0.013	0.000	0.009
	(0.04)	(0.01)	(0.02)	(0.04)	(0.00)	(0.03)
p-value difference	0.385	0.363	0.990	0.308	0.383	0.161
Observations	3769	3769	3769	3769	3769	3769
Prefer progressive × Treated	−0.007	−0.028	0.011	0.011	−0.032	−0.015
	(0.01)	(0.02)	(0.00)	(0.02)	(0.01)	(0.01)
Prefer not progressive × Treated	−0.017	−0.015	0.009	−0.003	0.008	−0.003
	(0.04)	(0.02)	(0.01)	(0.06)	(0.02)	(0.05)
p-value difference	0.779	0.820	0.932	0.771	0.037	0.816
Observations	3769	3769	3769	3769	3769	3769

Note: This table shows the heterogeneous effects of the combined treatment based on respondents prior beliefs about and existing preferences regarding whether taxes were progressive, where countries are pooled based on whether or not the combined effect of taxes and transfers is actually progressive. This table is based on Eq. (7) in Section 3 of the paper, except the regression analysis is conducted separately for respondents based on their prior beliefs and existing preferences. Robust standard errors are in brackets. * p<0.1, ** p<0.05, *** p<0.01. Beliefs about progressivity are based on Q8, which asks respondents whether they believe that richer households pay a higher share of their income in tax than poorer households. Preferences about progressivity are based on Q9, which asks respondents whether they think that richer households should pay a higher share of their income in tax than poorer households. See the notes to Table 1 for further variable definitions.

#### 4.2.3 Heterogeneous effects of the combined treatment based on prior beliefs and existing preferences

The impact of the combined treatment varied based on people’s prior beliefs. Table 3 shows that the overall effects of the combined treatment were entirely driven by respondents who received information counter to their prior briefs. Among these respondents, the tax morale index for the combined treatment group in countries where taxes were progressive (not progressive) was 0.049 (0.030) standard deviations higher (lower). As such, the combined treatment appears to be correcting people’s prior beliefs and this impacts their tax morale in the expected direction in these instances (i.e., consistent with Hyopthesis B1, Hyopthesis B2 in Section 2). However, there is no evidence to suggest the treatment varied based on whether or not respondents held an existing preference for progressivity (i.e., there is no support for Hyopthesis C1, Hyopthesis C2 in Section 2).

There were no notable trends in terms of heterogeneous effects from the combined treatment across other dimensions that were included in the pre-analysis plan (e.g., by respondents’ perceived place in the national income distribution). These results are discussed further in Appendix A.

## 5 Discussion

### 5.1 Summary of the experimental results

This study has illustrated that progressivity in the tax system influences tax morale. Respondents who were informed that the tax system was progressive (not progressive) reported higher (lower) levels of tax morale. The negative effect on tax morale from being informed that the tax system was not progressive appears to be larger than the positive effect on tax morale from being informed taxes are progressive. However, as this is across different subsets of countries, it is not directly comparable. There were weaker overall effects from the combined treatment (although the point estimates were in the same direction) and no impact from the transfers treatment. The main experimental results were predominantly driven by respondents in cases where the information they received was counter to their prior beliefs and/or consistent with their preferences. There were no notable trends in terms of heterogeneous treatment effects across other dimensions that were included in the pre-analysis plan (e.g., by respondents’ perceived place in the national income distribution). As such, these results suggest that people’s beliefs about and preferences for the progressivity of taxes matter more – in terms of driving tax morale responses to information about progressivity – than more traditional factors related to individual incentives, such as their position in the income distribution.

There were some differences between the impact of the treatments across specific questions measuring people’s tax morale, but these differences should be interpreted with caution. Differences between measures of people’s tax morale have been observed in the existing literature (Prichard, forthcoming) and this was noted as likely to occur in the pre-analysis plan. As a result, five questions focusing on slightly different ways of measuring people’s tax morale were used in this survey experiment. The consistency of the effect of the taxes treatment across most of the questions illustrates the robustness of the findings of the randomized survey experiment.

The differences between the size of the effects of the taxes and other treatments were somewhat anticipated as noted in Section 2 and in the pre-analysis plan.^15^ The most straightforward explanation for these differences is that the questions were about the tax system, and, consequently people were more responsive to information about taxes than transfers. In other words, respondents’ elasticity of tax morale is higher for information about taxes. Another potentially compatible explanation for these results is that people may not necessarily link the taxes they pay with the transfers people receive. It may not be clear to respondents that the structure and generosity of the transfer system have anything to do with paying taxes.

### 5.2 Theoretical implications from this study

This study has generated rigorous evidence that the progressivity of the tax system shapes people’s tax morale across countries. As discussed throughout this paper, before this study there was limited empirical evidence about how the progressivity of taxes and government transfers shapes people’s tax morale, particularly in developing countries. The results provide clear evidence supporting a conceptual framework that combines seminal theoretical models of tax compliance (Allingham and Sandmo, 1972) and preferences for redistribution (Alesina and Giuliano, 2011) to illustrate the channels through which equity in the tax and transfer system is likely to influence people’s tax morale. The most immediate theoretical implication from these findings is that research on tax compliance needs to engage further with how the progressivity of taxes impacts people’s utility. To put this formally using the utility function in Section 2, the weighting ($\gamma_{i}^{t}$) people place on the difference between their perceived and preferred levels of progressivity in the tax and transfer system ($Q_{bi}^{t}-Q_{i}^{t*}$) is non-trivial. As such, the role of equity in the tax system should be considered alongside more commonly cited motivations for why people pay (or do not pay) tax, such as to keep up with social norms (Hallsworth, 2014), to contribute to the provision of public goods (Giaccobasso et al., 2022), and because they have a positive outlook on the government (Cullen et al., 2021). While there has been some related work along these lines in the United States (Stantcheva, 2021), this study builds on these foundations to illustrate how progressivity in the tax and transfer system in general impacts people’s tax morale, as well as by showing how generalizable these trends are across a diverse set of developing countries.

The order of magnitude of the impact of the taxes treatment on tax morale was in line with seminal cross-country randomized survey experiments (Alesina et al., 2018, Alesina et al., 2022) and if this translated into actual tax compliance behavior the effects would be non-trivial (e.g., they would be of a similar size to recent work such as Balan et al., 2022). Given the novelty of this study, it is challenging to precisely compare the order of magnitude of the treatment effects to related work. However, there is a further limitation regarding the nature of the treatment. Ultimately, the information provided to respondents in the randomized survey experiment is largely binary (taxes and/or transfers are either progressive or not). As a result, this means it is not possible to estimate how the order of magnitude of progressivity in the tax and transfer system matters (technically the figures in the treatments provide this information, but it is unlikely to have been fully comprehended by some respondents). The similarity in the impact of the taxes treatment within the two groups of countries (with taxes being either progressive or not) would suggest that the order of magnitude of progressivity was not necessarily a particularly important consideration for respondents. Rather, it appears that what influenced respondents was purely whether or not the tax system was progressive (i.e., it was a binary consideration).

### 5.3 Implications for policy makers

A key implication for policymakers from this study is that de facto changes to the degree of equity in the tax system will impact people’s tax morale. As discussed in the introduction, changes to tax policy and/or administration that intend to improve a country’s fiscal position are likely to change the degree of progressivity in the tax system and it is necessary for policymakers to better understand people’s responses. Changes that improve progressivity could have an additional benefit by increasing people’s tax morale. The opposite could also be the case, whereby changes that reduce progressivity could, in turn, decrease people’s tax morale. In the most extreme case, it is possible that changes that reduce progressivity, which were intended to improve the fiscal position of a country, could undermine tax compliance to a point whereby the net impact on revenue is negative. Ultimately, the exact order of magnitude of these “second round” effects that have an *additional benefit* (*backfire effect*) from increasing (decreasing) progressivity in the tax system will likely vary over time and across countries. However, the results of this study do suggest that policymakers should take these “second round” effects seriously.

Policymakers can also learn from this research about the benefits of communicating effectively with the general population about the purposes of tax reforms, especially when they are implemented in tandem with changes to the government transfer system. Clearly, the results show that most people have a preference for progressive taxes and this can be utilized by policymakers to justify changes to the tax system. Alongside other reasons for tax reform (e.g., improving a country’s fiscal position), communicating the role of taxes in promoting greater equality (when this is the case) appears to be an important tool in policymakers’ arsenal, particularly in democratic regimes. Even in the absence of a reform agenda, communicating to taxpayers about the progressive aspects of the tax system in their country would appear to be a way to boost compliance. Further, there appears to be ample scope for information campaigns to be done by policymakers to help the general population understand how taxes help fund the government transfers that benefit so many households.

A potential reason why this approach may have been under-exploited by policymakers is they are most interested in richer individuals paying tax as, ultimately, this will collect the most revenue, and they are concerned that these taxpayers may be the least receptive to messages about progressivity. Our study presents mixed results on this point. While there was some variation between respondents across the perceived income distribution, these differences were not statistically significant. Further, there was evidence to suggest that the negative overall effect of the taxes and combined treatments on respondents’ tax morale was larger among segments of the population who face a sizable tax liability and have greater scope to avoid paying tax (although once again differences were not statistically significant). Collectively, these results do not suggest that concerns regarding upsetting richer taxpayers warrant discarding communication campaigns about progressive reforms to taxation. In fact, the descriptive finding that there is widespread support for progressive taxes and transfers, even among richer individuals, would suggest progressive reforms to tax and transfer systems in most developing countries may be far more popular than what many policymakers appreciate.

### 5.4 Directions for future research

A key area for future research that the findings from this study would suggest is worth pursuing is testing how equity in tax and transfer systems influences taxpayer behavior using administrative data (ideally across countries). Randomized survey experiments, including seminal studies by Kuziemko et al. (2015), Alesina et al., 2018, Alesina et al., 2022 and Stantcheva (2021) rely on the use of self-reported outcomes. While this is incredibly useful, a natural next step is to try to link these outcomes to administrative data. However, in this case, survey measures of tax morale are a plausible, but far from perfect, proxy for compliance (e.g., see discussion in Besley et al., 2021 and Luttmer and Singhal, 2014). Another area worthy of greater attention is exploring the extent to which other aspects of tax morale exist in developing countries. For example, a randomized survey experiment examining issues to do with fairness in the tax system would make a large contribution to the literature and may matter more to taxpayers than equity in the tax system.

## Data Availability

Data will be made available on request.

## Appendix A Supplementary data

MMC S1 This includes further information about the methodology and additional results.

## Footnotes

1 Understanding tax morale is particularly important in developing countries as there is typically a weaker capacity of revenue authorities to enforce tax legislation (Prichard et al., 2019, Prichard et al., 2022).

2 In general, tax and transfer systems are less progressive in countries that rely more heavily on indirect taxes (e.g., value added tax) compared to direct taxes (e.g., personal income tax) and/or indirect transfers (e.g., subsidies) compared to direct transfers (e.g., targeted cash transfers).

3 The CEQ approach was developed by the Commitment to Equity Institute (CEQ Institute) at Tulane University. The methodology, implementation guidelines, applications, and software of the CEQ approach can be found in Lustig (2018).

4 For example, respondents were asked on a Likert scale about whether it is important for people to pay tax, if the government always has a right to make people pay tax, and if they would still pay tax in the absence of enforcement.

5 Respondents in the tax treatment group were informed about the actual level of tax progressivity in their country. This means that I cannot test the effect of providing positive and negative information about tax progressivity in the same country. As such, it is not possible to rigorously conclude that it is more costly in tax morale terms to reduce progressivity than to increase it. However, the results do provide some basis to believe that tax morale can be more “easily unraveled” due to reductions in equity than built up due to increases in equity.

6 In some respects, this work involved applying (Becker, 1968) seminal work on crime and punishment to tax compliance.

7 In general, tax and transfer systems are less progressive in countries that rely more heavily on indirect taxes (e.g., value-added tax) compared to direct taxes (e.g., personal income tax) and/or indirect transfers (e.g., subsidies) compared to direct transfers (e.g., targeted cash transfers). In each of these countries, almost all households indirectly pay tax (e.g., through paying value-added tax) and/or indirectly receive government transfers (e.g., through receiving fuel subsidies) (Commitment to Equity (CEQ) Institute, 2021).

8 This survey instrument was translated into the following languages: Spanish (for use in Mexico and Colombia), Arabic (for use in Jordan), Bahasa (for use in Indonesia), Swahili (for use in Tanzania) and Sinhala and Tamil (for use in Sri Lanka).

9 More complicated questions (including list experiments that “implicitly” capture tax morale) were considered but ultimately discarded as the piloting process illustrated that keeping questions simple was by far the best way to maximize data quality and minimize attrition (see Appendix A for details).

10 In line with the IRB approval, the information provided to respondents had to be truthful. This means that it was not possible to test the impact of both positive and negative information treatments in the same country as only one of these could be true.

11 For example, if both aspects were included it is possible some respondents may react to the degree of progressivity, while others may react to the level of taxes and transfers, and it would not be possible to differentiate between these.

12 The exception is the richest quintile in Mexico.

13 It is important to note that the income distribution is constructed based on where people perceive their household to be as opposed to being determined based on reported household income. Consistent with the findings in Hoy and Mager (2021a), this resulted in most respondents perceiving themselves to be in the middle quintiles and only a very small share stating that they were in the poorest or richest quintile in each country (see Table A2 in Appendix B).

14 It is not possible to rigorously conclude that it is more costly in tax morale terms to reduce progressivity than to increase it. However, the results do provide some basis to believe that tax morale can be more “easily unraveled” due to reductions in equity than built up due to increases in equity.

15 Three reasons were noted in the pre-analysis plan. Firstly, on average, the share of household income collected in taxes is much higher than what is provided in transfers, which means people may be more concerned about how taxes are distributed compared to transfers. Secondly, there is reason to believe that “loss aversion” could exist where people’s utility is more likely to be influenced by “losing” from paying tax than by “gaining” from receiving a transfer. Thirdly, people’s awareness of when they pay tax may be higher than their awareness about when they receive a transfer.
