# Supplementary material for: How does progressivity impact tax morale? Experimental evidence across developing countries
Source: J Dev Econ. 2025 Jan;172:103398. doi: 10.1016/j.jdeveco.2024.103398 (PMC11625699; doi:10.1016/j.jdeveco.2024.103398)
Supplement: MMC S1 — This includes further information about the methodology and additional results. [file mmc1.pdf]

# List of Online Appendices

**Appendix A** - Further methodological details and results

**Appendix B** - Additional tables and figures

**Appendix C** - Survey instrument (English version)

**Appendix D** - Country specific treatments (English version)

# Appendix A – Further methodological details and results

## Approach to data collection

Ideally, face-to-face surveys collecting a representative sample of the general population using a sampling frame, such as a recent census, would have been conducted in each of the countries in this study. Not only are the costs involved in doing this prohibitive, but there are also issues with conducting face-to-face surveys during a pandemic. While phone surveys present a popular alternative, this is not an appropriate format for a survey along these lines. The treatments are designed to be visual in nature and it is not possible to communicate these messages fully via a phone call. This left an online survey as the most promising option for data collection, even though there are challenges with representativeness that need to be recognized and can be overcome to some extent.

A major challenge with conducting an online randomized survey experiment in low- and middle-income countries is collecting a representative sample of the total population. Unlike high-income countries where internet access is nearly universal, the share of the total population with internet access in the countries in this study varies from 20 to 67 percent. Furthermore, there is limited existing online survey "infrastructure", such as what exists in many high-income countries where market research firms run online opinion polls daily from a large pool of pre-registered respondents who regularly complete surveys. This is far less common in low- and middle-income countries and there are reasons to be concerned about just how nar-

row a subset of the population would participate in an engagement like this. Similar concerns exist regarding the use of online labor platforms, such as MTurk, in a low- and middle-income country context.

Alternative approaches to online data collection in low- and middle-income countries can crudely be categorized as providing "opt-in" or "opt-out" options. An example of the former would be to use social media advertisements to invite people to participate in an online survey. While this "opt-in" approach may be attractive as it is easy to implement, I identified at least two shortcomings that I felt meant this approach was not ideal for this study. Firstly, there is a clear concern regarding selection bias as people who would "opt in" to a survey based on a social media advertisement potentially have some unobservable characteristics that make them distinct from the rest of the population. It is challenging to estimate the extent to which these unobservable characteristics exist without gaining access to administrative data from social media providers. Secondly, as I was asking about a sensitive topic (tax compliance) it is possible that participants would not provide honest answers as they could easily be identified through the platform that they were opting into the survey on (e.g., Facebook). As such, on balance, I felt that an "opt-in" approach along these lines would not be ideal for this study.

Despite these concerns regarding "opt-in" online data collection via social media, I still attempted to pilot the randomized survey experiment via Facebook and Instagram in the countries with the two smallest "internet" populations in this study (Tanzania and Jordan). These countries were chosen as reaching a large enough sample size to have statistical power to detect effects from the treatments would be

the most challenging in these settings. The survey was non-incentivized (to minimize concerns about experimenter demand effects and to ensure respondents did not need to provide identifiable information), and to comply with research ethics protocols, the social media advertisements stated that respondents would be asked questions about taxes. Partly because of these constraints it was not possible to solicit even half of the total respondents required for the survey via this sampling method in Tanzania and Jordan, despite the social media advertisements reaching millions of unique social media users over a period of two months. These challenges that were faced when trying to pilot an "opt-in" approach to the survey provided further rationale behind using an alternative approach for this study.

Data was collected for the online randomized survey experiment in this paper using an "opt-out" approach offered by the survey firm, RIWI. They capture a sample of respondents that is broadly representative of the internet population in each country by using Random Domain Intercept Technology. This involves sampling internet users who incidentally access expired or inactive domains (i.e., which often result in a "404 error"). As domain names regularly change and they often do not automatically redirect internet users, it is commonplace for the internet-using population to incidentally access inactive domains. Research suggests the likelihood of accessing an inactive domain is approximately proportional to having access to the internet (IRIS, 2021). RIWI exploits this by redirecting users from inactive domains to a website inviting them to take part in a survey. At this point, people can decide whether to continue to participate in the survey or "opt out". RIWI tracks information about the device used and the operating system used by people who are redirected to the

survey platform, even if they do not answer a single question. In addition, the first question people are asked is about their age and sex. As a result, I observe how "opt-out" rates from a representative sample of the internet population vary based on the characteristics of respondents (for example, I am able to measure whether people using smartphones disproportionally opt-out of the survey). A shortcoming of this "opt-out" approach is that high rates of attrition occur early in the survey. However, given that I track how attrition varies by the characteristics of respondents and the survey experiment is at the back end of the survey, this does not undermine the integrity of the study.

I also examine whether particular types of internet users were more likely to participate in and complete the survey. In general, respondents using a smartphone were less likely to begin the survey, and conditional on starting the survey, they were slightly less likely to complete all the questions (see Table A4 in Appendix B). This is to be expected as the visual components of the survey are easier to see on a larger screen. To address concerns about this, an additional robustness check was conducted using "device type weights". This involved adjusting the results to ensure that in each country, the share of respondents using a smartphone at the end of the survey was the same as the share of respondents using a smartphone that were exposed to the survey (i.e., they saw the invitation to participate in the survey). I show that using these "device type weights" had no meaningful impact on the results (see Table A5 in Appendix B).

## Additional methodological details

### Selection of countries

The eight countries (Colombia, Ghana, Indonesia, Jordan, Mexico, Sri Lanka, South Africa, and Tanzania) focused on in this study were selected for the following reasons. Firstly, there is very limited, standardized, cross-country data available about the progressivity of taxes and government transfers in developing countries. By far, the largest effort that has been made to collect and disseminate this information has been through the Commitment to Equity Institute at Tulane University, which is headed by Nora Lustig (CEQ, 2021). Importantly, the CEQ database takes into account the actual behavior of households and consequently presents the de facto distribution of taxes and transfers (i.e., factoring in tax compliance and the accuracy of targeting of transfers). Estimates have been produced of the difference between the gross and net GINI index in over 55 developing countries through this work program in partnership with the World Bank (see Figure A1). These estimates are based on standardized household income and expenditure surveys, and in 2020, a cross-country database that provided dis-aggregated information in a standardized way for many countries was publicly released through a joint initiative between universities, civil society, and international organizations (Lustig, Mariotti, and Sánchez-Páramo, 2020). However, due to a range of factors, including governments' reluctance to make certain information publicly available, information about the progressivity of direct and indirect taxes, as well as direct and indirect government transfers (including subsidies), is restricted to a far smaller subset of these countries. This subset of countries was the starting point for selecting which countries to include in this study.

Secondly, the time and costs involved in collecting data online in low- and middle-income countries are considerably lower when there is a high internet population in absolute terms. As such, countries with high populations and/or high internet penetration rates were focused on as part of this study. For example, some countries in this study, like Tanzania have relatively high total populations (60 million), but low internet penetration rates (20 percent), whereas other countries, like Jordan have a relatively low total population (10 million) but relatively high internet penetration rates (67 percent) (World Bank, 2021).

Thirdly, due to funding reasons, it was necessary to collect a diverse set of countries in each of the major regions with low- and middle-income countries (i.e., Latin America, West Africa, East Africa, the Middle East, South Asia, and East Asia) as well as across various income levels. This restricted the choice set considerably in some regions; for example, Indonesia was the only country in East Asia with publicly available data about the distribution of direct and indirect taxes, as well as direct and indirect government transfers (including subsidies) (CEQ, 2021).

Finally, efforts were made to ensure that the information about the distribution of taxes and government transfers in the database was still likely to provide a reasonable estimate of what would exist in 2022. However, in some countries, such as the Islamic Republic of Iran, there have been significant changes to the tax and transfer system since the survey included in the database took place. As a result, it would not be a realistic approximation of how taxes and government transfers were likely to be distributed in 2022.

## Details about Survey Design

Efforts were also made to ensure that respondents were likely to trust the content of the treatments without inducing experimenter demand effects by following a similar approach to seminal work by Alesina et al. (2018). For example, respondents were informed that the information they were provided with recently became publicly available online through a collaboration between universities, civil society and international organizations (see Lustig, Mariotti and Sánchez-Páramo, 2020). In addition, given the extensive analysis in prior work to illustrate that experimental demand effects are unlikely to be present in these types of randomized survey experiments (e.g., see Kuziemko et al., 2015 on a related topic), it is very unlikely to be an issue in this study. Especially given that experimenter demand effects are much less likely to be a concern in experiments like this study where respondents are anonymous, they are not incentivized, the survey is online, there is no information provided about the research hypotheses, the treatments are framed in a "neutral" way, and the analysis is between-subjects (Haaland et al., 2023; de Quidt, Haushofer and Roth, 2018). Furthermore, all respondents were asked about their prior beliefs about the progressivity of taxes and transfers immediately before the treatment, which means the purpose of the treatment is somewhat naturally concealed by potentially being viewed as feedback on whether the respondents' beliefs were accurate (Haaland et al., 2023).

### Questions measuring people's prior beliefs and preferences

Before the treatments, respondents were asked to provide information about their beliefs about their household's position in the income distribution in their country, their beliefs and preferences for the distribution of taxes and government transfers in their country, preferences for the level of inequality in their country, and whether they viewed their households as being net contributors to or beneficiaries from the tax and transfer system. These questions were either sourced from existing studies in a series of developing countries (Q4 and Q5) (e.g., see Hoy and Mager, 2021a) or were specifically developed for this study (Q6–Q11). The questions developed for this study were based on the structure of standardized questions in the literature (e.g., they use a Likert scale), informed by expert feedback, and modified based on the piloting process to ensure these new questions were adequately comprehended by respondents (see Appendix A for details of the piloting process). The final set of questions used in this study were intentionally simple and qualitative as more complex and quantitative questions resulted in considerable measurement error during the piloting process, indicating a lack of understanding by respondents. This is partly a consequence of the setting of the survey experiment, whereby many respondents had very low levels of education.

### Questions measuring people's tax morale

The first question to measure tax morale (Q14) directly asks respondents whether they would pay tax if they knew that they would not be caught for non-compliance. A potential shortcoming of a direct measure of tax morale is that people may be

very unwilling to provide honest answers, and consequently, people's answers to this question are likely to be particularly inelastic to an information treatment. In addition, the share of respondents claiming they have high tax morale in the control group (i.e., in the absence of additional information provided through the treatments) will almost certainly be higher than what is the case. Therefore, this question is likely to suffer from "ceiling effects" (Po, 1998), which means it provides a lower-bound estimate of the impact of the treatments.

The remaining four "indirect" questions have been sourced from existing studies on this topic in low- and middle-income countries and capture slightly different aspects of tax morale. The second question (Q15) measures the degree to which respondents believe people not paying tax is understandable. It was used in the Afrobarometer (2012; 2013; 2015) as well as by Ali, Fjeldstad and Sjursen (2014). The third question (Q16) measures whether people believe paying taxes is important and was used by Khwaja et al. (2020). The fourth question (Q17) measures people's unconditional beliefs about the extent to which the government has the right to make people pay taxes, and it has been included in many rounds of the Afrobarometer (2002; 2003; 2004; 2008; 2012; 2013; 2014; 2015; 2017). The fifth question measures the degree to which people believe that paying tax should be conditional on what the government spends tax on and is a slightly modified version of what was used by Prichard, Jibao, and Orgeira (forthcoming).

## Pilot data

The proposed survey instrument went through an extensive review process within the World Bank prior to being piloted in December 2021. The internal review process identified ways in which the survey instrument could reflect best practices in the literature (e.g., avoiding ceiling effects on the outcome variables by phrasing questions to ensure greater variation of responses across a Likert scale). Reviewers also emphasized that during the piloting process it will be crucial to examine whether respondents adequately comprehend the treatments and the questions. As such the primary focus of the piloting that took place was to ensure the responses that were gathered indicated the respondents understood the survey instrument. In addition, piloting provided an opportunity to verify the assumptions made about the size of the treatment effects in the statistical power calculations and to identify ways in which the experiment could be designed in a manner to minimize attrition. These three issues are discussed one by one below following a description of the piloting process.

*Implementation of the piloting process*

The survey instrument and experiment were piloted with 1,061 respondents (who completed the survey), which made up a representative sample of the internet population in India in December 2021. India was selected as an appropriate location to pilot the survey as this is where the survey firm typically conducts pilots (due to the diverse, but very large, population where English is commonly used on the internet); it has a similar level of development to many of the countries in the full study and as I was not including India in the full study, I did not need to be concerned about

contaminating the pool of respondents.

There were two phases to the pilot. The first phase involved using visual stimuli for some of the questions (somewhat similar to what Hoy and Mager (2021b) used in high-income countries) capturing people's prior beliefs and preferences about the distribution of taxes and transfers as well as levels of inequality in their country. In this version of the survey instrument that had been approved through the internal review process at the World Bank, respondents were required to select the distribution of taxes and transfers that exist in their country based on actual examples. Specifically, the options provided for respondents to select from were based on the actual progressivity of taxes in Tanzania in 2011, Colombia in 2014, and Jordan in 2017. In addition, respondents were randomly allocated to receive questions from a pool of seven potential questions about their willingness to pay tax. This process helped to inform which five questions should be included in the full study. In total, 511 respondents completed this phase of the pilot.

The main change in the second phase of the pilot was replacing the questions from the first phase that involved visual stimuli with basic questions that aimed to capture people's prior beliefs and preferences about the distribution of taxes and transfers as well as levels of inequality in their country on a Likert scale. This approach brought the format of these questions into line with the rest of the survey. A shortcoming of this approach was that it was no longer possible to identify whether people's beliefs and preferences matched examples of the actual level of progressivity of taxes in some low- and middle-income countries. In the second phase of the pilot, respondents continued to be randomly allocated to receive a subset of questions about

their willingness to pay tax. In total, 550 respondents completed this phase of the pilot.

*Lessons learned through the piloting process*

There were three key lessons that emerged from the two phases of the pilot that informed the final survey instrument. Firstly, there was a clear need to keep the survey instrument as simple as possible. Answers to the questions that included visual stimuli in the first phase of the pilot suggested respondents did not adequately comprehend the options they were presented with. Responses were very evenly distributed across the options in each of the four questions about people's beliefs and preferences in regard to the distribution of taxes and transfers in their country. To test whether this was primarily due to measurement error, in the second phase of the pilot, respondents were randomly allocated to either the question format from phase one or basic questions about their views on how taxes and transfers are distributed using a Likert scale. The results were substantially different between these approaches with the basic question format returning results far more consistent with previous literature. Specifically, the results showed most people tend to prefer richer households to pay more taxes than poorer households and poorer households to receive more government transfers than richer households (i.e., most people tend to prefer progressivity in the tax and transfer system). As such, I decided that the final survey instrument should rely on these basic questions to capture people's prior beliefs and preferences, even though this means that the options provided are not based on actual progressivity of taxes and transfers in countries. I believe that capturing higher quality, reliable responses is of greater importance.

Secondly, the results of the piloting process provided me with confidence that the sample size in the final study would be adequate. The point estimates of the treatment effects were promising as they indicated variation between respondents in the treatment and control groups of an order of magnitude that I would be powered to detect at standard levels (i.e., an alpha of 0.05 and beta of 0.2) when the full sample of respondents is reached (i.e., 3,600 as opposed to 1,061). The direction of the treatment effects was also often in line with the primary hypotheses of this study.

Thirdly, the piloting process highlighted ways to minimize attrition during the survey experiment and the most straightforward way was by removing list experiments from the study. Specifically, there was low attrition for the outcome variable questions included in the final survey instrument, whereas around one-quarter of respondents dropped out during the two list experiments that were included in the pilot. Removing the list experiments from the randomized survey experiment was not a major issue for our study as there is debate in the literature about the value of this approach in general and I would have potentially faced considerable issues with inadequate statistical power. In the second phase of the pilot I also randomized alternative data quality check questions between respondents immediately prior to the survey experiment and found that our original question from the first phase of the pilot outperformed an alternative question that was used by Alesina et al. (2018). As such, I felt confident that including a question that asks respondents to drop out prior to the treatment if they are unwilling to complete the survey experiment would serve as an effective way to minimize attrition post-treatment. I was also reassured by the lack of differential attrition observed throughout the piloting process.

## Coding of variables

Q0 – Age - age1834= 1 if respondent aged 18–34 years (respondents under 18 automatically discarded), 0 if respondent aged 35 years or older

Q0 – Sex - male = 1 if respondent male, 0 otherwise

Q1 – Education - edusecorless = 1 if respondent selected primary or secondary education, 0 otherwise

Q2 – Location - largecity = 1 if respondent selected large city or suburb, 0 otherwise

Q3 – Employment type - working= 1 if respondent selected employee or self-employed/small business owner, 0 otherwise

Q4 – Prefer lower inequality - lowerineq = 1 if respondent selects strongly agree or agree, 0 otherwise

Q5 – Perceived position in national income distribution - pB40 = 1 if respondent selected poorest or second poorest quintile, 0 otherwise

Q6 – Household paid large share of income in tax - largetax = 1 if respondent selects strongly agree or agree, 0 otherwise

Q7 – Household paid more in tax than received in transfers - netcont = 1 if respondent selects strongly agree or agree, 0 otherwise

Q8 – Perceived taxes as currently progressive - curprogtax = 1 if respondent selects strongly agree or agree, 0 otherwise

Q9 – Prefer taxes to be progressive - progtax = 1 if respondent selects strongly agree or agree, 0 otherwise

Q10 – Perceived transfers as currently progressive - curprogtrans = 1 if respondent selects strongly agree or agree, 0 otherwise

Q11 – Prefer transfers to be progressive - progtrans = 1 if respondent selects strongly agree or agree, 0 otherwise

Q12 – Data quality check - willcomplete = 1 if respondent selected yes, 0 otherwise

TREATMENT PROVIDED

Q14 – Will not pay without enforcement - willpaytax = 0 if respondent selects strongly agree or agree, 1 otherwise

Q15 – Not paying tax is wrong and punishable - wrongpunish = 1 if respondent selected wrong and punishable, 0 otherwise

Q16 – Paying taxes is important - importanttopay = 1 if respondent selects strongly agree or agree, 0 otherwise

Q17 – Government has right to pay tax - righttotax = 1 if respondent selects strongly agree or agree, 0 otherwise

Q18 – Do not Refuse - donotrefusepaytax = 1 if respondent selects strongly disagree or disagree, 0 otherwise

# Additional results

## Descriptive findings

### Tax morale across countries

People's tax morale varied between the different questions asked and across countries (these findings are based on respondents in the control group). Figure A4 shows that depending on the specific question and the country, between 19 and 89 percent of respondents selected an answer that was consistent with being willing to pay tax. In five of the eight countries in this study (Colombia, Indonesia, Mexico, Sri Lanka, and South Africa), there was broadly similar tax morale as there was only a 6 to 16 percentage point difference across countries for a given question. On average, people's tax morale was more than 10 percentage points higher in Ghana and Tanzania than in the other countries, while the opposite was the case in Jordan. The findings for each question are broadly consistent with the general patterns in the surveys that the questions were sourced from. For example, across multiple rounds of the Afrobarometer (2012, 2013, 2015) between 45 and 63 percent of survey respondents in Tanzania stated that not paying tax was wrong and punishable, while 47 percent of people in Tanzania in this survey agreed with this statement.

**[Figure A4]**

The characteristics associated with being willing to pay tax varied considerably across the questions that were asked. Multivariate regression analysis shows that

the most common pattern across questions is that those aged between 18 and 34 years old were less likely to state they would be willing to pay tax compared to those aged 35 years and older (see Table A9 in Appendix B). In addition, respondents who perceived themselves to be in the middle of the income distribution were more likely to state that they were willing to pay tax and, interestingly, people who thought they were in the richest quintile were often the least likely to state they would be willing to pay tax (although differences were typically not statistically significant). No other background characteristics were consistently associated with answers to the various questions that were used to measure tax morale across countries.

These descriptive survey findings about tax morale across countries cannot be directly compared to actual taxpayer behavior as this information is not available in a standardized way (PWC, 2022; USAID, 2019; World Bank, 2022b). However, as discussed above, these survey measures of tax morale provide a plausible but far from perfect proxy for tax compliance (Luttmer and Singhal, 2014). I also examine how these measures of tax morale compare to other cross-country measures, examining somewhat related topics of corruption (Transparency International, 2023), rule of law (World Justice Project, 2023), and beliefs about the rich being selfish (Almas et al. 2022). I observe no clear correlation between these concepts and tax morale (see Appendix Table A10), which suggests that these questions about tax morale are capturing something beyond what is measured in existing cross-country measures.

## Extensions and robustness checks

### Heterogeneous treatment effects among segments of the population that face different levels of tax liabilities

While the main results of the taxes and combined treatments suggest that, on average, people's tax morale is influenced by whether the tax system is progressive in their country, from a policymaker's perspective, it is critical to understand how different segments of the population respond to the treatments based on their tax liabilities. Particularly, if the main effects were purely driven by people who don't face substantial tax liabilities or the ability to avoid paying tax, then the findings may be less relevant from a revenue perspective. In line with the "secondary hypotheses" in the pre-analysis plan, I also examine heterogeneous treatment effects based on respondents' perceived place in the income distribution, respondents' employment type, respondents' beliefs about whether their household pays a large share of their income in tax and respondents' beliefs about whether their household pays more in tax than they receive in transfers. Given the limitations of asking respondents about their perceived place in the income distribution, I complement this by examining an objective measure that provides a straightforward indication of household welfare. Specifically, I group respondents into three categories (low, medium, high) of socio-economic status (SES) based on the education level of respondents and their geographic location. Respondents with tertiary education that live in urban areas are deemed to have high SES, respondents in rural areas with only secondary education or less are deemed to have low SES, and the remainder of respondents are

categorized as having medium SES.[1]. Heterogeneous treatment effects on each of these dimensions provide insights into how segments of the population with different tax liabilities respond to the treatments. To maximize statistical power and to streamline the discussion in the body of the paper, the taxes and combined treatment groups are merged and compared to the control group.[2] For completeness, I present the heterogeneous effects for each treatment in Tables A15-A19 in the Appendix.

I do not find compelling evidence to suggest that there are large differences in the impact of the taxes and combined treatments across segments of the population that face different levels of tax liabilities. Figure A5 presents the impact of the merged treatment on the tax morale index for each of the dimensions discussed above (respondents' perceived place in the income distribution, respondents' socio-economic status, respondents' employment type, respondents' beliefs about whether their household pays a large share of their income in tax and respondents' beliefs about whether their household pays more in tax than they receive in transfers). Figure A5a shows that in countries where the tax system was progressive, there are somewhat consistent findings across the poorest four quintiles of the perceived income distribution, but there was some evidence of an opposing effect for the richest quintile (although differences are not statistically significant). In countries where taxes were not progressive, the treatment effect on the WTP tax index for the poorest three quintiles was somewhat similar and close to zero for the richest two quintiles. However, as noted in the descriptive results, most respondents perceive themselves to be in the middle quintiles,

[1]These respondents either have tertiary education and live in rural areas or less than tertiary education and live in urban areas

[2]In this setup, the merged treatment can be thought of as providing some form of information about whether the tax system is progressive or not.

which means these findings across the perceived income distribution should be interpreted with caution, especially given the absence of statistically significant effects.

**[Figure A5]**

The other heterogeneous treatment effects displayed in Figure A5 suggest that the negative overall effect of the taxes and combined treatments on respondents' tax morale was larger among segments of the population who face a sizable tax liability and have greater scope to avoid paying tax (i.e., the self-employed). Figure A5b shows that respondents in the treatment group in countries who had medium or high socio-economic status were the most likely to report lower levels of tax morale in countries where the tax system was not progressive (there were no differences in the treatment effect between respondents by socio-economic status in countries where the tax system was progressive). Figure A5c shows that respondents in the treatment group who were not working were the most likely to increase their tax morale in countries where the tax system was progressive, whereas self-employed respondents in the treatment group were the most likely to decrease their tax morale in countries where the tax system was not progressive. Figure A5d shows that respondents in the treatment group whose household did not pay a large share of their income in tax were the most likely to increase their tax morale in countries where the tax system was progressive, whereas respondents in the treatment group whose household did pay a large share of their income in tax were the most likely to decrease their tax morale in countries where the tax system was not progressive. Figure A5e shows

that respondents in the treatment group whose household was a net beneficiary were the most likely to increase their tax morale in countries where the tax system was progressive, whereas there were no differences on this dimension in countries where the tax system was not progressive.

**Differences between the treatments**

The main results of this study appear to be driven by the content of the treatments, as opposed to simply receiving a treatment. The experiment was designed in a way that allowed for comparisons to be made across treatments to rule out concerns that the overall effects were purely due to receiving any information about taxes and transfers. This was possible in six of the eight countries (Ghana, Indonesia, Jordan, Sri Lanka, South Africa and Tanzania) where the direction of the taxes and transfers treatments were opposing one another (e.g., in Ghana the taxes treatment was highlighting that the system was progressive whereas the transfers treatment was stating the opposite) (see Table A20 in Appendix B). There was a large statistically significant difference between respondents' tax morale, depending on whether the treatment they received indicated that the tax and transfer system was progressive or not progressive.

**Representativeness of the survey**

The main results of the randomized survey experiment hold with and without weights applied to adjust the data to match the general population and with and without weights applied to adjust the data to match the characteristics of the internet pop-

ulation that was invited to participate in the survey. Firstly, as described in the methodology (Section 3), the results presented throughout the body of the paper have weights for age and sex to adjust the data to match the general population. In Appendix B, the results are also presented without these weights, and the findings are very similar (see Table A3). Secondly, the characteristics of the population that were invited to participate in the survey were compared to those that completed the survey to examine whether differences existed. The main dimension that was identified was whether people were participating in the survey via a smartphone. Those who were tended to be less likely to participate in the survey in the first place and less likely to complete the survey conditional on starting (see Table A4 in Appendix B). To examine whether this was driving the results, I re-weighted the data to match the original composition of respondents (smartphone vs other device types) that were invited to participate in the survey, and this did not have a noteworthy impact on the results (see Table A5 in Appendix B).

**Robustness checks**

The main results of the randomized survey experiment did not vary considerably when conducting a series of robustness checks. These checks involved removing respondents who took too long or short a period of time to complete the survey as well as conducting the analysis using alternative econometric specifications (see Tables A21-A22 in Appendix B). In addition, I show that the results are unlikely to be due to differential attrition between the treatment and control groups by using Lee (2009) bounds analysis (see Table A23 in Appendix B).

**Machine learning algorithm of heterogeneous treatment effects**

I conducted an exploratory analysis to provide a direct comparison of the relative order of magnitude of the heterogeneous treatment effects. I use a machine-learning algorithm (Generalized Random Forest) to identify where the greatest heterogeneity occurs across people's beliefs about and preferences for the progressivity of taxes as well as the demographic characteristics captured in the survey. This approach involves calculating the degree of heterogeneous treatment effects for each dimension, and the relative degree of variation is placed on a scale from 0 to 1 so that the total across all dimensions is equal to 1. This provides a simple way to directly compare the relative size of heterogeneous treatment effects across dimensions. The results of this analysis clearly illustrate that there are far greater heterogeneous treatment effects based on respondents' beliefs and/or preferences than other factors (see Table A24 in Appendix B). In general, heterogeneity based on these dimensions is around twice as large as other factors. In countries where the tax system was progressive, people's preference for progressivity was the dimension with the largest variation in heterogeneous effects from the taxes treatment, which is consistent with what can be seen in Panel A in Table 2. In countries where the tax system was not progressive, people's beliefs about progressivity was the dimension with the largest variation in heterogeneous effects from the taxes treatment, which is consistent with what can be seen in Panel B in Table 2.

## Appendix B - Additional Tables

Table A1: Age and sex of survey respondents and the general adult population

| | Male (%) survey | 18-34 years (%) survey | Male (%) population | 18-34 years (%) population |
|---|---|---|---|---|
| Colombia | 59.7 | 56.2 | 49.1 | 43.8 |
| Ghana | 78.7 | 78.1 | 50.7 | 55.6 |
| Indonesia | 67.2 | 73.3 | 50.4 | 43.4 |
| Jordan | 57.2 | 70.2 | 50.6 | 53.3 |
| Mexico | 62.5 | 53.5 | 48.9 | 45.1 |
| South Africa | 58.7 | 60.9 | 49.3 | 49.1 |
| Sri Lanka | 76.9 | 62.3 | 48.0 | 36.6 |
| Tanzania | 70.8 | 79.4 | 50.0 | 60.0 |

Note: This table shows the age and sex of survey respondents compared to the general adult population in each country.

*Source: World Bank, 2021*

## Table A2: Share of survey respondents with specific demographic characteristics

| | Sec edu or less | Large city | Working | Believe B40 | Believe T40 |
|---|---|---|---|---|---|
| Colombia | 0.469 | 0.696 | 0.576 | 0.396 | 0.0514 |
| Ghana | 0.420 | 0.576 | 0.499 | 0.269 | 0.133 |
| Indonesia | 0.250 | 0.424 | 0.604 | 0.244 | 0.0531 |
| Jordan | 0.442 | 0.653 | 0.416 | 0.439 | 0.0870 |
| Mexico | 0.272 | 0.673 | 0.658 | 0.236 | 0.0678 |
| South Africa | 0.500 | 0.287 | 0.458 | 0.404 | 0.0483 |
| Sri Lanka | 0.602 | 0.342 | 0.608 | 0.335 | 0.0724 |
| Tanzania | 0.457 | 0.519 | 0.538 | 0.247 | 0.0699 |

Note: This table presents demographic characteristics of survey respondents. *Sec edu or less:* Based on Q1, which asks whether respondents their level of education (variable takes value of 1 if they select "Primary or less" or "Secondary" and 0 otherwise). *Large city:* Based on Q2, which asks respondents about where they live (variable takes value of 1 if they select "Large city" and 0 otherwise). *Working:* Based on Q3, which asks whether respondents their current employment status (variable takes value of 1 if they select "Employee" or "Self employed" and 0 otherwise). *Believe B40:* Based on Q5, which asks respondents about their households place in the national income distribution (variable takes value of 1 if they select "Poorest group" or "Second poorest group" and 0 otherwise). *Believe T40:* Based on Q5, which asks respondents about their households place in the national income distribution (variable takes value of 1 if they select "Richest group" or "Second richest group" and 0 otherwise).

Table A3: Overall effects of the treatments (without weights)

| | Direct b/se/p | Punishable b/se/p | Important b/se/p | Right to Tax b/se/p | Do not Refuse b/se/p | INDEX b/se/p |
|---|---|---|---|---|---|---|
| Taxes (Progressive) | 0.005 | 0.014 | 0.015* | 0.018* | 0.010 | 0.028** |
| | (0.01) | (0.01) | (0.01) | (0.01) | (0.01) | (0.01) |
| p-value | 0.420 | 0.332 | 0.083 | 0.092 | 0.260 | 0.020 |
| Observations | 7605 | 7605 | 7605 | 7605 | 7605 | 7605 |
| Taxes (Not Progressive) | -0.025 | -0.013 | -0.007 | -0.019 | -0.024 | -0.038* |
| | (0.01) | (0.01) | (0.01) | (0.01) | (0.01) | (0.02) |
| p-value | 0.159 | 0.387 | 0.454 | 0.180 | 0.126 | 0.088 |
| Observations | 7435 | 7435 | 7435 | 7435 | 7435 | 7435 |
| Transfers (Progressive) | -0.005 | 0.015 | 0.012 | -0.008 | 0.008 | 0.009 |
| | (0.01) | (0.01) | (0.01) | (0.01) | (0.00) | (0.02) |
| p-value | 0.683 | 0.144 | 0.314 | 0.493 | 0.121 | 0.610 |
| Observations | 11318 | 11318 | 11318 | 11318 | 11318 | 11318 |
| Transfers (Not Progressive) | -0.014 | 0.002 | 0.002 | -0.005 | -0.007 | -0.008 |
| | (0.01) | (0.01) | (0.00) | (0.02) | (0.01) | (0.00) |
| p-value | 0.358 | 0.899 | 0.567 | 0.848 | 0.449 | 0.137 |
| Observations | 3810 | 3810 | 3810 | 3810 | 3810 | 3810 |
| Combined (Progressive) | -0.001 | 0.011 | 0.014 | 0.010 | 0.011* | 0.019 |
| | (0.01) | (0.01) | (0.01) | (0.01) | (0.00) | (0.01) |
| p-value | 0.924 | 0.401 | 0.169 | 0.294 | 0.068 | 0.196 |
| Observations | 11066 | 11066 | 11066 | 11066 | 11066 | 11066 |
| Combined (Not progressive) | -0.019 | -0.025 | 0.011 | -0.001 | -0.020 | -0.018 |
| | (0.01) | (0.01) | (0.00) | (0.02) | (0.02) | (0.02) |
| p-value | 0.297 | 0.168 | 0.132 | 0.977 | 0.454 | 0.444 |
| Observations | 3769 | 3769 | 3769 | 3769 | 3769 | 3769 |

Note: This table shows the overall impact of each of the treatments (without weights) relative to the control group, where countries are pooled based on whether the tax and/or transfer system is progressive. This table is directly comparable to Table 1 in Section 4 of the paper. This table is based on Equation 7 in Section 3 of the paper. * $p < 0.1$, ** $p < 0.05$, *** $p < 0.01$. *Direct:* Based on Q14, which asks whether respondents would not pay tax if they knew they would not get caught (variable takes value of 0 if they select "Strongly Agree" or "Agree" and 1 otherwise). *Punishable:* Based on Q15, which asks respondents their views about people not paying tax (variable takes value of 1 if they select "This is wrong and punishable" and 0 otherwise). *Important:* Based on Q16, which asks respondents whether it is important for people to pay tax (variable takes value of 1 if they select "Strongly Agree" or "Agree" and 0 otherwise). *Right to Tax:* Based on Q17, which asks respondents whether the government always has a right to make people pay tax (variable takes value of 1 if they select "Strongly Agree" or "Agree" and 0 otherwise). *Do not Refuse:* Based on Q18, which asks whether people should refuse to pay taxes until they receive more government transfers (variable takes value of 1 if they select "Strongly Disagree" or "Disagree" and 1 otherwise). *INDEX:* An unweighted average of the Z-scores of all five outcome variables, oriented so that a higher index means higher tax morale.

Table A4: Share of participants using a smartphone at various stages of the survey

| | Exposed to survey (%) | Began experiment (%) | Completed survey (%) |
|---|---|---|---|
| Colombia | 62.2 | 54.0 | 53.8 |
| Ghana | 50.4 | 66.7 | 66.6 |
| Indonesia | 72.4 | 78.0 | 77.2 |
| Jordan | 79.9 | 73.0 | 72.5 |
| Mexico | 62.5 | 52.6 | 52.5 |
| South Africa | 64.7 | 63.2 | 63.0 |
| Sri Lanka | 75.9 | 70.7 | 70.2 |
| Tanzania | 83.6 | 81.2 | 80.7 |

Note: This table shows the share of participants using a smartphone that were exposed to the survey, begin the survey experiment and completed the survey.

Table A5: Overall effects of the treatments (with device type weights)

| | Direct<br>b/se/p | Punishable<br>b/se/p | Important<br>b/se/p | Right to Tax<br>b/se/p | Refuse to Pay<br>b/se/p | INDEX<br>b/se/p |
|---|---|---|---|---|---|---|
| Taxes (Progressive) | 0.003 | 0.014 | 0.014 | 0.017 | 0.010 | 0.027** |
| | (0.01) | (0.01) | (0.01) | (0.01) | (0.01) | (0.01) |
| p-value | 0.637 | 0.370 | 0.102 | 0.108 | 0.286 | 0.020 |
| Observations | 7605 | 7605 | 7605 | 7605 | 7605 | 7605 |
| Taxes (Not Progressive) | -0.026 | -0.015 | -0.007 | -0.019 | -0.025 | -0.039* |
| | (0.01) | (0.01) | (0.01) | (0.01) | (0.01) | (0.01) |
| p-value | 0.147 | 0.352 | 0.383 | 0.179 | 0.123 | 0.079 |
| Observations | 7435 | 7435 | 7435 | 7435 | 7435 | 7435 |
| Transfers (Progressive) | -0.007 | 0.015 | 0.011 | -0.009 | 0.007 | 0.007 |
| | (0.01) | (0.01) | (0.01) | (0.01) | (0.00) | (0.02) |
| p-value | 0.583 | 0.162 | 0.362 | 0.487 | 0.134 | 0.683 |
| Observations | 11318 | 11318 | 11318 | 11318 | 11318 | 11318 |
| Transfers (Not Progressive) | -0.019 | -0.002 | 0.002 | -0.008 | -0.007 | -0.013 |
| | (0.01) | (0.01) | (0.00) | (0.03) | (0.01) | (0.01) |
| p-value | 0.190 | 0.876 | 0.674 | 0.797 | 0.442 | 0.267 |
| Observations | 3810 | 3810 | 3810 | 3810 | 3810 | 3810 |
| Combined (Progressive) | -0.004 | 0.008 | 0.012 | 0.010 | 0.011* | 0.016 |
| | (0.01) | (0.01) | (0.01) | (0.01) | (0.01) | (0.01) |
| p-value | 0.625 | 0.507 | 0.210 | 0.312 | 0.080 | 0.254 |
| Observations | 11066 | 11066 | 11066 | 11066 | 11066 | 11066 |
| Combined (Not progressive) | -0.021 | -0.027 | 0.012 | -0.002 | -0.021 | -0.021 |
| | (0.01) | (0.01) | (0.00) | (0.02) | (0.02) | (0.02) |
| p-value | 0.327 | 0.137 | 0.182 | 0.932 | 0.465 | 0.453 |
| Observations | 3769 | 3769 | 3769 | 3769 | 3769 | 3769 |

Note: This table shows the overall impact of each of the treatments (with device type weights) relative to the control group, where countries are pooled based on whether the tax and/or transfer system is progressive. This table is directly comparable to Table 1 in Section 4 of the paper. This table is based on Equation 7 in Section 3 of the paper. * $p < 0.1$, ** $p < 0.05$, *** $p < 0.01$. *Direct:* Based on Q14, which asks whether respondents would not pay tax if they knew they would not get caught (variable takes value of 0 if they select "Strongly Agree" or "Agree" and 1 otherwise). *Punishable:* Based on Q15, which asks respondents their views about people not paying tax (variable takes value of 1 if they select "This is wrong and punishable" and 0 otherwise). *Important:* Based on Q16, which asks respondents whether it is important for people to pay tax (variable takes value of 1 if they select "Strongly Agree" or "Agree" and 0 otherwise). *Right to Tax:* Based on Q17, which asks respondents whether the government always has a right to make people pay tax (variable takes value of 1 if they select "Strongly Agree" or "Agree" and 0 otherwise). *Do not Refuse:* Based on Q18, which asks whether people should refuse to pay taxes until they receive more government transfers (variable takes value of 1 if they select "Strongly Disagree" or "Disagree" and 1 otherwise). *INDEX:* An unweighted average of the Z-scores of all five outcome variables, oriented so that a higher index means higher tax morale.

Table A6: Balance table for the taxes treatment group relative to the control group

| | CO | GH | ID | JO | LK | MX | TZ | ZA |
|---|---|---|---|---|---|---|---|---|
| | b/se/p | b/se/p | b/se/p | b/se/p | b/se/p | b/se/p | b/se/p | b/se/p |
| Male | 0.000 | -0.073*** | 0.032 | 0.009 | -0.054* | -0.013 | 0.017 | 0.034 |
| | (0.02) | (0.03) | (0.03) | (0.02) | (0.03) | (0.02) | (0.03) | (0.02) |
| p-value | 0.983 | 0.007 | 0.198 | 0.714 | 0.058 | 0.596 | 0.510 | 0.151 |
| 18-34 years | -0.014 | 0.001 | -0.015 | -0.008 | 0.013 | 0.037 | 0.011 | -0.019 |
| | (0.02) | (0.03) | (0.03) | (0.03) | (0.03) | (0.02) | (0.03) | (0.02) |
| p-value | 0.548 | 0.976 | 0.572 | 0.742 | 0.594 | 0.118 | 0.713 | 0.437 |
| Sec edu or less | -0.007 | -0.026 | 0.021 | 0.003 | -0.025 | -0.020 | -0.079*** | 0.010 |
| | (0.02) | (0.02) | (0.03) | (0.02) | (0.02) | (0.03) | (0.02) | (0.02) |
| p-value | 0.767 | 0.267 | 0.444 | 0.884 | 0.300 | 0.460 | 0.001 | 0.676 |
| Large city | 0.046* | 0.043* | 0.001 | 0.011 | -0.031 | 0.018 | 0.010 | -0.015 |
| | (0.03) | (0.02) | (0.02) | (0.02) | (0.03) | (0.03) | (0.02) | (0.03) |
| p-value | 0.072 | 0.071 | 0.961 | 0.669 | 0.230 | 0.489 | 0.660 | 0.554 |
| Working | 0.008 | -0.031 | -0.009 | -0.023 | -0.014 | -0.005 | 0.036 | -0.041 |
| | (0.02) | (0.02) | (0.02) | (0.02) | (0.03) | (0.02) | (0.02) | (0.03) |
| p-value | 0.741 | 0.200 | 0.715 | 0.350 | 0.569 | 0.845 | 0.123 | 0.102 |
| Believe B40 | -0.005 | 0.009 | 0.052* | 0.004 | 0.030 | -0.007 | 0.005 | -0.027 |
| | (0.02) | (0.03) | (0.03) | (0.02) | (0.03) | (0.03) | (0.03) | (0.02) |
| p-value | 0.846 | 0.729 | 0.062 | 0.876 | 0.231 | 0.806 | 0.846 | 0.262 |
| Observations | 1923 | 1878 | 1864 | 1887 | 1799 | 1874 | 1930 | 1885 |
| F-statistic | 0.774 | 2.282 | 1.315 | 0.193 | 1.419 | 0.673 | 2.608 | 0.924 |

Note: This table presents the results of an OLS regression whereby the dependent variable is a dummy variable based on whether a respondent received the taxes treatment and the independent variables are characteristics of respondents. * $p < 0.1$, ** $p < 0.05$, *** $p < 0.01$. *CO:* Colombia. *GH:* Ghana. *ID:* Indonesia. *JO:* Jordan. *LK:* Sri Lanka. *MX:* Mexico. *TZ:* Tanzania. *ZA:* South Africa. *Male:* Based on Q0, which asks respondents whether they are male or female (variable takes value of 1 if they select "Male" and 0 otherwise). *18-34 years:* Based on Q0, which also asks respondents their age (variable takes value of 1 if they select between 18-34 years and 0 if they select 35 or older, noting respondents under the age of 18 years were automatically excluded). *Sec edu or less:* Based on Q1, which asks whether respondents their level of education (variable takes value of 1 if they select "Primary or less" or "Secondary" and 0 otherwise). *Large city:* Based on Q2, which asks respondents about where they live (variable takes value of 1 if they select "Large city" and 0 otherwise). *Working:* Based on Q3, which asks whether respondents their current employment status (variable takes value of 1 if they select "Employee" or "Self employed" and 0 otherwise). *Believe B40:* Based on Q5, which asks respondents about their households place in the national income distribution (variable takes value of 1 if they select "Poorest group" or "Second poorest group" and 0 otherwise).

Table A7: Balance table for the transfers treatment group relative to the control group

| | CO | GH | ID | JO | LK | MX | TZ | ZA |
|---|---|---|---|---|---|---|---|---|
| | b/se/p | b/se/p | b/se/p | b/se/p | b/se/p | b/se/p | b/se/p | b/se/p |
| Male | 0.015 | -0.032 | -0.015 | 0.020 | -0.050* | 0.005 | 0.077*** | -0.016 |
| | (0.02) | (0.03) | (0.02) | (0.02) | (0.03) | (0.02) | (0.03) | (0.02) |
| p-value | 0.522 | 0.258 | 0.535 | 0.405 | 0.075 | 0.831 | 0.002 | 0.504 |
| 18-34 years | -0.021 | -0.001 | 0.013 | -0.017 | 0.032 | 0.006 | 0.002 | -0.010 |
| | (0.02) | (0.03) | (0.03) | (0.03) | (0.02) | (0.02) | (0.03) | (0.02) |
| p-value | 0.382 | 0.972 | 0.629 | 0.509 | 0.198 | 0.805 | 0.930 | 0.687 |
| Sec edu or less | -0.029 | 0.004 | -0.012 | 0.047** | -0.038 | -0.049* | -0.066*** | 0.036 |
| | (0.02) | (0.02) | (0.03) | (0.02) | (0.02) | (0.03) | (0.02) | (0.02) |
| p-value | 0.220 | 0.881 | 0.654 | 0.047 | 0.117 | 0.070 | 0.004 | 0.133 |
| Large city | -0.006 | 0.015 | 0.025 | 0.018 | -0.041 | 0.005 | 0.036 | -0.033 |
| | (0.03) | (0.02) | (0.02) | (0.02) | (0.03) | (0.03) | (0.02) | (0.03) |
| p-value | 0.816 | 0.540 | 0.286 | 0.454 | 0.105 | 0.834 | 0.120 | 0.213 |
| Working | 0.009 | -0.001 | 0.047* | 0.017 | -0.006 | -0.021 | -0.002 | 0.019 |
| | (0.02) | (0.02) | (0.02) | (0.02) | (0.03) | (0.03) | (0.02) | (0.02) |
| p-value | 0.707 | 0.970 | 0.051 | 0.497 | 0.820 | 0.399 | 0.934 | 0.442 |
| Believe B40 | -0.028 | -0.009 | 0.034 | -0.007 | 0.018 | 0.019 | 0.013 | 0.019 |
| | (0.02) | (0.03) | (0.03) | (0.02) | (0.03) | (0.03) | (0.03) | (0.02) |
| p-value | 0.244 | 0.735 | 0.229 | 0.769 | 0.465 | 0.500 | 0.632 | 0.427 |
| Observations | 1905 | 1837 | 1901 | 1917 | 1849 | 1873 | 1973 | 1873 |
| F-statistic | 1.029 | 0.331 | 1.119 | 1.129 | 1.756 | 0.689 | 3.566 | 0.951 |

Note: This table presents the results of an OLS regression whereby the dependent variable is a dummy variable based on whether a respondent received the transfers treatment and the independent variables are characteristics of respondents. * $p < 0.1$, ** $p < 0.05$, *** $p < 0.01$. *CO:* Colombia. *GH:* Ghana. *ID:* Indonesia. *JO:* Jordan. *LK:* Sri Lanka. *MX:* Mexico. *TZ:* Tanzania. *ZA:* South Africa. *Male:* Based on Q0, which asks respondents whether they are male or female (variable takes value of 1 if they select "Male" and 0 otherwise). *18-34 years:* Based on Q0, which also asks respondents their age (variable takes value of 1 if they select between 18-34 years and 0 if they select 35 or older, noting respondents under the age of 18 years were automatically excluded). *Sec edu or less:* Based on Q1, which asks whether respondents their level of education (variable takes value of 1 if they select "Primary or less" or "Secondary" and 0 otherwise). *Large city:* Based on Q2, which asks respondents about where they live (variable takes value of 1 if they select "Large city" and 0 otherwise). *Working:* Based on Q3, which asks whether respondents their current employment status (variable takes value of 1 if they select "Employee" or "Self employed" and 0 otherwise). *Believe B40:* Based on Q5, which asks respondents about their households place in the national income distribution (variable takes value of 1 if they select "Poorest group" or "Second poorest group" and 0 otherwise).

Table A8: Balance table for the combined treatment group relative to the control group

| | CO | GH | ID | JO | LK | MX | TZ | ZA |
|---|---|---|---|---|---|---|---|---|
| | b/se/p | b/se/p | b/se/p | b/se/p | b/se/p | b/se/p | b/se/p | b/se/p |
| Male | -0.014 | -0.035 | 0.035 | 0.021 | -0.070** | -0.004 | 0.034 | 0.010 |
| | (0.02) | (0.03) | (0.03) | (0.02) | (0.03) | (0.02) | (0.03) | (0.02) |
| p-value | 0.563 | 0.203 | 0.157 | 0.396 | 0.012 | 0.880 | 0.184 | 0.680 |
| 18-34 years | -0.026 | -0.014 | -0.004 | -0.016 | -0.000 | 0.004 | -0.019 | -0.022 |
| | (0.02) | (0.03) | (0.03) | (0.03) | (0.02) | (0.02) | (0.03) | (0.02) |
| p-value | 0.275 | 0.633 | 0.881 | 0.540 | 0.995 | 0.872 | 0.510 | 0.376 |
| Sec edu or less | 0.016 | -0.037 | -0.020 | -0.009 | 0.010 | -0.011 | -0.056** | 0.024 |
| | (0.02) | (0.02) | (0.03) | (0.02) | (0.02) | (0.03) | (0.02) | (0.02) |
| p-value | 0.510 | 0.125 | 0.473 | 0.719 | 0.668 | 0.696 | 0.018 | 0.321 |
| Large city | 0.002 | 0.039 | -0.019 | -0.005 | -0.055** | 0.017 | -0.037 | -0.009 |
| | (0.03) | (0.02) | (0.02) | (0.02) | (0.03) | (0.03) | (0.02) | (0.03) |
| p-value | 0.923 | 0.101 | 0.416 | 0.825 | 0.029 | 0.514 | 0.114 | 0.728 |
| Working | 0.033 | -0.051** | -0.016 | -0.034 | 0.019 | 0.019 | 0.004 | -0.023 |
| | (0.02) | (0.02) | (0.02) | (0.03) | (0.02) | (0.03) | (0.02) | (0.03) |
| p-value | 0.184 | 0.034 | 0.502 | 0.180 | 0.451 | 0.449 | 0.862 | 0.357 |
| Believe B40 | -0.035 | -0.002 | 0.049* | -0.013 | 0.017 | 0.014 | 0.010 | -0.031 |
| | (0.02) | (0.03) | (0.03) | (0.02) | (0.03) | (0.03) | (0.03) | (0.02) |
| p-value | 0.156 | 0.947 | 0.078 | 0.589 | 0.493 | 0.619 | 0.708 | 0.210 |
| Observations | 1849 | 1900 | 1878 | 1865 | 1850 | 1794 | 1869 | 1830 |
| F-statistic | 1.108 | 1.927 | 1.207 | 0.442 | 1.939 | 0.237 | 1.656 | 0.604 |

Note: This table presents the results of an OLS regression whereby the dependent variable is a dummy variable based on whether a respondent received the combined treatment and the independent variables are characteristics of respondents. * $p < 0.1$, ** $p < 0.05$, *** $p < 0.01$. *CO:* Colombia. *GH:* Ghana. *ID:* Indonesia. *JO:* Jordan. *LK:* Sri Lanka. *MX:* Mexico. *TZ:* Tanzania. *ZA:* South Africa. *Male:* Based on Q0, which asks respondents whether they are male or female (variable takes value of 1 if they select "Male" and 0 otherwise). *18-34 years:* Based on Q0, which also asks respondents their age (variable takes value of 1 if they select between 18-34 years and 0 if they select 35 or older, noting respondents under the age of 18 years were automatically excluded) . *Sec edu or less:* Based on Q1, which asks whether respondents their level of education (variable takes value of 1 if they select "Primary or less" or "Secondary" and 0 otherwise). *Large city:* Based on Q2, which asks respondents about where they live (variable takes value of 1 if they select "Large city" and 0 otherwise). *Working:* Based on Q3, which asks whether respondents their current employment status (variable takes value of 1 if they select "Employee" or "Self employed" and 0 otherwise). *Believe B40:* Based on Q5, which asks respondents about their households place in the national income distribution (variable takes value of 1 if they select "Poorest group" or "Second poorest group" and 0 otherwise).

Table A9: Characteristics associated with higher tax morale

| | Direct<br>b/se | Punishable<br>b/se | Important<br>b/se | Right to Tax<br>b/se | Do not Refuse<br>b/se | INDEX<br>b/se |
|---|---|---|---|---|---|---|
| Smartphone | 0.018 | 0.029 | 0.059** | 0.034** | 0.006 | 0.063** |
| | (0.02) | (0.02) | (0.02) | (0.01) | (0.02) | (0.02) |
| Male | -0.017 | 0.033 | 0.077*** | 0.045** | 0.021 | 0.069* |
| | (0.02) | (0.02) | (0.02) | (0.01) | (0.03) | (0.03) |
| 18-34 years | -0.080*** | -0.071*** | -0.037 | -0.063** | -0.088*** | -0.140*** |
| | (0.02) | (0.02) | (0.03) | (0.02) | (0.01) | (0.04) |
| Sec edu or less | -0.051** | -0.041 | -0.021 | -0.030* | -0.039* | -0.076*** |
| | (0.02) | (0.03) | (0.01) | (0.01) | (0.02) | (0.02) |
| Large city | 0.027** | -0.010 | 0.021 | 0.035 | -0.008 | 0.029 |
| | (0.01) | (0.01) | (0.02) | (0.02) | (0.01) | (0.02) |
| Working | -0.019 | 0.024 | -0.008 | 0.017 | 0.009 | 0.010 |
| | (0.02) | (0.02) | (0.01) | (0.01) | (0.02) | (0.03) |
| Poorest quintile | 0.048 | 0.057 | -0.079 | 0.042 | -0.006 | 0.029 |
| | (0.06) | (0.06) | (0.05) | (0.04) | (0.05) | (0.08) |
| Second poorest quintile | 0.151** | 0.036 | -0.118* | 0.080 | -0.017 | 0.060 |
| | (0.06) | (0.05) | (0.06) | (0.06) | (0.05) | (0.09) |
| Middle quintile | 0.135* | 0.075* | -0.086 | 0.116** | -0.001 | 0.106 |
| | (0.06) | (0.04) | (0.05) | (0.05) | (0.04) | (0.08) |
| Second richest quintile | 0.050 | 0.099 | -0.093 | 0.053 | 0.043 | 0.064 |
| | (0.07) | (0.05) | (0.06) | (0.05) | (0.06) | (0.09) |
| Observations | 7933 | 7933 | 7933 | 7933 | 7933 | 7933 |

Note: This table presents the results of OLS regressions whereby the dependent variable is based on various measures of respondents (in the control group) willingness to pay tax and the independent variables are characteristics of respondents. Country fixed effects are used. * $p < 0.1$, ** $p < 0.05$, *** $p < 0.01$. *Direct:* Based on Q14, which asks whether respondents would not pay tax if they knew they would not get caught (variable takes value of 0 if they select "Strongly Agree" or "Agree" and 1 otherwise). *Punishable:* Based on Q15, which asks respondents their views about people not paying tax (variable takes value of 1 if they select "This is wrong and punishable" and 0 otherwise). *Important:* Based on Q16, which asks respondents whether it is important for people to pay tax (variable takes value of 1 if they select "Strongly Agree" or "Agree" and 0 otherwise). *Right to Tax:* Based on Q17, which asks respondents whether the government always has a right to make people pay tax (variable takes value of 1 if they select "Strongly Agree" or "Agree" and 0 otherwise). *Do not Refuse:* Based on Q18, which asks whether people should refuse to pay taxes until they receive more government transfers (variable takes value of 1 if they select "Strongly Disagree" or "Disagree" and 1 otherwise). *INDEX:* An unweighted average of the Z-scores of all five outcome variables, oriented so that a higher index means higher tax morale. *Smartphone:* Based on data provided by survey firm (variable takes value of 1 if they accessed the survey via smartphone and 0 otherwise). *Male:* Based on Q0, which asks respondents whether they are male or female (variable takes value of 1 if they select "Male" and 0 otherwise). *18-34 years:* Based on Q0, which also asks respondents their age (variable takes value of 1 if they select between 18-34 years and 0 if they select 35 or older, noting respondents under the age of 18 years were automatically excluded) *Sec edu or less:* Based on Q1, which asks whether respondents their level of education (variable takes value of 1 if they select "Primary or less" or "Secondary" and 0 otherwise). *Large city:* Based on Q2, which asks respondents about where they live (variable takes value of 1 if they select "Large city" and 0 otherwise). *Working:* Based on Q3, which asks whether respondents their current employment status (variable takes value of 1 if they select "Employee" or "Self employed" and 0 otherwise). *Poorest quintile:* Based on Q5, which asks respondents about their households place in the national income distribution (variable takes value of 1 if they selected the "Poorest group" or and 0 if they selected the "Richest group"). *Second poorest quintile:* Based on Q5, which asks respondents about their households place in the national income distribution (variable takes value of 1 if they selected the "Second poorest group" or and 0 if they selected the "Richest group"). *Middle quintile:* Based on Q5, which asks respondents about their households place in the national income distribution (variable takes value of 1 if they selected the "Middle group" or and 0 if they selected the "Richest group"). *Second richest quintile:* Based on Q5, which asks respondents about their households place in the national income distribution (variable takes value of 1 if they selected the "Second richest group" or and 0 if they selected the "Richest group").

Table A10: Average measure of tax morale compared to other cross-country measures

| Country | Average Tax Morale | Corruption Perceptions Index | Rule of Law Index | Believing the rich are selfish |
|---|---|---|---|---|
| Colombia | 0.47 | 39 | 36 | 3.2 |
| Ghana | 0.62 | 43 | 33 | N/A |
| Indonesia | 0.52 | 34 | 24 | 2.9 |
| Jordan | 0.34 | 47 | 50 | 3.3 |
| Mexico | 0.49 | 31 | 22 | 3.3 |
| South Africa | 0.5 | 43 | 42 | 3.8 |
| Sri Lanka | 0.65 | 36 | 33 | 3.1 |
| Tanzania | 0.53 | 38 | 33 | 3.6 |
| Cross-country correlation with Tax Morale | | -0.367 | -0.503 | -0.156 |

This table shows how the average measure of tax morale compares to other cross-country measures examining somewhat related topics of corruption (Transparency International, 2023), rule of law (World Justice Project, 2023) and beliefs about the rich being selfish in each country (Almas et al., 2022).

Table A11: Characteristics associated with believing taxes are progressive

| | CO | GH | ID | JO | LK | MX | TZ | ZA |
|---|---|---|---|---|---|---|---|---|
| | b/se | b/se | b/se | b/se | b/se | b/se | b/se | b/se |
| Male | 0.010 | 0.050*** | -0.032 | 0.041** | -0.041 | 0.017 | -0.007 | -0.026 |
| | (0.02) | (0.02) | (0.02) | (0.02) | (0.03) | (0.02) | (0.02) | (0.02) |
| 18-34 years | -0.012 | 0.015 | 0.001 | 0.041** | 0.066*** | 0.015 | 0.019 | 0.068*** |
| | (0.02) | (0.02) | (0.02) | (0.02) | (0.02) | (0.02) | (0.02) | (0.02) |
| Sec edu or less | 0.045** | 0.083*** | 0.026 | 0.049*** | 0.087*** | 0.085*** | 0.063*** | 0.011 |
| | (0.02) | (0.02) | (0.03) | (0.02) | (0.02) | (0.02) | (0.02) | (0.02) |
| Large city | -0.001 | 0.002 | -0.004 | 0.005 | 0.027 | -0.006 | -0.006 | -0.031 |
| | (0.02) | (0.02) | (0.02) | (0.02) | (0.03) | (0.02) | (0.02) | (0.02) |
| Working | -0.022 | 0.040** | 0.016 | 0.027 | 0.009 | -0.021 | -0.015 | 0.028 |
| | (0.02) | (0.02) | (0.02) | (0.02) | (0.02) | (0.02) | (0.02) | (0.02) |
| Second poorest quintile | -0.121*** | -0.125*** | -0.036 | -0.154*** | -0.053 | -0.075* | -0.056 | -0.042 |
| | (0.03) | (0.04) | (0.04) | (0.02) | (0.04) | (0.04) | (0.04) | (0.03) |
| Middle quintile | -0.062** | -0.092*** | -0.032 | -0.071*** | -0.007 | -0.018 | -0.008 | 0.017 |
| | (0.03) | (0.03) | (0.04) | (0.02) | (0.04) | (0.04) | (0.04) | (0.03) |
| Second richest quintile | 0.195*** | 0.088** | -0.060 | 0.057 | 0.041 | 0.101* | 0.126** | 0.240*** |
| | (0.06) | (0.04) | (0.09) | (0.05) | (0.06) | (0.06) | (0.06) | (0.04) |
| Richest quintile | 0.250*** | 0.163*** | -0.041 | 0.080* | 0.232*** | 0.130* | 0.214*** | 0.134** |
| | (0.07) | (0.05) | (0.10) | (0.05) | (0.08) | (0.07) | (0.07) | (0.07) |
| Prefer lower inequality | 0.011 | 0.082*** | 0.125*** | 0.057*** | 0.103*** | 0.030 | 0.108*** | 0.075*** |
| | (0.02) | (0.02) | (0.02) | (0.02) | (0.03) | (0.02) | (0.02) | (0.02) |
| Pay large share of income in tax | 0.058** | 0.043* | 0.095*** | -0.058*** | -0.041 | 0.104*** | 0.067*** | 0.061*** |
| | (0.02) | (0.02) | (0.03) | (0.02) | (0.03) | (0.02) | (0.02) | (0.02) |
| Net contributor to tax and transfer system | 0.060** | 0.152*** | 0.233*** | 0.145*** | 0.239*** | 0.173*** | 0.176*** | 0.092*** |
| | (0.02) | (0.02) | (0.03) | (0.02) | (0.03) | (0.02) | (0.02) | (0.02) |
| Observations | 3687 | 3607 | 3665 | 3645 | 3624 | 3629 | 3622 | 3658 |

Note: This table presents the characteristics that are associated with respondents believing taxes are progressive. Specifically, the table presents the results of an OLS regression in each country whereby the dependent variable is a dummy variable based on whether or not respondents prefer progressive taxes and the independent variables are characteristics of respondents. Believing taxes are progressive is based on Q8, which asks respondents whether they think that richer households currently pay a higher share of their income in tax than poorer households. * $p < 0.1$, ** $p < 0.05$, *** $p < 0.01$. *CO:* Colombia. *GH:* Ghana. *ID:* Indonesia. *JO:* Jordan. *LK:* Sri Lanka. *MX:* Mexico. *TZ:* Tanzania. *ZA:* South Africa. *Male:* Based on Q0, which asks respondents whether they are male or female (variable takes value of 1 if they select "Male" and 0 otherwise). *18-34 years:* Based on Q0, which also asks respondents their age (variable takes value of 1 if they select between 18-34 years and 0 if they select 35 or older, noting respondents under the age of 18 years were automatically excluded) *Sec edu or less:* Based on Q1, which asks whether respondents their level of education (variable takes value of 1 if they select "Primary or less" or "Secondary" and 0 otherwise). *Large city:* Based on Q2, which asks respondents about where they live (variable takes value of 1 if they select "Large city" and 0 otherwise). *Working:* Based on Q3, which asks whether respondents their current employment status (variable takes value of 1 if they select "Employee" or "Self employed" and 0 otherwise). *Poorest quintile:* Based on Q5, which asks respondents about their households place in the national income distribution (variable takes value of 1 if they selected the "Poorest group" or and 0 if they selected the "Richest group"). *Second poorest quintile:* Based on Q5, which asks respondents about their households place in the national income distribution (variable takes value of 1 if they selected the "Second poorest group" or and 0 if they selected the "Richest group"). *Middle quintile:* Based on Q5, which asks respondents about their households place in the national income distribution (variable takes value of 1 if they selected the "Middle group" or and 0 if they selected the "Richest group"). *Second richest quintile:* Based on Q5, which asks respondents about their households place in the national income distribution (variable takes value of 1 if they selected the "Second richest group" or and 0 if they selected the "Richest group"). Beliefs about the share of household income that is paid in tax are based on Q6. Respondents' views about whether their household was a net contributor to the tax and transfer system is based on Q7. Respondents' views about inequality is based on Q4.

Table A12: Characteristics associated with preferring progressive taxes

| | CO | GH | ID | JO | LK | MX | TZ | ZA |
|---|---|---|---|---|---|---|---|---|
| | b/se | b/se | b/se | b/se | b/se | b/se | b/se | b/se |
| Male | 0.051*** | 0.029 | -0.024 | 0.056*** | 0.027 | -0.001 | 0.026 | 0.026 |
| | (0.02) | (0.02) | (0.02) | (0.02) | (0.02) | (0.02) | (0.02) | (0.02) |
| 18-34 years | 0.006 | -0.050** | -0.098*** | -0.070*** | -0.030 | -0.071*** | -0.033 | 0.025 |
| | (0.02) | (0.02) | (0.02) | (0.02) | (0.02) | (0.02) | (0.02) | (0.02) |
| Sec edu or less | 0.018 | 0.039** | -0.018 | 0.010 | -0.005 | 0.039* | 0.007 | 0.023 |
| | (0.02) | (0.02) | (0.03) | (0.02) | (0.02) | (0.02) | (0.02) | (0.02) |
| Large city | -0.001 | 0.007 | -0.012 | -0.000 | 0.032 | 0.016 | 0.018 | -0.024 |
| | (0.02) | (0.02) | (0.02) | (0.02) | (0.02) | (0.02) | (0.02) | (0.02) |
| Working | -0.025 | 0.031* | 0.014 | 0.029 | 0.018 | 0.060*** | 0.011 | 0.001 |
| | (0.02) | (0.02) | (0.02) | (0.02) | (0.02) | (0.02) | (0.02) | (0.02) |
| Second poorest quintile | -0.059** | -0.124*** | 0.070* | -0.060** | 0.014 | 0.051 | -0.041 | -0.070** |
| | (0.03) | (0.03) | (0.04) | (0.03) | (0.04) | (0.04) | (0.04) | (0.03) |
| Middle quintile | -0.030 | -0.125*** | 0.042 | -0.077*** | 0.010 | 0.014 | -0.021 | -0.083*** |
| | (0.03) | (0.03) | (0.04) | (0.02) | (0.03) | (0.04) | (0.03) | (0.02) |
| Second richest quintile | -0.073 | -0.145*** | -0.020 | -0.110** | -0.056 | -0.099* | 0.001 | -0.031 |
| | (0.06) | (0.04) | (0.06) | (0.05) | (0.07) | (0.06) | (0.05) | (0.05) |
| Richest quintile | -0.138** | -0.116*** | 0.011 | -0.070 | -0.008 | -0.144** | -0.049 | 0.011 |
| | (0.07) | (0.04) | (0.08) | (0.04) | (0.07) | (0.06) | (0.07) | (0.07) |
| Prefer lower inequality | 0.196*** | 0.193*** | 0.168*** | 0.174*** | 0.158*** | 0.219*** | 0.211*** | 0.206*** |
| | (0.02) | (0.02) | (0.02) | (0.02) | (0.03) | (0.02) | (0.02) | (0.02) |
| Pay large share of income in tax | 0.131*** | 0.079*** | 0.111*** | 0.063*** | 0.002 | 0.096*** | 0.092*** | 0.053** |
| | (0.02) | (0.02) | (0.03) | (0.02) | (0.03) | (0.02) | (0.02) | (0.02) |
| Net contributor to tax and transfer system | 0.119*** | 0.118*** | 0.239*** | 0.132*** | 0.249*** | 0.117*** | 0.131*** | 0.070*** |
| | (0.02) | (0.02) | (0.02) | (0.02) | (0.03) | (0.02) | (0.02) | (0.02) |
| Observations | 3687 | 3607 | 3665 | 3645 | 3624 | 3629 | 3622 | 3658 |

Note: This table presents the characteristics that are associated with respondents preferring progressive taxes. Specifically, the table presents the results of an OLS regression in each country whereby the dependent variable is a dummy variable based on whether or not respondents prefer progressive taxes and the independent variables are characteristics of respondents. Preferences about progressivity are based on Q9, which asks respondents whether they think that richer households should pay a higher share of their income in tax than poorer households. * $p < 0.1$, ** $p < 0.05$, *** $p < 0.01$. *CO:* Colombia. *GH:* Ghana. *ID:* Indonesia. *JO:* Jordan. *LK:* Sri Lanka. *MX:* Mexico. *TZ:* Tanzania. *ZA:* South Africa. *Male:* Based on Q0, which asks respondents whether they are male or female (variable takes value of 1 if they select "Male" and 0 otherwise). *18-34 years:* Based on Q0, which also asks respondents their age (variable takes value of 1 if they select between 18-34 years and 0 if they select 35 or older, noting respondents under the age of 18 years were automatically excluded) *Sec edu or less:* Based on Q1, which asks whether respondents their level of education (variable takes value of 1 if they select "Primary or less" or "Secondary" and 0 otherwise). *Large city:* Based on Q2, which asks respondents about where they live (variable takes value of 1 if they select "Large city" and 0 otherwise). *Working:* Based on Q3, which asks whether respondents their current employment status (variable takes value of 1 if they select "Employee" or "Self employed" and 0 otherwise). *Poorest quintile:* Based on Q5, which asks respondents about their households place in the national income distribution (variable takes value of 1 if they selected the "Poorest group" or and 0 if they selected the “Richest group”). *Second poorest quintile:* Based on Q5, which asks respondents about their households place in the national income distribution (variable takes value of 1 if they selected the "Second poorest group" or and 0 if they selected the “Richest group”). *Middle quintile:* Based on Q5, which asks respondents about their households place in the national income distribution (variable takes value of 1 if they selected the "Middle group" or and 0 if they selected the “Richest group”). *Second richest quintile:* Based on Q5, which asks respondents about their households place in the national income distribution (variable takes value of 1 if they selected the "Second richest group" or and 0 if they selected the “Richest group”). Beliefs about the share of household income that is paid in tax are based on Q6. Respondents’ views about whether their household was a net contributor to the tax and transfer system is based on Q7. Respondents’ views about inequality is based on Q4.

Table A13: Overall impact of the transfers treatment

| | Direct<br>b/se | Punishable<br>b/se | Important<br>b/se | Right to Tax<br>b/se | Do not Refuse<br>b/se | INDEX<br>b/se |
|---|---|---|---|---|---|---|
| **Panel A - Respondents in countries where transfers were progressive** | | | | | | |
| Believe progressive × Treated | 0.032 | 0.015 | 0.005 | -0.018 | 0.019 | 0.022 |
| | (0.03) | (0.01) | (0.02) | (0.03) | (0.02) | (0.03) |
| Believe not progressive × Treated | -0.032* | 0.005 | 0.024* | 0.011 | -0.006 | 0.001 |
| | (0.01) | (0.01) | (0.01) | (0.01) | (0.01) | (0.01) |
| p-value difference | 0.136 | 0.404 | 0.378 | 0.173 | 0.244 | 0.429 |
| Observations | 11318 | 11318 | 11318 | 11318 | 11318 | 11318 |
| | | | | | | |
| Prefer progressive × Treated | -0.000 | 0.023 | -0.001 | -0.005 | 0.006 | 0.010 |
| | (0.01) | (0.02) | (0.02) | (0.03) | (0.01) | (0.02) |
| Prefer not progressive × Treated | -0.015 | -0.011 | 0.039** | 0.001 | 0.001 | 0.006 |
| | (0.02) | (0.02) | (0.01) | (0.02) | (0.01) | (0.02) |
| p-value difference | 0.250 | 0.281 | 0.098 | 0.893 | 0.738 | 0.859 |
| Observations | 11318 | 11318 | 11318 | 11318 | 11318 | 11318 |
| **Panel B - Respondents in countries where transfers were not progressive** | | | | | | |
| Believe progressive × Treated | -0.010 | 0.037 | -0.022* | -0.012 | 0.006 | -0.007 |
| | (0.01) | (0.02) | (0.00) | (0.00) | (0.02) | (0.01) |
| Believe not progressive × Treated | 0.008 | -0.007 | 0.014 | 0.002 | -0.013 | 0.007 |
| | (0.00) | (0.01) | (0.01) | (0.04) | (0.02) | (0.02) |
| p-value difference | 0.532 | 0.123 | 0.286 | 0.791 | 0.715 | 0.699 |
| Observations | 3810 | 3810 | 3810 | 3810 | 3810 | 3810 |
| | | | | | | |
| Prefer progressive × Treated | -0.006 | -0.001 | -0.007 | -0.016 | -0.006 | -0.017 |
| | (0.01) | (0.01) | (0.01) | (0.01) | (0.01) | (0.01) |
| Prefer not progressive × Treated | 0.006 | 0.017 | 0.015 | 0.020 | -0.013** | 0.025 |
| | (0.02) | (0.02) | (0.03) | (0.06) | (0.00) | (0.04) |
| p-value difference | 0.714 | 0.227 | 0.651 | 0.617 | 0.671 | 0.493 |
| Observations | 3810 | 3810 | 3810 | 3810 | 3810 | 3810 |

Note: This table shows the heterogeneous effects of the transfers treatment based on respondents prior beliefs about and existing preferences regarding whether taxes were progressive, where countries are pooled based on whether or not transfers are actually progressive. This table is based on Equation 7 in Section 3 of the paper, except the regression analysis is conducted separately for respondents based on their prior beliefs and existing preferences. Beliefs about progressivity are based on Q8, which asks respondents whether they believe that richer households pay a higher share of their income in tax than poorer households. Preferences about progressivity are based on Q9, which asks respondents whether they think that richer households should pay a higher share of their income in tax than poorer households.* $p < 0.1$, ** $p < 0.05$, *** $p < 0.01$. See the notes to Table 1 for further variable definitions.

Table A14: Overall impact of each treatment on the Tax Morale index by country

| | CO | GH | ID | JO | LK | MX | TZ | ZA |
|---|---|---|---|---|---|---|---|---|
| | b/se/p | b/se/p | b/se/p | b/se/p | b/se/p | b/se/p | b/se/p | b/se/p |
| Taxes treatment | 0.058* | 0.013 | -0.020 | -0.042 | -0.045 | 0.029 | 0.040 | -0.071** |
| | (0.03) | (0.03) | (0.04) | (0.03) | (0.03) | (0.03) | (0.03) | (0.03) |
| p-value | 0.055 | 0.651 | 0.646 | 0.115 | 0.181 | 0.347 | 0.179 | 0.013 |
| Observations | 1923 | 1878 | 1864 | 1887 | 1799 | 1874 | 1930 | 1885 |
| Transfers treatment | 0.069** | -0.011 | 0.010 | -0.033 | -0.045 | 0.040 | 0.013 | -0.008 |
| | (0.03) | (0.03) | (0.04) | (0.03) | (0.03) | (0.03) | (0.03) | (0.03) |
| p-value | 0.022 | 0.718 | 0.799 | 0.222 | 0.194 | 0.215 | 0.676 | 0.783 |
| Observations | 1905 | 1837 | 1901 | 1917 | 1849 | 1873 | 1973 | 1873 |
| Combined treatment | 0.052* | -0.032 | 0.064 | 0.004 | 0.012 | 0.039 | 0.011 | -0.017 |
| | (0.03) | (0.03) | (0.04) | (0.03) | (0.04) | (0.03) | (0.03) | (0.03) |
| p-value | 0.080 | 0.233 | 0.117 | 0.880 | 0.730 | 0.236 | 0.726 | 0.574 |
| Observations | 1849 | 1900 | 1878 | 1865 | 1850 | 1794 | 1869 | 1830 |

Note: This table shows the overall impact of the treatments in each of the countries. This table is based on Equation 7 in Section 3 of the paper, except the regression analysis is conducted separately for each country (i.e. there are no country fixed effects). * $p < 0.1$, ** $p < 0.05$, *** $p < 0.01$. *CO:* Colombia. *GH:* Ghana. *ID:* Indonesia. *JO:* Jordan. *LK:* Sri Lanka. *MX:* Mexico. *TZ:* Tanzania. *ZA:* South Africa. *WTP tax INDEX:* An unweighted average of the Z-scores of all five outcome variables, oriented so that a higher index means higher tax morale.

Table A15 - Impact of the treatments on Tax Morale index across the income distribution

| | Q1<br>b/se/p | Q2<br>b/se/p | Q3<br>b/se/p | Q4<br>b/se/p | Q5<br>b/se/p |
|---|---|---|---|---|---|
| Taxes (Progressive) | 0.066* | 0.047 | 0.033** | 0.102 | -0.115 |
| | (0.03) | (0.03) | (0.01) | (0.10) | (0.13) |
| p-value | 0.081 | 0.158 | 0.022 | 0.368 | 0.453 |
| Observations | 668 | 1454 | 4924 | 354 | 205 |
| Taxes (Not Progressive) | -0.026 | -0.042 | -0.065** | 0.034 | 0.005 |
| | (0.06) | (0.03) | (0.01) | (0.12) | (0.06) |
| p-value | 0.698 | 0.280 | 0.021 | 0.792 | 0.945 |
| Observations | 984 | 1672 | 4349 | 247 | 183 |
| Transfers (Progressive) | -0.047 | 0.030 | 0.005 | 0.041 | 0.054 |
| | (0.04) | (0.03) | (0.02) | (0.11) | (0.09) |
| p-value | 0.318 | 0.321 | 0.768 | 0.717 | 0.577 |
| Observations | 1360 | 2482 | 6850 | 378 | 248 |
| Transfers (Not Progressive) | 0.039 | 0.021 | 0.013 | -0.169 | -0.035 |
| | (0.05) | (0.09) | (0.01) | (0.17) | (0.06) |
| p-value | 0.559 | 0.852 | 0.460 | 0.502 | 0.642 |
| Observations | 294 | 661 | 2507 | 216 | 132 |
| Combined (Progressive) | 0.018 | 0.022 | 0.033 | -0.038 | -0.032 |
| | (0.04) | (0.03) | (0.02) | (0.14) | (0.12) |
| p-value | 0.638 | 0.522 | 0.113 | 0.797 | 0.799 |
| Observations | 1318 | 2413 | 6716 | 373 | 246 |
| Combined (Not progressive) | -0.023 | 0.029 | -0.008 | -0.077 | -0.030 |
| | (0.02) | (0.06) | (0.04) | (0.15) | (0.22) |
| p-value | 0.510 | 0.717 | 0.894 | 0.690 | 0.913 |
| Observations | 299 | 650 | 2489 | 207 | 124 |

Note:. This table shows the overall impact of the tax treatment on the willingness to pay tax index for each quintile, where countries are pooled based on whether the tax and/or transfer system is actually progressive. This table is based on Equation 7 in Section 3 of the paper, except the regression analysis is conducted separately for each quintile. * $p < 0.1$, ** $p < 0.05$, *** $p < 0.01$. *WTP tax index:* An unweighted average of the Z-scores of all five outcome variables, oriented so that a higher index means higher tax morale. *Q1:* Poorest quintile, based on answer to Q5. *Q2:* Second poorest quintile, based on answer to Q5. *Q3:* Middle quintile, based on answer to Q5. *Q4:* Second richest quintile, based on answer to Q5. *Q5:* Richest quintile, based on answer to Q5.

Table A16 – Heterogeneous effects of the treatments based on socio-economic status of respondents

| | Direct b/se | Punishable b/se | Important b/se | Right to Tax b/se | Do not Refuse b/se | INDEX b/se |
|---|---|---|---|---|---|---|
| **Panel A - Respondents with low socio-economic status** | | | | | | |
| Taxes (Progressive) | -0.004 | 0.000 | 0.040 | 0.054* | 0.030 | 0.060 |
| | (0.05) | (0.05) | (0.02) | (0.02) | (0.03) | (0.04) |
| Taxes (Not Progressive) | 0.008 | 0.012 | -0.015 | -0.007 | -0.001 | -0.003 |
| | (0.05) | (0.02) | (0.02) | (0.02) | (0.03) | (0.05) |
| | (0.03) | (0.01) | (0.02) | (0.01) | (0.02) | (0.01) |
| Transfers (Progressive) | -0.006 | 0.028* | 0.032 | 0.004 | 0.006 | 0.027 |
| | (0.04) | (0.01) | (0.02) | (0.03) | (0.01) | (0.03) |
| Transfers (Not Progressive) | 0.024 | -0.080** | 0.018 | 0.036 | -0.014 | 0.001 |
| | (0.09) | (0.00) | (0.03) | (0.01) | (0.02) | (0.06) |
| Combined (Progressive) | 0.009 | 0.029 | 0.018 | 0.005 | 0.000 | 0.025 |
| | (0.04) | (0.02) | (0.01) | (0.02) | (0.01) | (0.03) |
| Combined (Not Progressive) | -0.026 | -0.041* | 0.039 | 0.051 | -0.039** | 0.009 |
| | (0.04) | (0.00) | (0.01) | (0.04) | (0.00) | (0.03) |
| **Panel B - Respondents with medium or high socio-economic status** | | | | | | |
| Taxes (Progressive) | 0.011 | 0.026* | 0.020 | 0.009 | 0.010 | 0.033** |
| | (0.01) | (0.01) | (0.01) | (0.01) | (0.01) | (0.01) |
| Taxes (Not Progressive) | -0.031* | -0.035* | -0.011 | -0.033 | -0.036 | -0.061*** |
| | (0.01) | (0.01) | (0.02) | (0.02) | (0.03) | (0.01) |
| Transfers (Progressive) | -0.007 | 0.004 | 0.010 | -0.004 | 0.003 | 0.002 |
| | (0.01) | (0.01) | (0.01) | (0.01) | (0.01) | (0.02) |
| Transfers (Not Progressive) | -0.007 | 0.032 | -0.003 | -0.012 | -0.006 | 0.001 |
| | (0.02) | (0.01) | (0.00) | (0.03) | (0.01) | (0.00) |
| Combined (Progressive) | -0.002 | 0.001 | 0.020* | 0.024* | 0.015* | 0.024 |
| | (0.01) | (0.01) | (0.01) | (0.01) | (0.01) | (0.01) |
| Combined (Not Progressive) | -0.008 | -0.021 | 0.004 | -0.004 | -0.018 | -0.017 |
| | (0.02) | (0.01) | (0.01) | (0.02) | (0.02) | (0.02) |

Note: This table shows the heterogeneous treatment effects based on whether respondents had low or medium/high socio-economic status, where countries are pooled based on whether the tax and/or transfer system is actually progressive. This table is based on Equation 7 in Section 3 of the paper, except the regression analysis is conducted separately for respondents based on socio-economic status. Socio-economic status is based on respondents' education level and location (Q1 and Q2). * $p < 0.1$, ** $p < 0.05$, *** $p < 0.01$. *Direct:* Based on Q14, which asks whether respondents would not pay tax if they knew they would not get caught (variable takes value of 0 if they select "Strongly Agree" or "Agree" and 1 otherwise). *Punishable:* Based on Q15, which asks respondents their views about people not paying tax (variable takes value of 1 if they select "This is wrong and punishable" and 0 otherwise). *Important:* Based on Q16, which asks respondents whether it is important for people to pay tax (variable takes value of 1 if they select "Strongly Agree" or "Agree" and 0 otherwise). *Right to Tax:* Based on Q17, which asks respondents whether the government always has a right to make people pay tax (variable takes value of 1 if they select "Strongly Agree" or "Agree" and 0 otherwise). *Do not Refuse:* Based on Q18, which asks whether people should refuse to pay taxes until they receive more government transfers (variable takes value of 1 if they select "Strongly Disagree" or "Disagree" and 1 otherwise). *INDEX:* An unweighted average of the Z-scores of all five outcome variables, oriented so that a higher index means higher tax morale.

Table A17 – Heterogeneous effects of the treatments based on whether households paid a large share of their income in tax

| | Direct | Punishable | Important | Right to Tax | Do not Refuse | INDEX |
|---|---|---|---|---|---|---|
| | b/se | b/se | b/se | b/se | b/se | b/se |
| **Panel A - Respondents stated their household paid a large share of their income in tax** | | | | | | |
| Taxes (Progressive) | 0.006 | 0.038** | 0.024 | 0.015 | 0.017 | 0.042* |
| | (0.01) | (0.01) | (0.01) | (0.01) | (0.02) | (0.02) |
| Taxes (Not Progressive) | -0.025 | -0.022* | -0.020 | -0.045** | -0.017 | -0.054** |
| | (0.03) | (0.01) | (0.02) | (0.01) | (0.02) | (0.01) |
| Transfers (Progressive) | 0.006 | 0.013 | 0.007 | -0.011 | 0.024** | 0.016 |
| | (0.01) | (0.01) | (0.01) | (0.02) | (0.01) | (0.02) |
| Transfers (Not Progressive) | -0.021*** | -0.008 | -0.012** | -0.032* | -0.031 | -0.048 |
| | (0.00) | (0.03) | (0.00) | (0.00) | (0.01) | (0.02) |
| Combined (Progressive) | -0.001 | 0.005 | 0.002 | 0.003 | 0.023** | 0.014 |
| | (0.01) | (0.02) | (0.01) | (0.01) | (0.01) | (0.01) |
| Combined (Not Progressive) | - 0.047 | 0.003 | 0.008 | -0.002 | -0.031 | -0.026 |
| | (0.03) | (0.01) | (0.01) | (0.01) | (0.03) | (0.03) |
| **Panel B - Respondents stated their household did not pay a large share of their income in tax** | | | | | | |
| Taxes (Progressive) | 0.014 | -0.009 | 0.015 | 0.017 | 0.010 | 0.027 |
| | (0.02) | (0.05) | (0.02) | (0.03) | (0.02) | (0.04) |
| Taxes (Not Progressive) | -0.016 | -0.022 | -0.004 | -0.002 | -0.039 | -0.036 |
| | (0.04) | (0.01) | (0.01) | (0.03) | (0.03) | (0.03) |
| Transfers (Progressive) | -0.024 | 0.006 | 0.023 | 0.008 | -0.021 | -0.002 |
| | (0.02) | (0.02) | (0.02) | (0.01) | (0.01) | (0.02) |
| Transfers (Not Progressive) | 0.041 | 0.037 | 0.033 | 0.067 | 0.041* | 0.105 |
| | (0.01) | (0.01) | (0.03) | (0.10) | (0.01) | (0.06) |
| Combined (Progressive) | 0.003 | 0.015 | 0.044** | 0.044** | 0.002 | 0.045 |
| | (0.01) | (0.01) | (0.02) | (0.02) | (0.02) | (0.02) |
| Combined (Not Progressive) | 0.075* | -0.089 | 0.016* | 0.027 | 0.007 | 0.023 |
| | (0.01) | (0.06) | (0.00) | (0.07) | (0.03) | (0.01) |

Note: This table shows the heterogeneous treatment effects based on respondents beliefs about the share of their household income that is paid in tax, where countries are pooled based on whether the tax and/or transfer system is actually progressive. This table is based on Equation 7 in Section 3 of the paper, except the regression analysis is conducted separately for respondents based on their prior beliefs about the share of their household income that is paid in tax. Beliefs about the share of household income that is paid in tax are based on Q6. * $p < 0.1$, ** $p < 0.05$, *** $p < 0.01$. *Direct:* Based on Q14, which asks whether respondents would not pay tax if they knew they would not get caught (variable takes value of 0 if they select "Strongly Agree" or "Agree" and 1 otherwise). *Punishable:* Based on Q15, which asks respondents their views about people not paying tax (variable takes value of 1 if they select "This is wrong and punishable" and 0 otherwise). *Important:* Based on Q16, which asks respondents whether it is important for people to pay tax (variable takes value of 1 if they select "Strongly Agree" or "Agree" and 0 otherwise). *Right to Tax:* Based on Q17, which asks respondents whether the government always has a right to make people pay tax (variable takes value of 1 if they select "Strongly Agree" or "Agree" and 0 otherwise). *Do not Refuse:* Based on Q18, which asks whether people should refuse to pay taxes until they receive more government transfers (variable takes value of 1 if they select "Strongly Disagree" or "Disagree" and 1 otherwise). *INDEX:* An unweighted average of the Z-scores of all five outcome variables, oriented so that a higher index means higher tax morale.

Table A18 – Heterogeneous effects of the treatments based on whether respondents claimed their household was a net contributor to the tax and transfer system

| | Direct | Punishable | Important | Right to Tax | Do not Refuse | INDEX |
|---|---|---|---|---|---|---|
| | b/se | b/se | b/se | b/se | b/se | b/se |
| **Panel A - Respondents stated their household paid more in taxes than they received in transfers** | | | | | | |
| Taxes (Progressive) | 0.001 | 0.031** | 0.013 | 0.007 | 0.023 | 0.033** |
| | (0.02) | (0.01) | (0.01) | (0.01) | (0.02) | (0.01) |
| Taxes (Not Progressive) | -0.022* | -0.031* | -0.022 | -0.043* | -0.016 | -0.056** |
| | (0.01) | (0.01) | (0.02) | (0.02) | (0.02) | (0.02) |
| Transfers (Progressive) | 0.005 | 0.009 | 0.006 | -0.008 | 0.012 | 0.011 |
| | (0.01) | (0.01) | (0.02) | (0.02) | (0.01) | (0.02) |
| Transfers (Not Progressive) | -0.014 | 0.006 | -0.001 | -0.024* | -0.005 | -0.017** |
| | (0.01) | (0.01) | (0.00) | (0.00) | (0.01) | (0.00) |
| Combined (Progressive) | -0.009 | 0.002 | -0.009 | -0.001 | 0.015 | 0.000 |
| | (0.01) | (0.02) | (0.01) | (0.01) | (0.01) | (0.01) |
| Combined (Not Progressive) | -0.045 | -0.018* | 0.013 | -0.001 | -0.034 | -0.030 |
| | (0.03) | (0.00) | (0.02) | (0.02) | (0.02) | (0.02) |
| **Panel B - Respondents stated their household did not pay more in taxes than they received in transfers** | | | | | | |
| Taxes (Progressive) | 0.020 | 0.007 | 0.040* | 0.034 | -0.003 | 0.044 |
| | (0.01) | (0.04) | (0.02) | (0.02) | (0.01) | (0.02) |
| Taxes (Not Progressive) | 0.021 | -0.012 | -0.004 | -0.009 | -0.044 | -0.040 |
| | (0.03) | (0.01) | (0.03) | (0.03) | (0.04) | (0.04) |
| Transfers (Progressive) | -0.023 | 0.009 | 0.025 | 0.004 | -0.009 | 0.002 |
| | (0.03) | (0.02) | (0.01) | (0.02) | (0.02) | (0.03) |
| Transfers (Not Progressive) | 0.022 | 0.009 | 0.004 | 0.033 | -0.002 | 0.033 |
| | (0.02) | (0.03) | (0.02) | (0.07) | (0.01) | (0.02) |
| Combined (Progressive) | 0.016 | 0.017 | 0.058** | 0.045* | 0.009 | 0.059 |
| | (0.02) | (0.02) | (0.02) | (0.02) | (0.02) | (0.03) |
| Combined (Not Progressive) | 0.043 | -0.035 | 0.007 | 0.027 | 0.010 | 0.027 |
| | (0.02) | (0.02) | (0.02) | (0.05) | (0.02) | (0.01) |

Note: This table shows the heterogeneous treatment effects based on whether respondents claimed their household was a net contributor to the tax and transfer system, where countries are pooled based on whether the tax and/or transfer system is actually progressive. This table is based on Equation 7 in Section 3 of the paper, except the regression analysis is conducted separately based on whether respondents claimed their household was a net contributor to the tax and transfer system. Respondents' views about whether their household was a net contributor to the tax and transfer system is based on Q7. * $p < 0.1$, ** $p < 0.05$, *** $p < 0.01$. *Direct:* Based on Q14, which asks whether respondents would not pay tax if they knew they would not get caught (variable takes value of 0 if they select "Strongly Agree" or "Agree" and 1 otherwise). *Punishable:* Based on Q15, which asks respondents their views about people not paying tax (variable takes value of 1 if they select "This is wrong and punishable" and 0 otherwise). *Important:* Based on Q16, which asks respondents whether it is important for people to pay tax (variable takes value of 1 if they select "Strongly Agree" or "Agree" and 0 otherwise). *Right to Tax:* Based on Q17, which asks respondents whether the government always has a right to make people pay tax (variable takes value of 1 if they select "Strongly Agree" or "Agree" and 0 otherwise). *Do not Refuse:* Based on Q18, which asks whether people should refuse to pay taxes until they receive more government transfers (variable takes value of 1 if they select "Strongly Disagree" or "Disagree" and 1 otherwise). *INDEX:* An unweighted average of the Z-scores of all five outcome variables, oriented so that a higher index means higher tax morale.

Table A19 – Heterogeneous effects of the treatments based on whether respondents were working

| | Direct<br>b/se | Punishable<br>b/se | Important<br>b/se | Right to Tax<br>b/se | Do not Refuse<br>b/se | INDEX<br>b/se |
|---|---|---|---|---|---|---|
| **Panel A - Respondents stated that they were either an employee or self-employed** | | | | | | |
| Taxes (Progressive) | 0.008 | 0.015 | 0.015* | 0.019** | 0.012 | 0.030* |
| | (0.01) | (0.02) | (0.01) | (0.00) | (0.01) | (0.01) |
| Taxes (Not Progressive) | -0.038 | -0.039 | -0.016 | -0.014 | -0.054* | -0.069* |
| | (0.03) | (0.02) | (0.01) | (0.01) | (0.02) | (0.03) |
| Transfers (Progressive) | -0.004 | 0.003 | 0.013 | 0.016 | 0.001 | 0.012 |
| | (0.02) | (0.02) | (0.01) | (0.02) | (0.01) | (0.03) |
| Transfers (Not Progressive) | 0.011 | 0.029 | 0.004 | -0.003 | 0.001 | 0.018 |
| | (0.02) | (0.01) | (0.00) | (0.01) | (0.03) | (0.01) |
| Combined (Progressive) | -0.011 | 0.001 | 0.014 | 0.027* | 0.003 | 0.014 |
| | (0.02) | (0.02) | (0.01) | (0.01) | (0.00) | (0.02) |
| Combined (Not Progressive) | 0.018** | -0.022* | 0.041 | 0.021 | -0.008 | 0.033 |
| | (0.00) | (0.00) | (0.01) | (0.03) | (0.01) | (0.01) |
| **Panel B - Respondents stated that they were neither an employee or self-employed** | | | | | | |
| Taxes (Progressive) | 0.005 | 0.032 | 0.032* | 0.011 | 0.011 | 0.041** |
| | (0.02) | (0.02) | (0.01) | (0.02) | (0.01) | (0.01) |
| Taxes (Not Progressive) | -0.005 | -0.002 | -0.009 | -0.041 | -0.001 | -0.024 |
| | (0.02) | (0.02) | (0.02) | (0.02) | (0.04) | (0.01) |
| Transfers (Progressive) | -0.010 | 0.019 | 0.019 | -0.025 | 0.006 | 0.005 |
| | (0.01) | (0.02) | (0.01) | (0.01) | (0.01) | (0.01) |
| Transfers (Not Progressive) | -0.014 | -0.019 | -0.001 | -0.000 | -0.020 | -0.021 |
| | (0.01) | (0.01) | (0.01) | (0.05) | (0.01) | (0.04) |
| Combined (Progressive) | 0.013 | 0.013 | 0.028** | 0.012 | 0.021 | 0.037* |
| | (0.02) | (0.01) | (0.01) | (0.01) | (0.01) | (0.01) |
| Combined (Not Progressive) | -0.047 | -0.025 | -0.023 | -0.006 | -0.041 | -0.062 |
| | (0.05) | (0.02) | (0.01) | (0.02) | (0.02) | (0.03) |

Note: This table shows the heterogeneous treatment effects based on whether respondents' were working or not, where countries are pooled based on whether the tax and/or transfer system is actually progressive. This table is based on Equation 7 in Section 3 of the paper, except the regression analysis is conducted separately for respondents based on whether or not they are working. Respondents' employment status is based on Q3. * $p < 0.1$, ** $p < 0.05$, *** $p < 0.01$. *Direct:* Based on Q14, which asks whether respondents would not pay tax if they knew they would not get caught (variable takes value of 0 if they select "Strongly Agree" or "Agree" and 1 otherwise). *Punishable:* Based on Q15, which asks respondents their views about people not paying tax (variable takes value of 1 if they select "This is wrong and punishable" and 0 otherwise). *Important:* Based on Q16, which asks respondents whether it is important for people to pay tax (variable takes value of 1 if they select "Strongly Agree" or "Agree" and 0 otherwise). *Right to Tax:* Based on Q17, which asks respondents whether the government always has a right to make people pay tax (variable takes value of 1 if they select "Strongly Agree" or "Agree" and 0 otherwise). *Do not Refuse:* Based on Q18, which asks whether people should refuse to pay taxes until they receive more government transfers (variable takes value of 1 if they select "Strongly Disagree" or "Disagree" and 1 otherwise). *INDEX:* An unweighted average of the Z-scores of all five outcome variables, oriented so that a higher index means higher tax morale.

Table A20: Differences in the impact of the treatments in countries where either taxes or transfers are not progressive

| | Direct<br>b/se/p | Punishable<br>b/se/p | Important<br>b/se/p | Right to Tax<br>b/se/p | Refuse to Pay<br>b/se/p | INDEX<br>b/se/p |
|---|---|---|---|---|---|---|
| Taxes (Not Progressive) × Transfers (Progressive) | -0.014 | -0.018 | -0.018 | -0.023 | -0.029 | -0.043** |
| | (0.01) | (0.01) | (0.01) | (0.01) | (0.02) | (0.01) |
| p-value | 0.286 | 0.203 | 0.270 | 0.182 | 0.259 | 0.015 |
| Observations | 10689 | 10689 | 10689 | 10689 | 10689 | 10689 |
| Taxes (Progressive) × Transfers (Not progressive) | 0.013 | 0.010 | 0.005 | 0.020 | 0.027 | 0.033** |
| | (0.01) | (0.00) | (0.00) | (0.01) | (0.02) | (0.00) |
| p-value | 0.264 | 0.126 | 0.409 | 0.269 | 0.361 | 0.043 |
| Observations | 5150 | 5150 | 5150 | 5150 | 5150 | 5150 |

Note:. This table shows the impact of tax treatment compared to the transfers and combined treatments in the six countries for which these treatments were in opposing directions (Ghana, Indonesia, Jordan, Sri Lanka, South Africa and Tanzania). This table is based on Equation 7 in Section 3 of the paper, except the treatment dummy is coded such that it takes on the value of 1 if the respondent received the taxes treatment and 0 if they respondent received either the transfers or combined treatment. * $p < 0.1$, ** $p < 0.05$, *** $p < 0.01$. *Direct:* Based on Q14, which asks whether respondents would not pay tax if they knew they would not get caught (variable takes value of 0 if they select "Strongly Agree" or "Agree" and 1 otherwise). *Punishable:* Based on Q15, which asks respondents their views about people not paying tax (variable takes value of 1 if they select "This is wrong and punishable" and 0 otherwise). *Important:* Based on Q16, which asks respondents whether it is important for people to pay tax (variable takes value of 1 if they select "Strongly Agree" or "Agree" and 0 otherwise). *Right to Tax:* Based on Q17, which asks respondents whether the government always has a right to make people pay tax (variable takes value of 1 if they select "Strongly Agree" or "Agree" and 0 otherwise). *Do not Refuse:* Based on Q18, which asks whether people should refuse to pay taxes until they receive more government transfers (variable takes value of 1 if they select "Strongly Disagree" or "Disagree" and 1 otherwise). *INDEX:* An unweighted average of the Z-scores of all five outcome variables, oriented so that a higher index means higher tax morale.

Table A21: Overall effects of the treatments (excluding the fastest 5% and slowest 5% of respondents based on the time taken to complete survey)

| | Direct | Punishable | Important | Right to Tax | Do not Refuse | INDEX |
|---|---|---|---|---|---|---|
| | b/se/p | b/se/p | b/se/p | b/se/p | b/se/p | b/se/p |
| Taxes (Progressive) | -0.001 | 0.020 | 0.015* | 0.021* | 0.012 | 0.027** |
| | (0.01) | (0.01) | (0.00) | (0.01) | (0.01) | (0.01) |
| p-value | 0.945 | 0.274 | 0.050 | 0.099 | 0.357 | 0.036 |
| Observations | 7045 | 7059 | 7062 | 7063 | 7048 | 7246 |
| Taxes (Not Progressive) | -0.031 | -0.028* | -0.005 | -0.017 | -0.034 | -0.043** |
| | (0.01) | (0.01) | (0.01) | (0.01) | (0.02) | (0.01) |
| p-value | 0.113 | 0.055 | 0.615 | 0.164 | 0.269 | 0.035 |
| Observations | 6737 | 6756 | 6762 | 6759 | 6717 | 6959 |
| Transfers (Progressive) | -0.016* | 0.006 | 0.012 | 0.008 | 0.001 | 0.006 |
| | (0.01) | (0.01) | (0.01) | (0.01) | (0.01) | (0.01) |
| p-value | 0.097 | 0.695 | 0.227 | 0.621 | 0.896 | 0.711 |
| Observations | 10222 | 10242 | 10252 | 10240 | 10219 | 10478 |
| Transfers (Not Progressive) | -0.006 | -0.001 | 0.008 | -0.000 | -0.008 | -0.008 |
| | (0.01) | (0.02) | (0.01) | (0.03) | (0.00) | (0.01) |
| p-value | 0.553 | 0.964 | 0.682 | 0.993 | 0.339 | 0.461 |
| Observations | 3646 | 3650 | 3651 | 3646 | 3631 | 3792 |
| Combined (Progressive) | -0.007 | -0.002 | 0.014* | 0.021 | 0.009 | 0.017 |
| | (0.01) | (0.01) | (0.01) | (0.01) | (0.01) | (0.01) |
| p-value | 0.634 | 0.885 | 0.056 | 0.110 | 0.166 | 0.270 |
| Observations | 9962 | 10001 | 10004 | 9996 | 9955 | 10245 |
| Combined (Not progressive) | -0.017 | -0.029 | 0.008** | 0.004 | -0.021 | -0.027 |
| | (0.02) | (0.02) | (0.00) | (0.02) | (0.01) | (0.01) |
| p-value | 0.559 | 0.319 | 0.021 | 0.883 | 0.238 | 0.314 |
| Observations | 3595 | 3593 | 3615 | 3598 | 3583 | 3735 |

Note: This table shows the overall impact of each of the treatments (excluding the fastest 5% and slowest 5% of respondents based on the time taken to complete survey) relative to the control group, where countries are pooled based on whether the tax and/or transfer system is progressive. This table is comparable to Table 1 in Section 4 of the paper. This table is based on Equation 7 in Section 3 of the paper. * $p < 0.1$, ** $p < 0.05$, *** $p < 0.01$. *Direct:* Based on Q14, which asks whether respondents would not pay tax if they knew they would not get caught (variable takes value of 0 if they select "Strongly Agree" or "Agree" and 1 otherwise). *Punishable:* Based on Q15, which asks respondents their views about people not paying tax (variable takes value of 1 if they select "This is wrong and punishable" and 0 otherwise). *Important:* Based on Q16, which asks respondents whether it is important for people to pay tax (variable takes value of 1 if they select "Strongly Agree" or "Agree" and 0 otherwise). *Right to Tax:* Based on Q17, which asks respondents whether the government always has a right to make people pay tax (variable takes value of 1 if they select "Strongly Agree" or "Agree" and 0 otherwise). *Do not Refuse:* Based on Q18, which asks whether people should refuse to pay taxes until they receive more government transfers (variable takes value of 1 if they select "Strongly disagree" or "Disagree" and 0 otherwise). *INDEX:* An unweighted average of the Z-scores of all five outcome variables, oriented so that a higher index means higher tax morale.

Table A22: Overall effects of the treatments (without controls)

| | Direct b/se/p | Punishable b/se/p | Important b/se/p | Right to Tax b/se/p | Do not Refuse b/se/p | INDEX b/se/p |
|---|---|---|---|---|---|---|
| Taxes (Progressive) | 0.010 | 0.023 | 0.026** | 0.016 | 0.014 | 0.039** |
| | (0.01) | (0.02) | (0.01) | (0.01) | (0.01) | (0.01) |
| p-value | 0.535 | 0.300 | 0.037 | 0.172 | 0.179 | 0.048 |
| Observations | 7605 | 7605 | 7605 | 7605 | 7605 | 7605 |
| Taxes (Not Progressive) | -0.022 | -0.022* | -0.013 | -0.027 | -0.029 | -0.048** |
| | (0.01) | (0.01) | (0.02) | (0.01) | (0.02) | (0.01) |
| p-value | 0.231 | 0.078 | 0.530 | 0.161 | 0.327 | 0.013 |
| Observations | 7435 | 7435 | 7435 | 7435 | 7435 | 7435 |
| Transfers (Progressive) | -0.006 | 0.010 | 0.016 | -0.002 | 0.005 | 0.010 |
| | (0.01) | (0.01) | (0.01) | (0.02) | (0.01) | (0.02) |
| p-value | 0.621 | 0.442 | 0.278 | 0.899 | 0.578 | 0.660 |
| Observations | 11318 | 11318 | 11318 | 11318 | 11318 | 11318 |
| Transfers (Not Progressive) | 0.001 | 0.005 | 0.003 | -0.005 | -0.009 | -0.000 |
| | (0.00) | (0.01) | (0.01) | (0.03) | (0.01) | (0.02) |
| p-value | 0.826 | 0.761 | 0.815 | 0.912 | 0.334 | 0.996 |
| Observations | 3810 | 3810 | 3810 | 3810 | 3810 | 3810 |
| Combined (Progressive) | 0.000 | 0.009 | 0.022* | 0.021* | 0.013** | 0.027* |
| | (0.01) | (0.01) | (0.01) | (0.01) | (0.00) | (0.01) |
| p-value | 0.987 | 0.394 | 0.059 | 0.095 | 0.040 | 0.080 |
| Observations | 11066 | 11066 | 11066 | 11066 | 11066 | 11066 |
| Combined (Not progressive) | -0.006 | -0.023 | 0.013 | 0.008 | -0.018 | -0.005 |
| | (0.02) | (0.01) | (0.01) | (0.03) | (0.02) | (0.02) |
| p-value | 0.855 | 0.252 | 0.271 | 0.829 | 0.478 | 0.861 |
| Observations | 3769 | 3769 | 3769 | 3769 | 3769 | 3769 |

Note: This table shows the overall impact of each of the treatments (without controls) relative to the control group, where countries are pooled based on whether the tax and/or transfer system is progressive. This table is comparable to Table 1 in Section 4 of the paper. This table is based on Equation 7 in Section 3 of the paper (except there are no control variables). * $p < 0.1$, ** $p < 0.05$, *** $p < 0.01$. *Direct:* Based on Q14, which asks whether respondents would not pay tax if they knew they would not get caught (variable takes value of 0 if they select "Strongly Agree" or "Agree" and 1 otherwise). *Punishable:* Based on Q15, which asks respondents their views about people not paying tax (variable takes value of 1 if they select "This is wrong and punishable" and 0 otherwise). *Important:* Based on Q16, which asks respondents whether it is important for people to pay tax (variable takes value of 1 if they select "Strongly Agree" or "Agree" and 0 otherwise). *Right to Tax:* Based on Q17, which asks respondents whether the government always has a right to make people pay tax (variable takes value of 1 if they select "Strongly Agree" or "Agree" and 0 otherwise). *Do not Refuse:* Based on Q18, which asks whether people should refuse to pay taxes until they receive more government transfers (variable takes value of 0 if they select "Strongly Agree" or "Agree" and 1 otherwise).

Table A23: Leebounds analysis for the taxes treatment

| | Direct<br>b/se/p | Punishable<br>b/se/p | Important<br>b/se/p | Right to Tax<br>b/se/p | Do not Refuse<br>b/se/p | INDEX<br>b/se/p |
|---|---|---|---|---|---|---|
| Taxes (Progressive) | | | | | | |
| Lower bound | -0.003 | 0.007 | 0.001 | -0.005 | -0.003 | 0.011 |
| | (0.01) | (0.01) | (0.01) | (0.01) | (0.01) | (0.02) |
| p-value | 0.792 | 0.519 | 0.905 | 0.677 | 0.774 | 0.506 |
| Upper bound | 0.013 | 0.016 | 0.010 | 0.011 | 0.009 | 0.030** |
| | (0.01) | (0.01) | (0.01) | (0.01) | (0.01) | (0.02) |
| p-value | 0.279 | 0.192 | 0.398 | 0.236 | 0.465 | 0.046 |
| Taxes (Not Progressive) | | | | | | |
| Lower bound | -0.028** | -0.018* | -0.018 | -0.007 | -0.026** | -0.055*** |
| | (0.01) | (0.01) | (0.01) | (0.01) | (0.01) | (0.02) |
| p-value | 0.022 | 0.098 | 0.145 | 0.587 | 0.017 | 0.000 |
| Upper bound | -0.016 | -0.005 | -0.007 | 0.002 | -0.013 | -0.017 |
| | (0.01) | (0.01) | (0.01) | (0.01) | (0.01) | (0.02) |
| p-value | 0.189 | 0.659 | 0.544 | 0.836 | 0.285 | 0.288 |

Note: This table presents the upper and lower Leebounds (based on Lee (2009)) for the taxes treatment, where countries are pooled based on whether the tax system is progressive. * $p < 0.1$, ** $p < 0.05$, *** $p < 0.01$. *Direct:* Based on Q14, which asks whether respondents would not pay tax if they knew they would not get caught (variable takes value of 0 if they select "Strongly Agree" or "Agree" and 1 otherwise). *Punishable:* Based on Q15, which asks respondents their views about people not paying tax (variable takes value of 1 if they select "This is wrong and punishable" and 0 otherwise). *Important:* Based on Q16, which asks respondents whether it is important for people to pay tax (variable takes value of 1 if they select "Strongly Agree" or "Agree" and 0 otherwise). *Right to Tax:* Based on Q17, which asks respondents whether the government always has a right to make people pay tax (variable takes value of 1 if they select "Strongly Agree" or "Agree" and 0 otherwise). *Do not Refuse:* Based on Q18, which asks whether people should refuse to pay taxes until they receive more government transfers (variable takes value of 1 if they select "Strongly disagree" or "Disagree" and 0 otherwise). *INDEX:* An unweighted average of the Z-scores of all five outcome variables, oriented so that a higher index means higher tax morale.

Table A24: Degree of Variation in Heterogeneous Treatment Effects

| Dimension | Countries where taxes are progressive | Countries where taxes are not progressive |
|---|---|---|
| Male | 0.13 | 0.09 |
| 18-34 years | 0.11 | 0.14 |
| Sec edu or less | 0.10 | 0.10 |
| Large city | 0.12 | 0.09 |
| Working | 0.12 | 0.13 |
| Believe B40 | 0.12 | 0.09 |
| Believe progressive | 0.10 | 0.27 |
| Prefer progressive | 0.20 | 0.09 |

Note: The output in this table is based on the causal forest function within the Generalized Random Forest R package. The degree of heterogeneity in effects from the taxes treatment are calculated for each dimension, and the relative degree of variation in heterogeneity for each dimension is placed on a scale from 0 to 1 (i.e. so that the total across all dimensions is equal to 1). This exercise is completed independently for countries that have progressive and do not have progressive tax systems. *Male:* Based on Q0, which asks respondents whether they are male or female (variable takes value of 1 if they select "Male" and 0 otherwise). *18-34 years:* Based on Q0, which also asks respondents their age (variable takes value of 1 if they select between 18-34 years and 0 if they select 35 or older, noting respondents under the age of 18 years were automatically excluded). *Sec edu or less:* Based on Q1, which asks whether respondents their level of education (variable takes value of 1 if they select "Primary or less" or "Secondary" and 0 otherwise). *Large city:* Based on Q2, which asks respondents about where they live (variable takes value of 1 if they select "Large city" and 0 otherwise). *Working:* Based on Q3, which asks whether respondents their current employment status (variable takes value of 1 if they select "Employee" or "Self employed" and 0 otherwise). *Believe B40:* Based on Q5, which asks respondents about their households place in the national income distribution (variable takes value of 1 if they select "Poorest group" or "Second poorest group" and 0 otherwise). Beliefs about progressivity are based on Q8, which asks respondents whether they believe that richer households pay a higher share of their income in tax than poorer households. Preferences about progressivity are based on Q9, which asks respondents whether they think that richer households should pay a higher share of their income in tax than poorer households.

Figure A1: Difference between the Gross and Net GINI index in all developing countries where comparable data exists

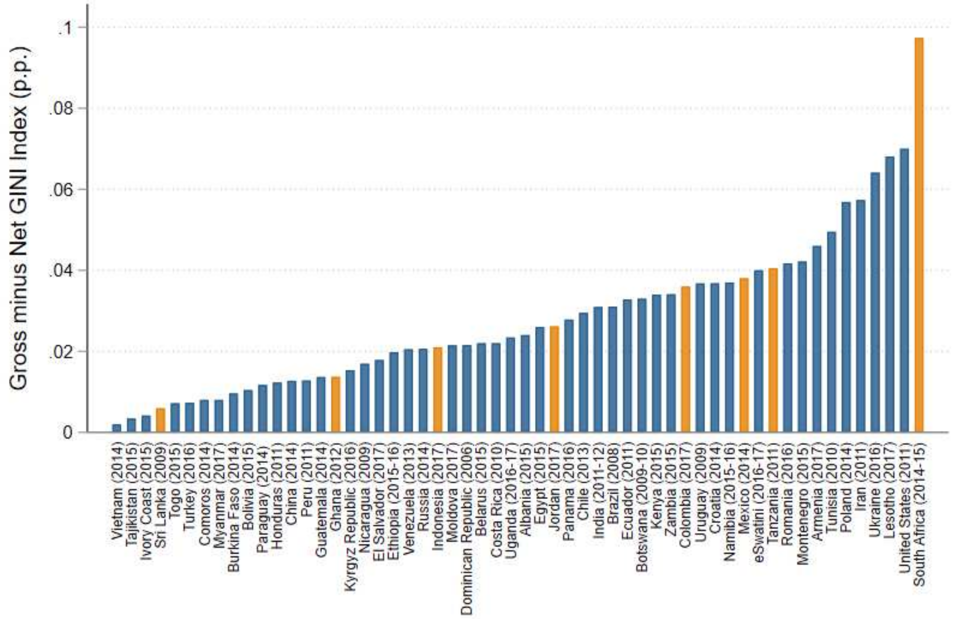


Note: This figure shows that the difference between the gross (i.e., pre-taxes and government transfers) and net (i.e., post-taxes and government transfers) GINI index is negligible in some countries in this study and far more substantial in others. Countries marked in yellow were included in this study. The year shown in brackets next to each country is the year in which the survey took place that the GINI index is based on. The United States is included as a point of comparison.

*Source: CEQ, 2021*

Figure A2: Share of respondents in each quintile in each country that agreed richer households should pay a higher share of their income in tax than poorer households

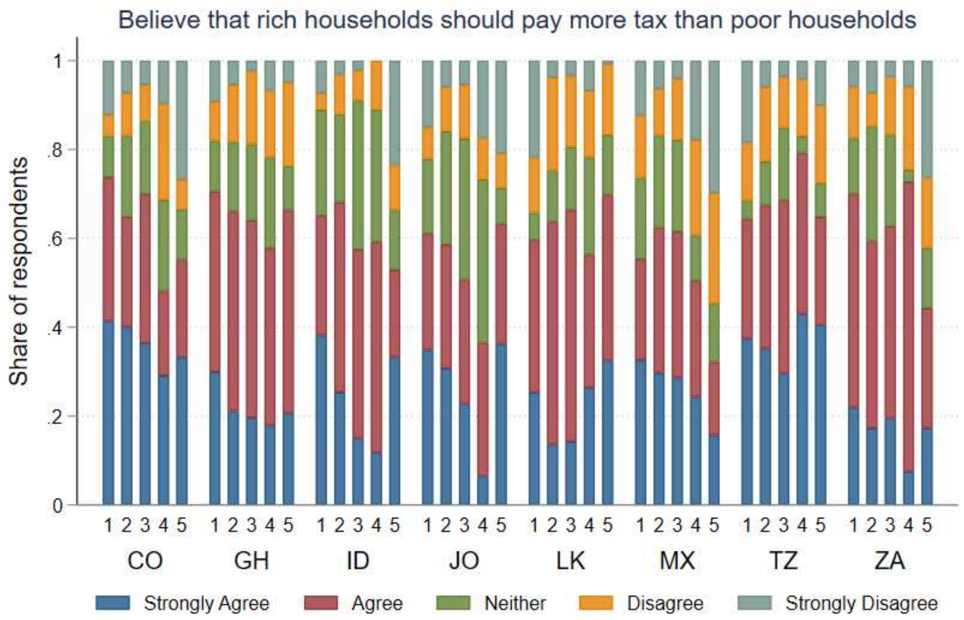


Note: This figure shows the share of respondents in each quintile in each country that agreed richer households should pay a higher share of their income in tax than poorer households. *CO:* Colombia. *GH:* Ghana. *ID:* Indonesia. *JO:* Jordan. *LK:* Sri Lanka. *MX:* Mexico. *TZ:* Tanzania. *ZA:* South Africa. Preferences about progressivity of taxes are based on Q9, which asks respondents whether they think that richer households should pay a higher share of their income in tax than poorer households.

Figure A3: Share of respondents in each quintile in each country that agreed poorer households should receive a higher share of their income in transfers than richer households

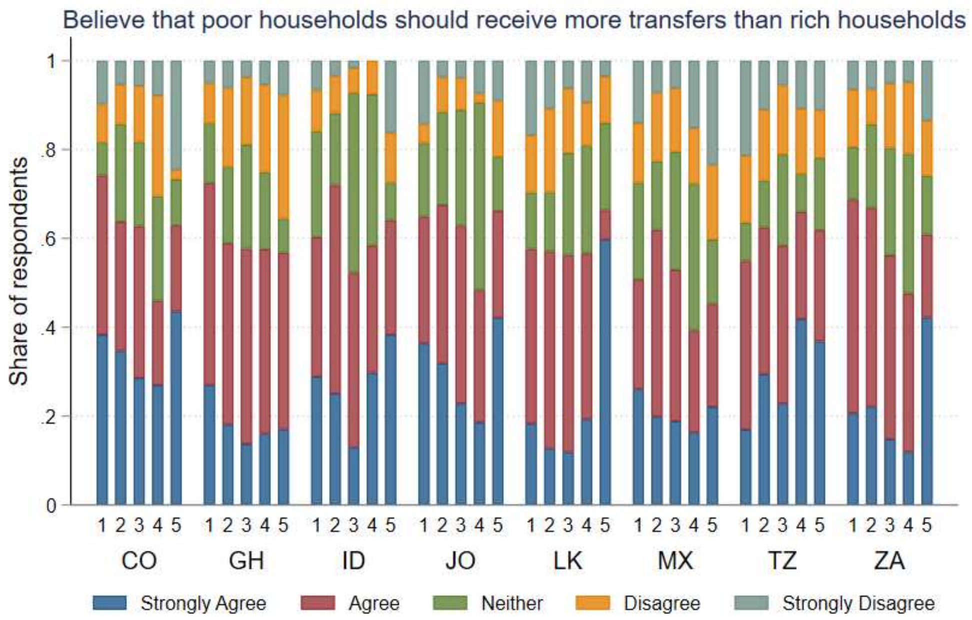


Note: This figure shows the share of respondents in each quintile in each country that agreed poorer households should receive a higher share of their income in transfers than richer households. *CO:* Colombia. *GH:* Ghana. *ID:* Indonesia. *JO:* Jordan. *LK:* Sri Lanka. *MX:* Mexico. *TZ:* Tanzania. *ZA:* South Africa. Preferences about progressivity of transfers are based on Q11, which asks respondents whether they think that poorer households should receive a higher share of their income in transfers than richer households.

Figure A4: Tax morale across countries according to different indicators

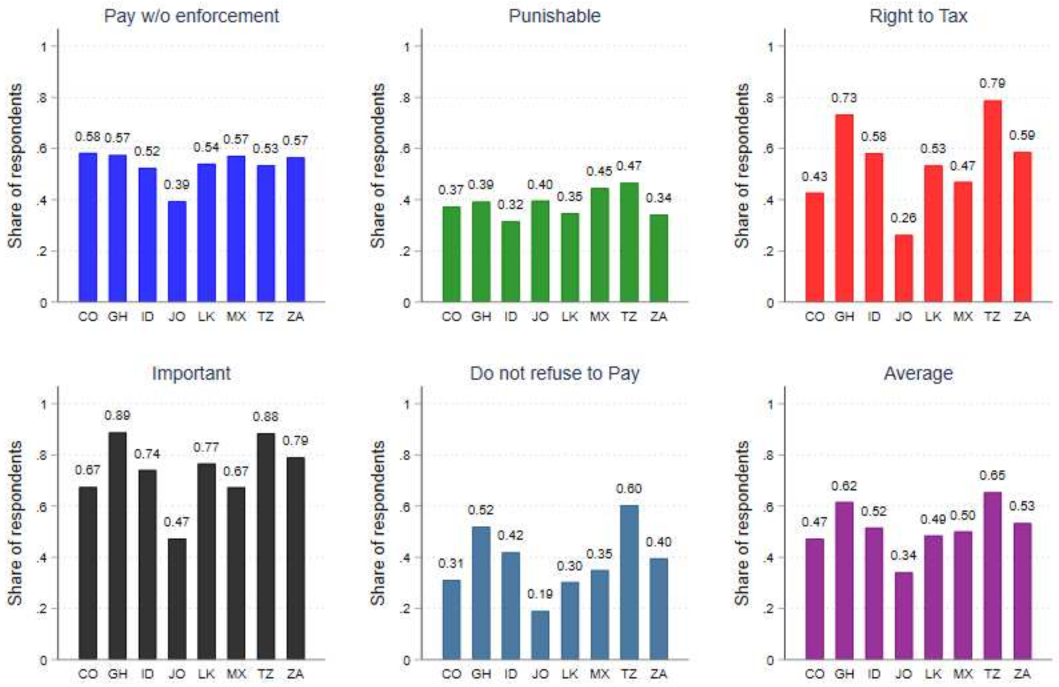


Note: This figure shows the share of respondents (in the control group) providing answers consistent with having higher tax morale in each country. *CO:* Colombia. *GH:* Ghana. *ID:* Indonesia. *JO:* Jordan. *LK:* Sri Lanka. *MX:* Mexico. *TZ:* Tanzania. *ZA:* South Africa. *Pay w/o enforcement:* Based on Q14, which asks whether respondents would not pay tax if they knew they would not get caught (variable takes value of 0 if they select "Strongly Agree" or "Agree" and 1 otherwise). *Punishable:* Based on Q15, which asks respondents their views about people not paying tax (variable takes value of 1 if they select "This is wrong and punishable" and 0 otherwise). *Important:* Based on Q16, which asks respondents whether it is important for people to pay tax (variable takes value of 1 if they select "Strongly Agree" or "Agree" and 0 otherwise). *Right to Tax:* Based on Q17, which asks respondents whether the government always has a right to make people pay tax (variable takes value of 1 if they select "Strongly Agree" or "Agree" and 0 otherwise). *Do not Refuse to Pay:* Based on Q18, which asks whether people should refuse to pay taxes until they receive more government transfers (variable takes value of 1 if they select "Strongly Disagree" or "Disagree" and 1 otherwise).

Figure A5a: Impact of the taxes and combined treatment on Tax morale by quintile

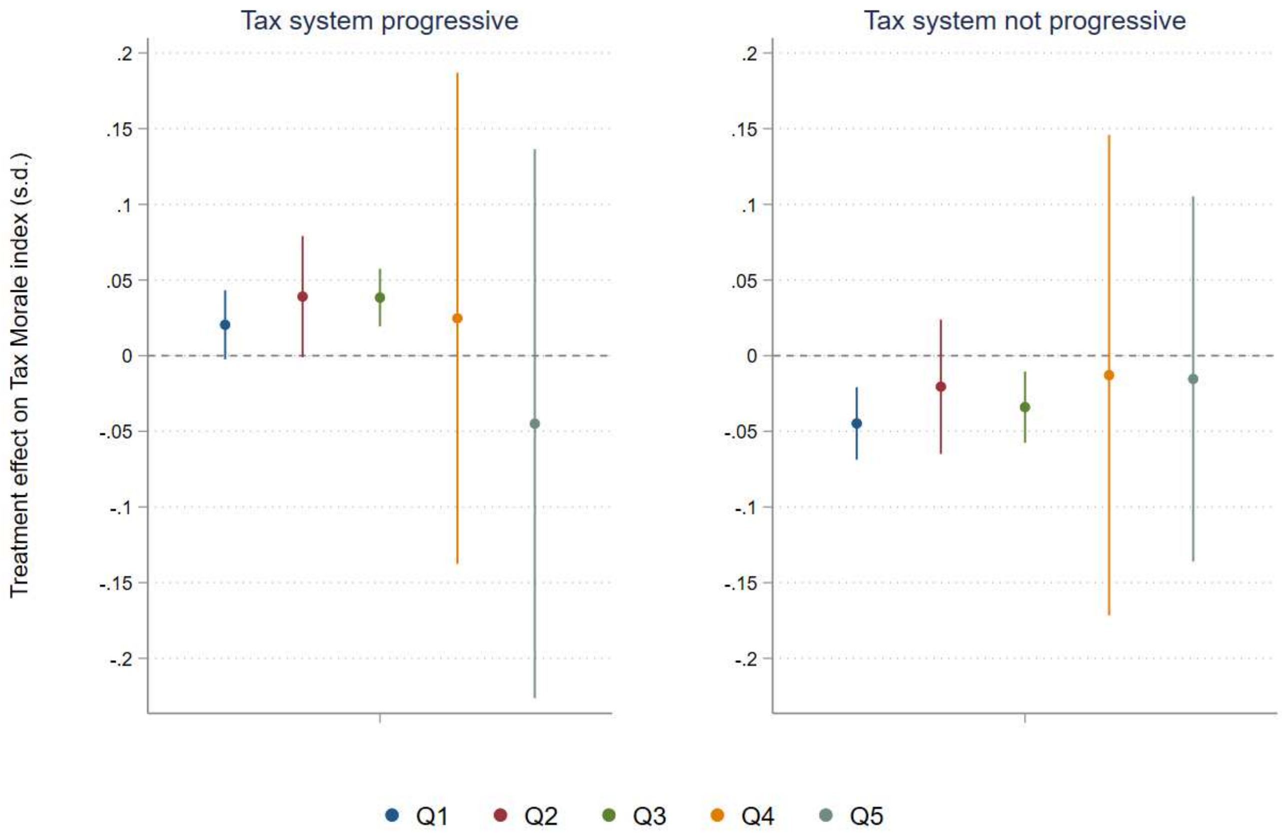

Figure A5b: Impact of the taxes and combined treatment on Tax morale by socio-economic status

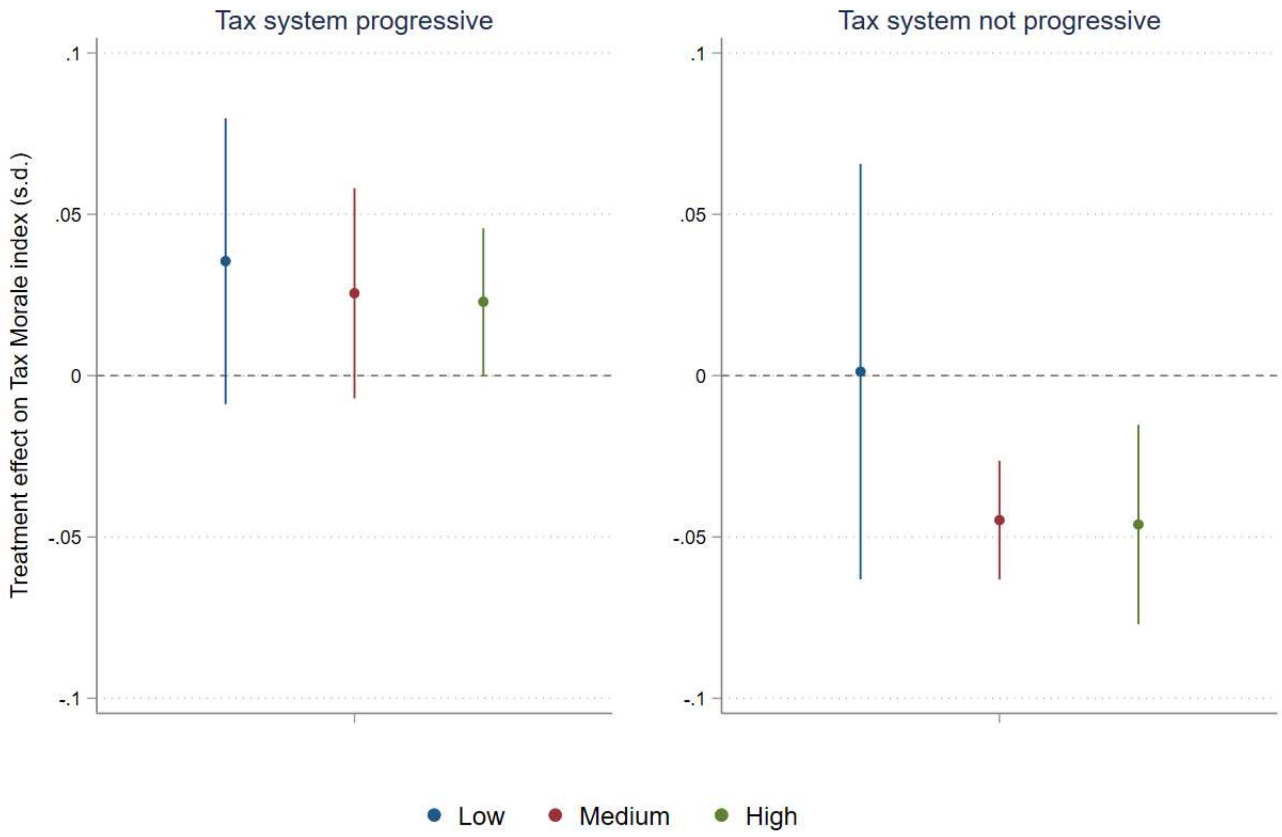

Figure A5c: Impact of the taxes and combined treatment on Tax morale by employment status

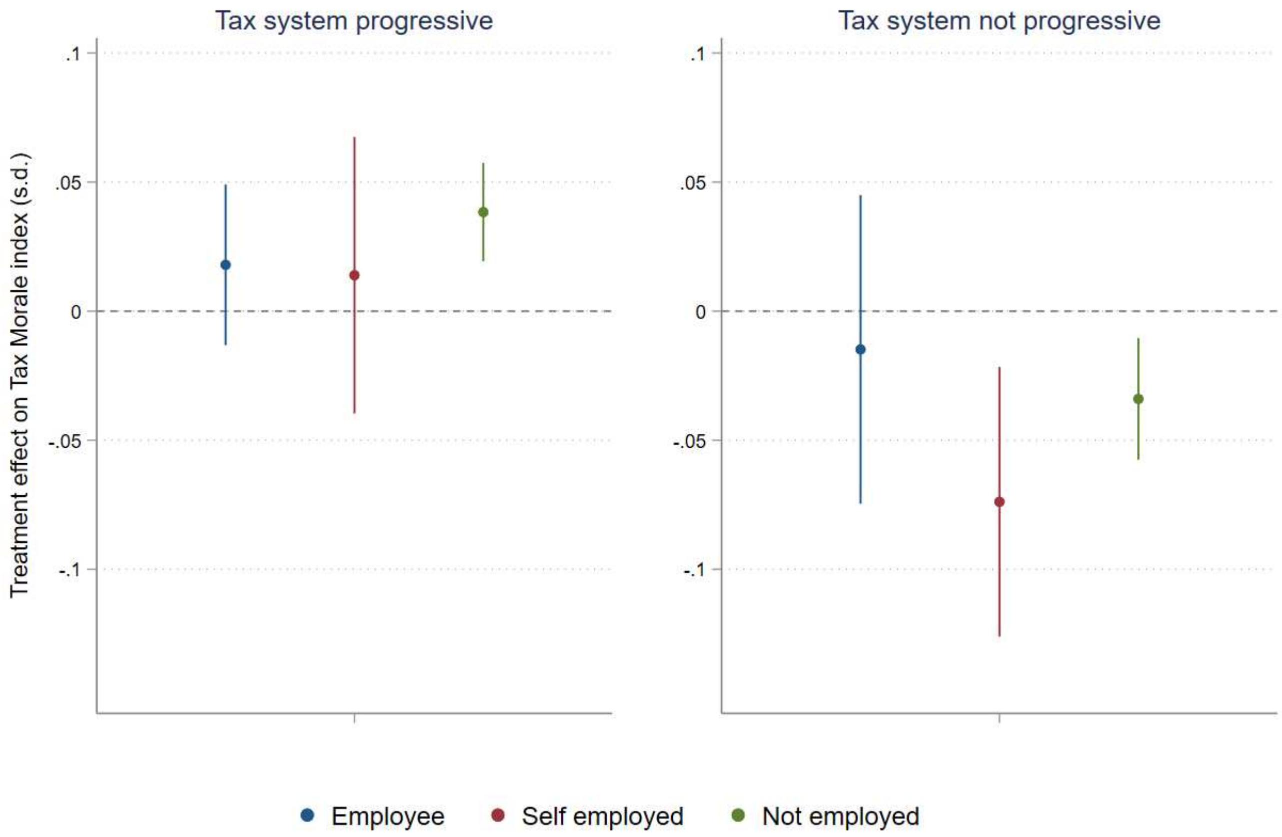

FIGURE A5D: Impact of the taxes and combined treatment on Tax morale by being a large taxpayer

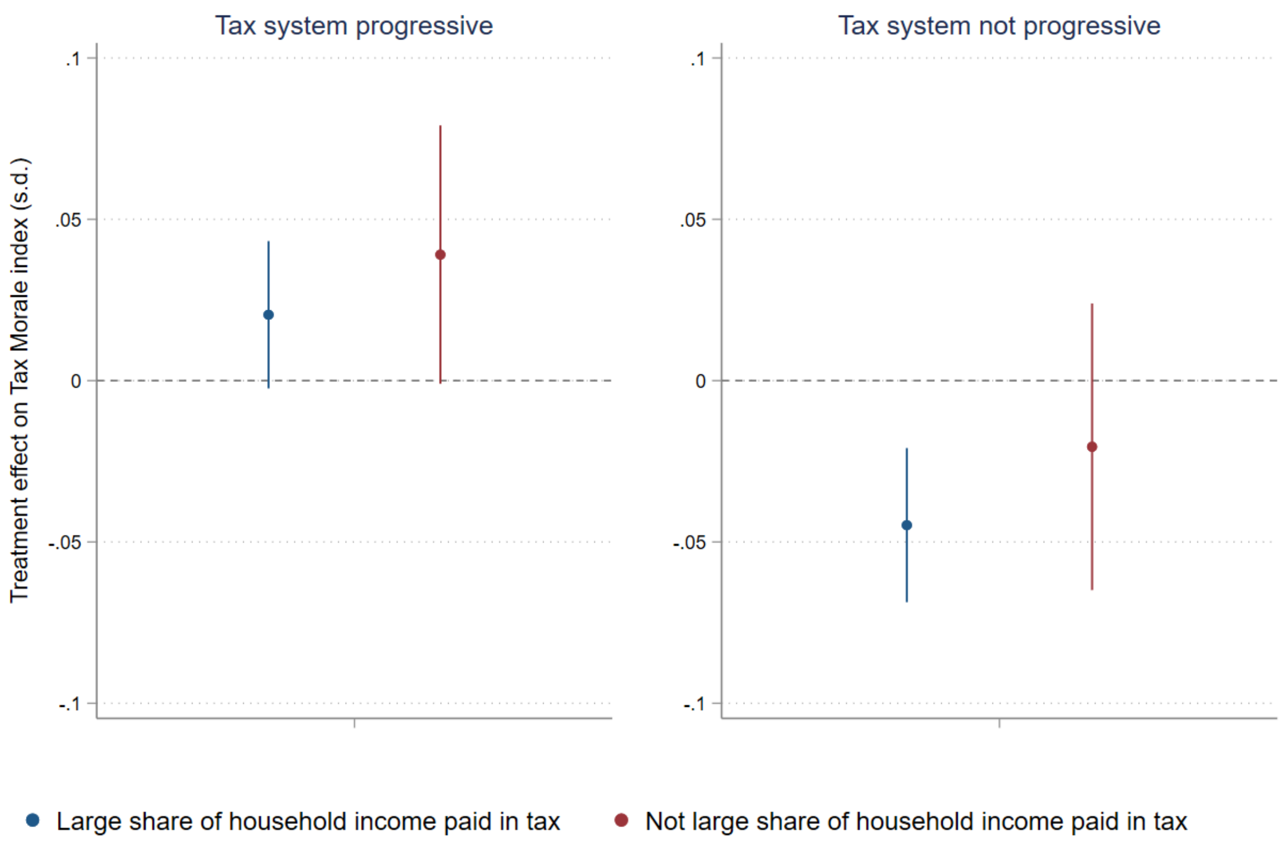

Figure A5e: Impact of the taxes and combined treatment on Tax morale by being a net contributor

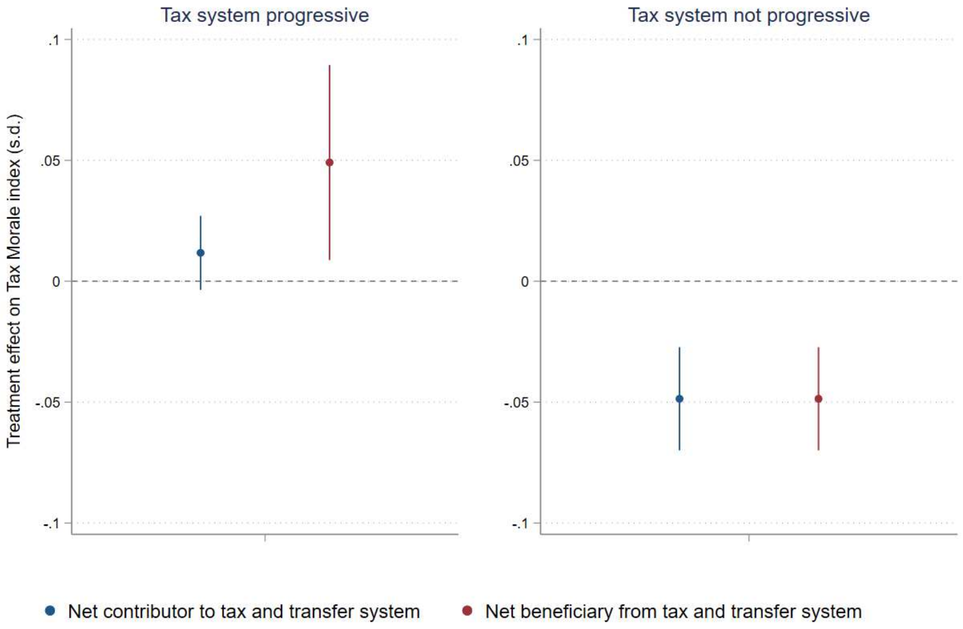


Note: Figure 6a shows the impact of the merged treatment (consisting of both the taxes and combined treatment groups) on the Tax morale index by quintile. Figure 6b shows the impact of the merged treatment (consisting of both the taxes and combined treatment groups) on the Tax morale index by socio-economic status (defined by location and education level). Figure 6c shows the impact of the merged treatment (consisting of both the taxes and combined treatment groups) on the Tax morale index by employment status. Figure 6d shows the impact of the merged treatment (consisting of both the taxes and combined treatment groups) on the Tax morale index by whether or not respondents are a large taxpayer. Figure 6e shows the impact of the merged treatment (consisting of both the taxes and combined treatment groups) on the Tax morale index by whether or not respondents are a net contributor to the tax and transfer system. *Q1:* Poorest quintile, based on answer to Q5. *Q2:* Second poorest quintile, based on answer to Q5. *Q3:* Middle quintile, based on answer to Q5. *Q4:* Second richest quintile, based on answer to Q5. *Q5:* Richest quintile, based on answer to Q5. 90 percent confidence intervals are displayed in this figure. Socio-economic status is based on respondents' education level and location (Q1 and Q2). Respondents' employment status is based on Q3. Beliefs about the share of household income that is paid in tax are based on Q6. Respondents' views about whether their household was a net contributor to the tax and transfer system is based on Q7. *INDEX:* An unweighted average of the Z-scores of all five outcome variables, oriented so that a higher index means higher Tax morale. 90 percent confidence intervals are displayed in this figure.

**Q0 (age_group / gender)**

# What is your age and gender?

You are invited to take part in this survey about your views on the tax and transfer system in your country. Please answer honestly and read the questions carefully. Your participation in this survey is voluntary and you may decline to take part or withdraw at any time. More information about the survey is available here https://tinyurl.com/a7bj5wks. Access to the data you provide will be restricted to the team of independent, non-partisan researchers and your responses will be entirely anonymous. If you would like to proceed, please click below.

## Male

| | | | | | | | | | |
|---|---|---|---|---|---|---|---|---|---|
| < 14 | | | | 14 | 15 | 16 | 17 | 18 | 19 |
| 20 | 21 | 22 | 23 | 24 | 25 | 26 | 27 | 28 | 29 |
| 30 | 31 | 32 | 33 | 34 | 35 | 36 | 37 | 38 | 39 |
| 40 | 41 | 42 | 43 | 44 | 45 | 46 | 47 | 48 | 49 |
| 50 | 51 | 52 | 53 | 54 | 55 | 56 | 57 | 58 | 59 |
| 60 | 61 | 62 | 63 | 64 | 65+ | | | | |

## Female

| | | | | | | | | | |
|---|---|---|---|---|---|---|---|---|---|
| < 14 | | | | 14 | 15 | 16 | 17 | 18 | 19 |
| 20 | 21 | 22 | 23 | 24 | 25 | 26 | 27 | 28 | 29 |
| 30 | 31 | 32 | 33 | 34 | 35 | 36 | 37 | 38 | 39 |
| 40 | 41 | 42 | 43 | 44 | 45 | 46 | 47 | 48 | 49 |
| 50 | 51 | 52 | 53 | 54 | 55 | 56 | 57 | 58 | 59 |
| 60 | 61 | 62 | 63 | 64 | 65+ | | | | |

Privacy Policy

Q1 (**q01_education)**

## What is the highest level of education you have completed?

- Primary or less
- Secondary
- Post-secondary vocational training
- Bachelor's degree
- Master's degree or higher

Q2 **(q02_live)**

## Where do you live?

- Large city
- Suburb
- Rural area / farm
- Small town / village

Q3 (**q03_employment)**

## What is your current employment status?

- Employee
- Self-employed or small business owner
- Not currently working and looking for work
- Not currently working and **not** looking for work
- Student

Q4 (**q04_rich_poor_gap)**

Do you agree or disagree with the following statement:

**The gap between the rich and the poor in [country] is too large**

| Strongly disagree | Disagree | Neither agree nor disagree | Agree | Strongly agree |
|---|---|---|---|---|

Q5 (**q05_house_income_group)**

Imagine the total population of [country] is divided into 5 income groups from poorest to richest, each with the same number of people.

**In which of these income groups do you place your household?**

| | | | | |
|---|---|---|---|---|
| **Poorest group** | **2nd Poorest group** | **Middle group** | **2nd Richest group** | **Richest group** |

Q6 (**q06_perceived_payed_tax)**

Do you agree or disagree with the following statement:

**In the last 12 months, my household has paid a large share of our income in taxes* to the government**

**Taxes decrease household income either directly (e.g., taxes on wages) or indirectly by increasing the prices of goods and services (e.g., taxes on imported goods).*

| Strongly disagree | Disagree | Neither agree nor disagree | Agree | Strongly agree |
| --- | --- | --- | --- | --- |

Q7 (**q07_perceived_tax_and_transfer)**

×

Do you agree or disagree with the following statement:

**In the last 12 months, my household paid more in taxes than was received in government transfers***

**Government transfers increase household income either directly (e.g., an elderly pension) or indirectly by decreasing the prices of goods and services (e.g., a fuel subsidy).*

| | | | | |
|---|---|---|---|---|
| Strongly disagree | Disagree | Neither agree nor disagree | Agree | Strongly agree |

Q8 (**q08_rich_pay_more_tax_now)**

Do you agree or disagree with the following statement:

**Currently, richer households in [country] pay a higher share of their income in tax than poorer households**

| Strongly disagree | Disagree | Neither agree nor disagree | Agree | Strongly agree |
|---|---|---|---|---|

Q9 (**q09_rich_should_pay_more_tax)**

Do you agree or disagree with the following statement:

**Richer households in [country] SHOULD pay a higher share of their income in tax than poorer households**

| Strongly disagree | Disagree | Neither agree nor disagree | Agree | Strongly agree |
|---|---|---|---|---|

Q10 (**q10_poor_incr_transfers_now)**

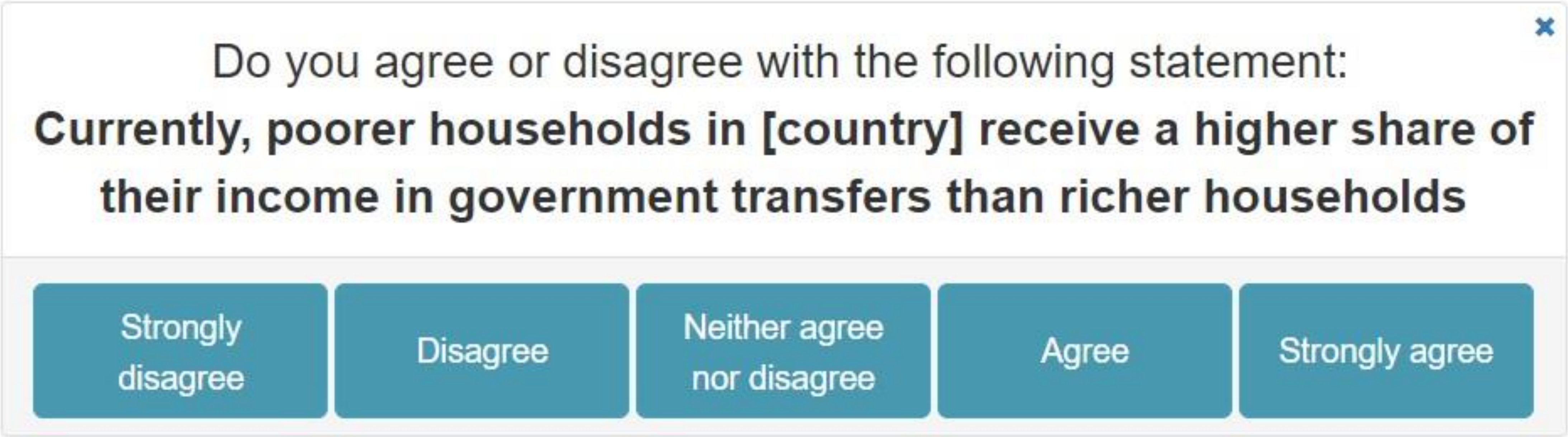

Q11 (**q11_poor_should_incr_transfers)**

Do you agree or disagree with the following statement:

**Poorer households in [country] SHOULD receive a higher share of their income in government transfers than richer households**

| Strongly disagree | Disagree | Neither agree nor disagree | Agree | Strongly agree |
|---|---|---|---|---|

Q12 (**q12_continue_with_survey)**

Before proceeding, please confirm that you are willing to answer the final 5 questions of the survey. If you cannot answer the next 5 questions, please indicate so below.

- Yes, I can answer another 5 questions
- No, I don't have time to answer another 5 questions

# Country Specific Experiments (Q13)

Q14 (**q14_wouldnt_pay_if_not_caught)**

Do you agree or disagree with the following statement:

**If I was sure I would not get caught, I would not pay all the taxes that I owe.**

| | | | | |
|---|---|---|---|---|
| Strongly disagree | Disagree | Neither agree nor disagree | Agree | Strongly agree |

Q15 (**q15_not_paying_taxes)**

Which best describes your thoughts on the following action:

**People not paying the taxes they owe to the government**

This is wrong and punishable

This is wrong but understandable

This is not wrong at all

Q16 (**q16_important_to_pay_tax)**

Do you agree or disagree with the following statement:

**It is important for people to pay taxes**

| | | | | |
|---|---|---|---|---|
| Strongly disagree | Disagree | Neither agree nor disagree | Agree | Strongly agree |

Q17 (**q17_gov_right_to_make_ppl_pay)**

Do you agree or disagree with the following statement:

**The government always has the right to make people pay taxes.**

| Strongly disagree | Disagree | Neither agree nor disagree | Agree | Strongly agree |
| --- | --- | --- | --- | --- |

Q18 (**q18_refuse_taxes)**

Do you agree or disagree with the following statement:

**People should refuse to pay taxes until they receive a larger share of household income in government transfers**

Strongly disagree | Disagree | Neither agree nor disagree | Agree | Strongly agree

Q19 (**q19_feedback)**

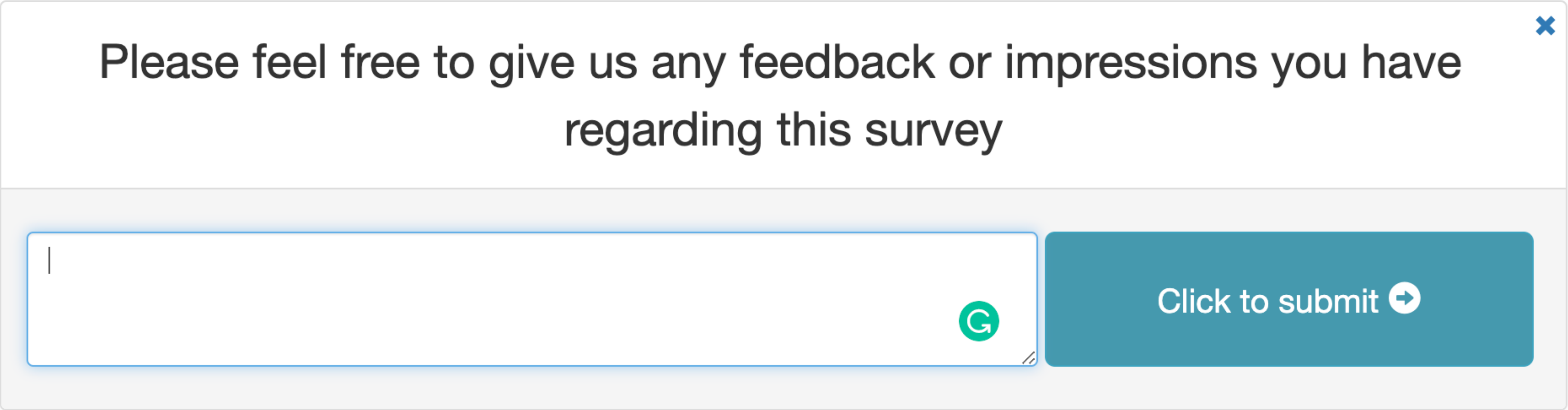

**Taxes treatment - Colombia**

# *Recent research* in Colombia shows:* Richer households pay a larger share of their income in taxes than Poorer households

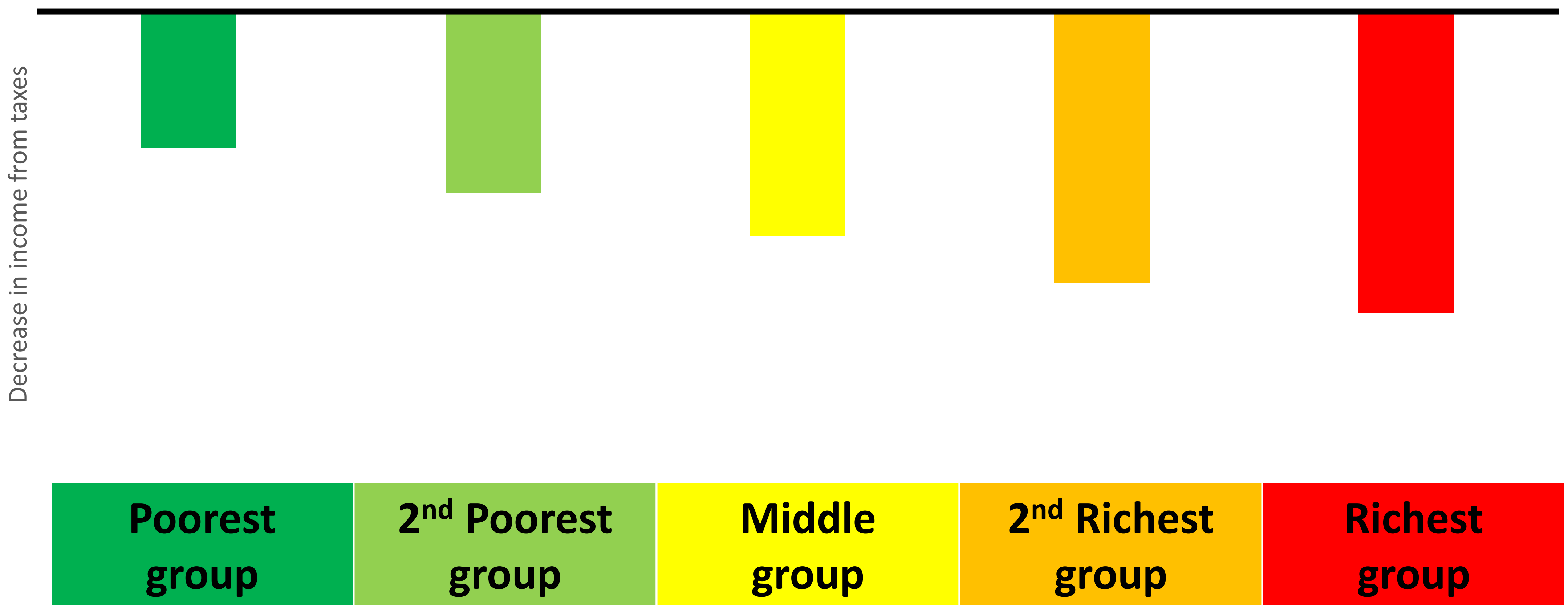


*This information recently became publicly available online through a collaboration between universities, civil society and international organisations.

**Transfers treatment - Colombia**

*Recent research* in Colombia shows:* Poorer households receive a much larger share of their income in government transfers than Richer households

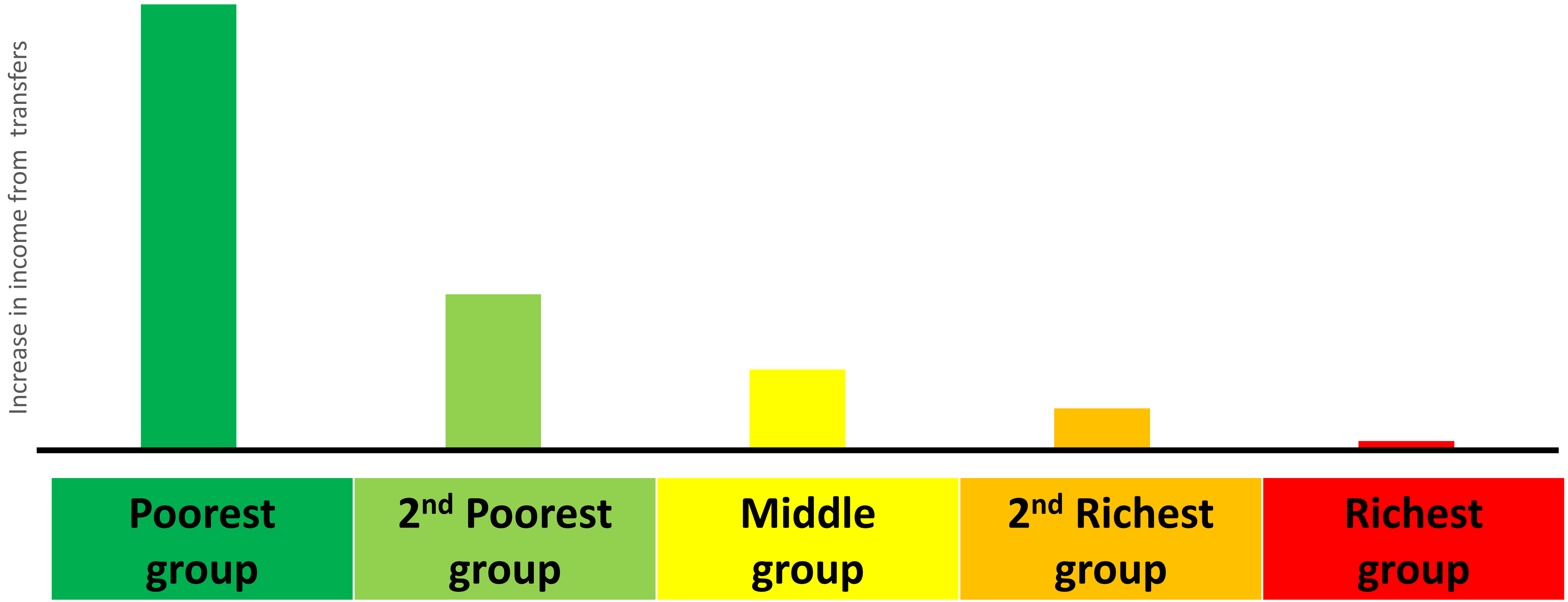


*This information recently became publicly available online through a collaboration between universities, civil society and international organisations.

**Taxes and transfers treatment - Colombia**

*Recent research* in Colombia shows:* Richer households pay more in taxes than they receive in government transfers, whereas Poorer households receive more in government transfers than they pay in taxes

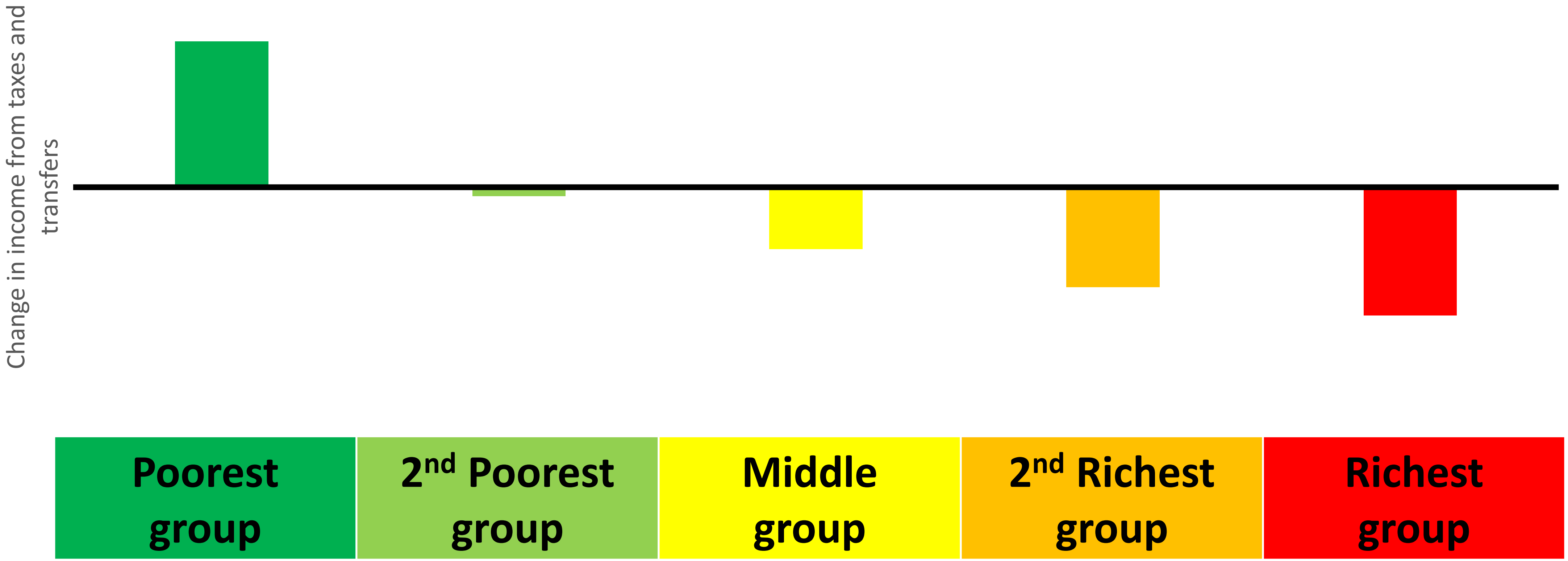


*This information recently became publicly available online through a collaboration between universities, civil society and international organisations.

**Taxes treatment - Ghana**

# *Recent research* in Ghana shows:* Richer households pay a larger share of their income in taxes than Poorer households

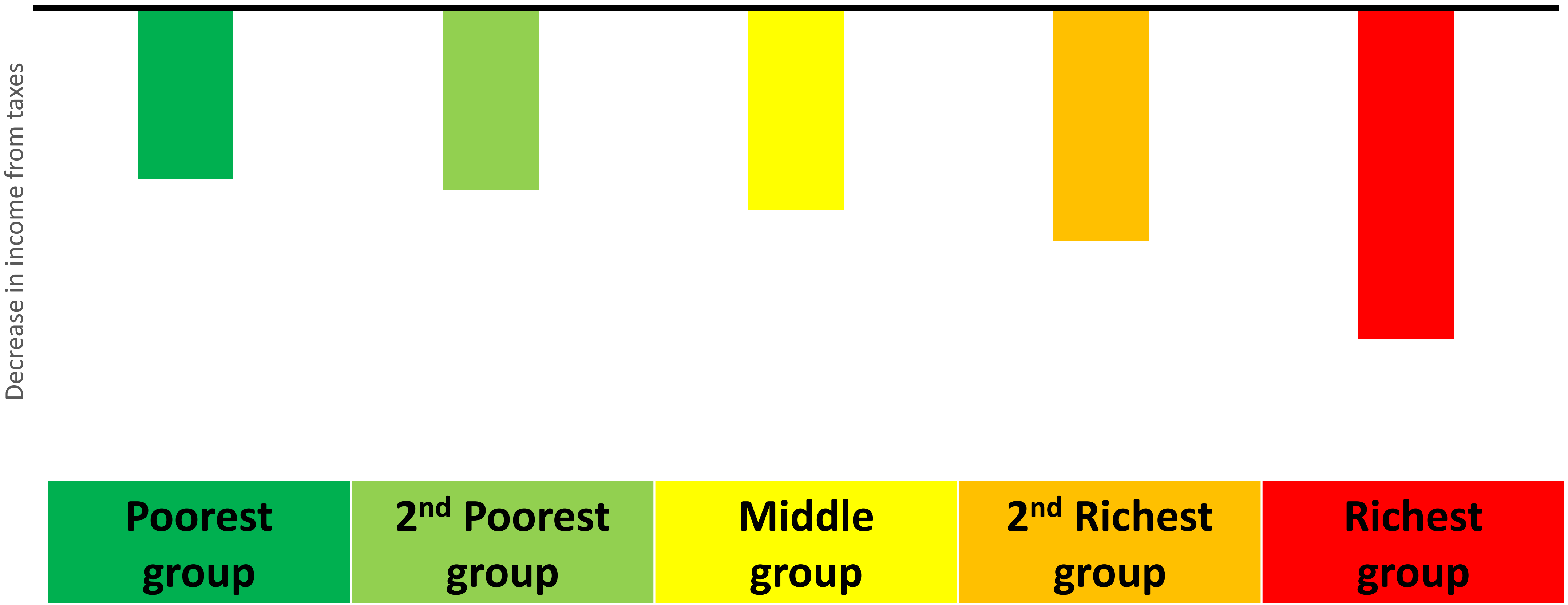


*This information recently became publicly available online through a collaboration between universities, civil society and international organisations.

**Transfers treatment - Ghana**

# *Recent research* in Ghana shows:* Poorer households receive a similar share of their income in government transfers as Richer households

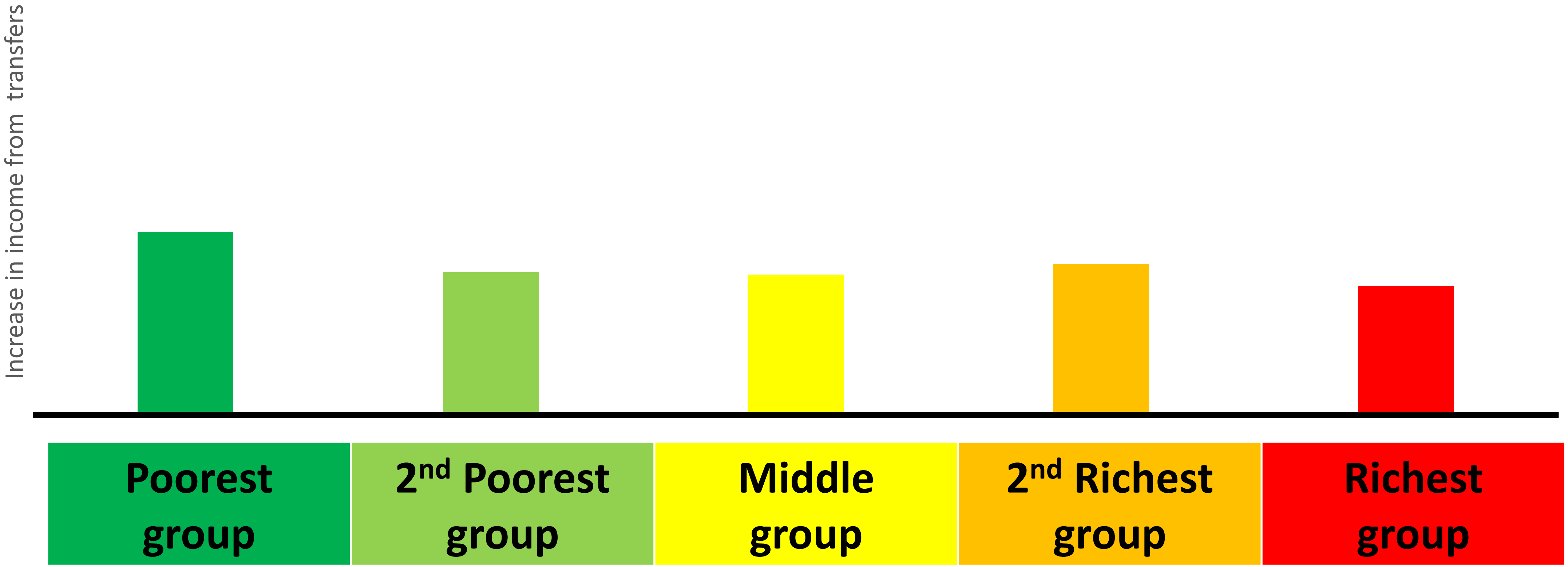


*This information recently became publicly available online through a collaboration between universities, civil society and international organisations.

**Taxes and transfers treatment - Ghana**

# *Recent research* in Ghana shows:* Most households pay more in taxes than they receive in government transfers and Richer households pay more than Poorer households

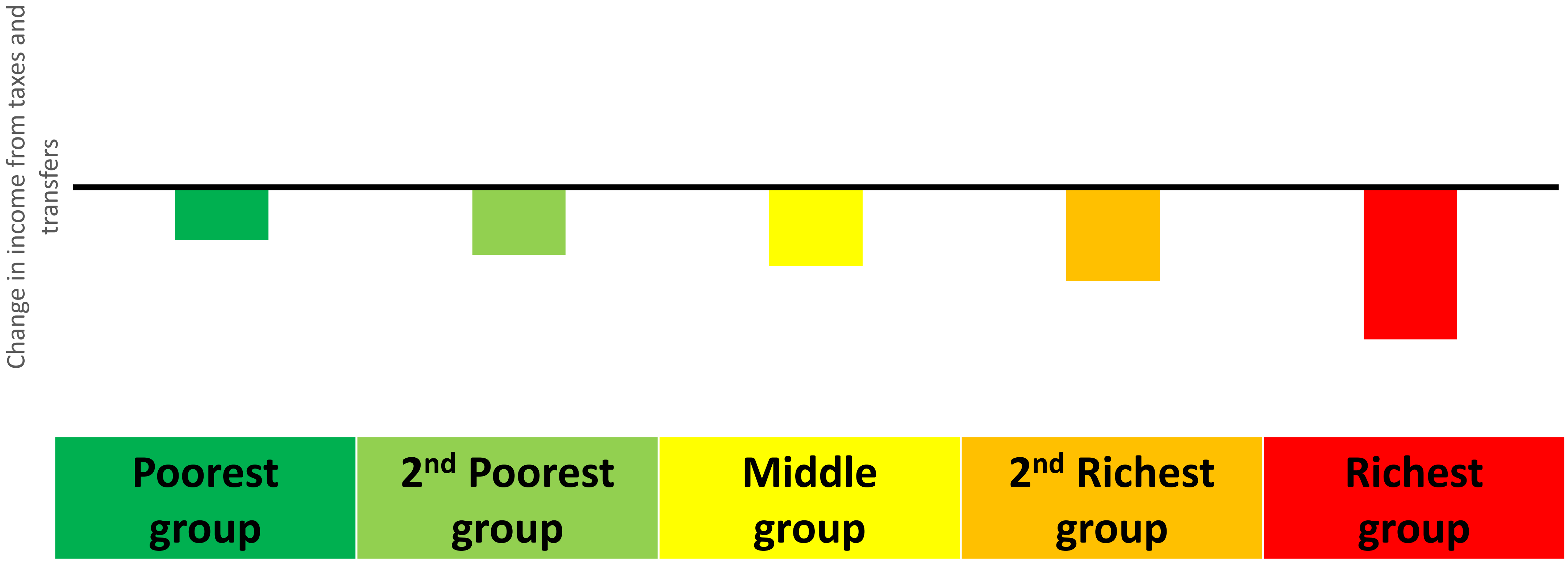


*This information recently became publicly available online through a collaboration between universities, civil society and international organisations.

# *Recent research* in Indonesia shows:* Richer households pay a similar share of their income in taxes as Poorer households

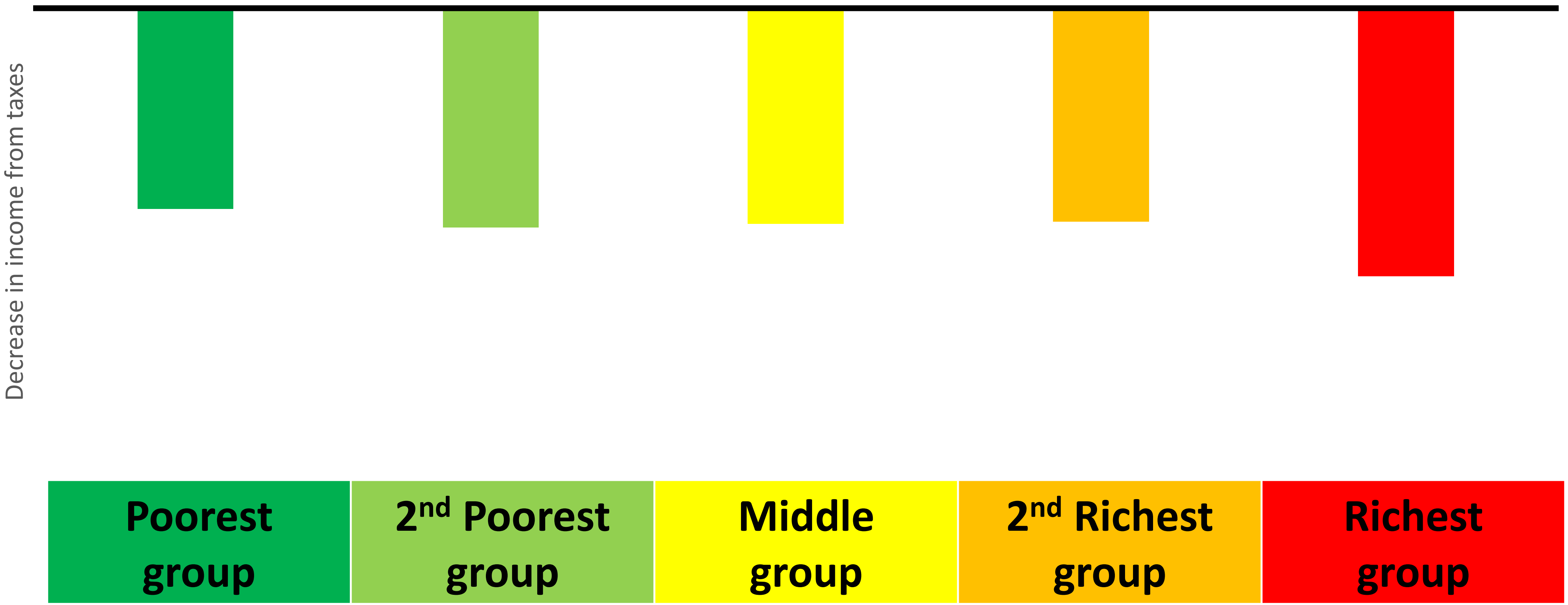


*This information recently became publicly available online through a collaboration between universities, civil society and international organisations.

**Transfers treatment - Indonesia**

# *Recent research* in Indonesia shows:* Poorer households receive a larger share of their income in government transfers than Richer households

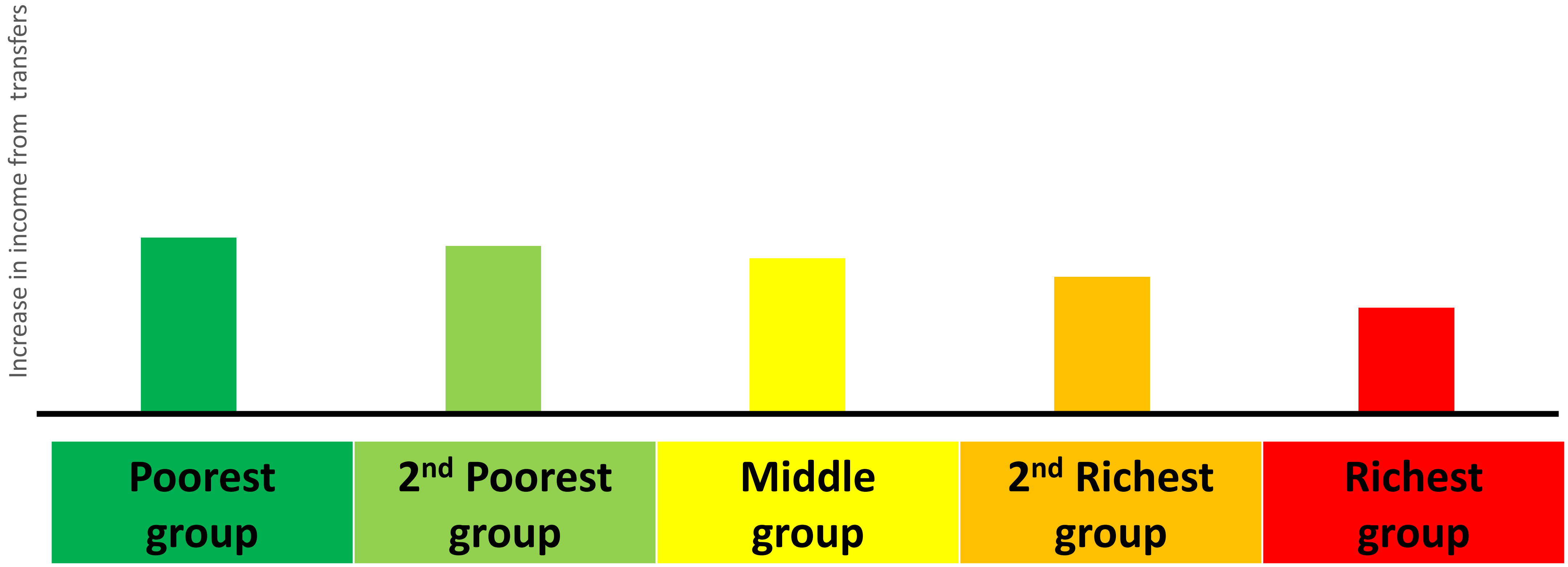


*This information recently became publicly available online through a collaboration between universities, civil society and international organisations.

**Taxes and transfers treatment - Indonesia**

*Recent research* in Indonesia shows:* Richer households pay more in taxes than they receive in government transfers, whereas Poorer households receive more in government transfers than they pay in taxes

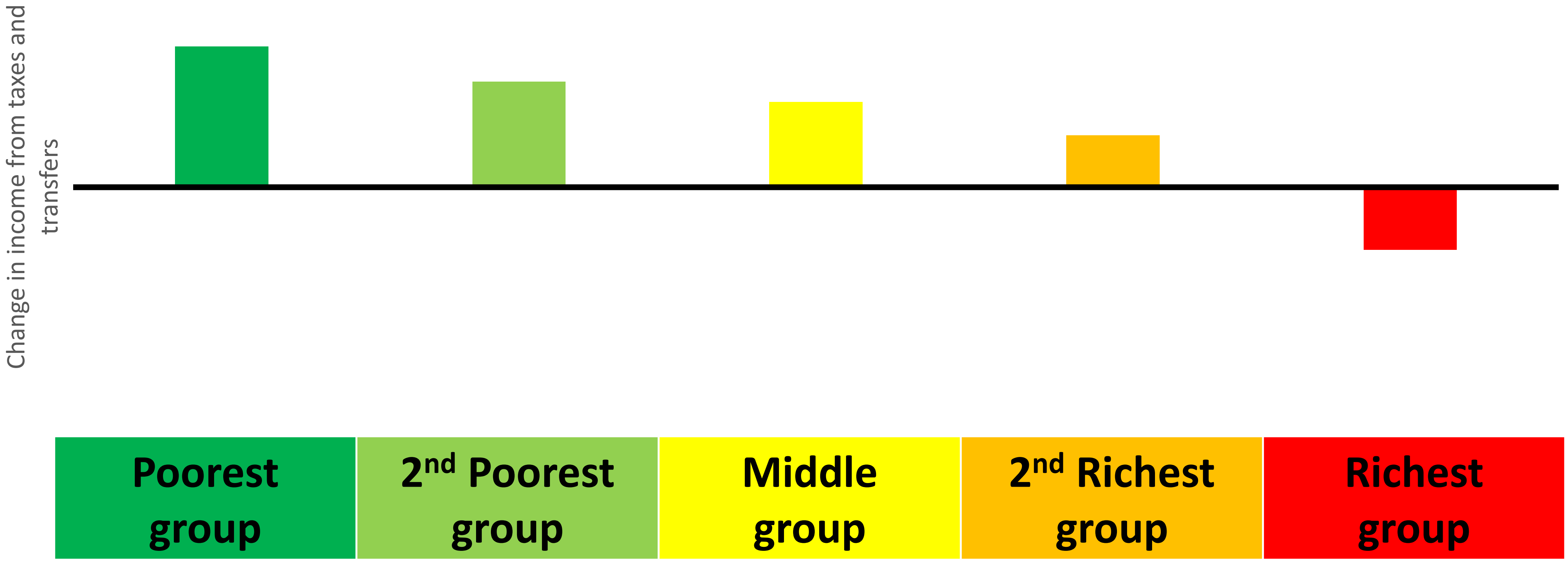


*This information recently became publicly available online through a collaboration between universities, civil society and international organisations.

# *Recent research* in Jordan shows:* Richer households pay a similar share of their income in taxes as Poorer households

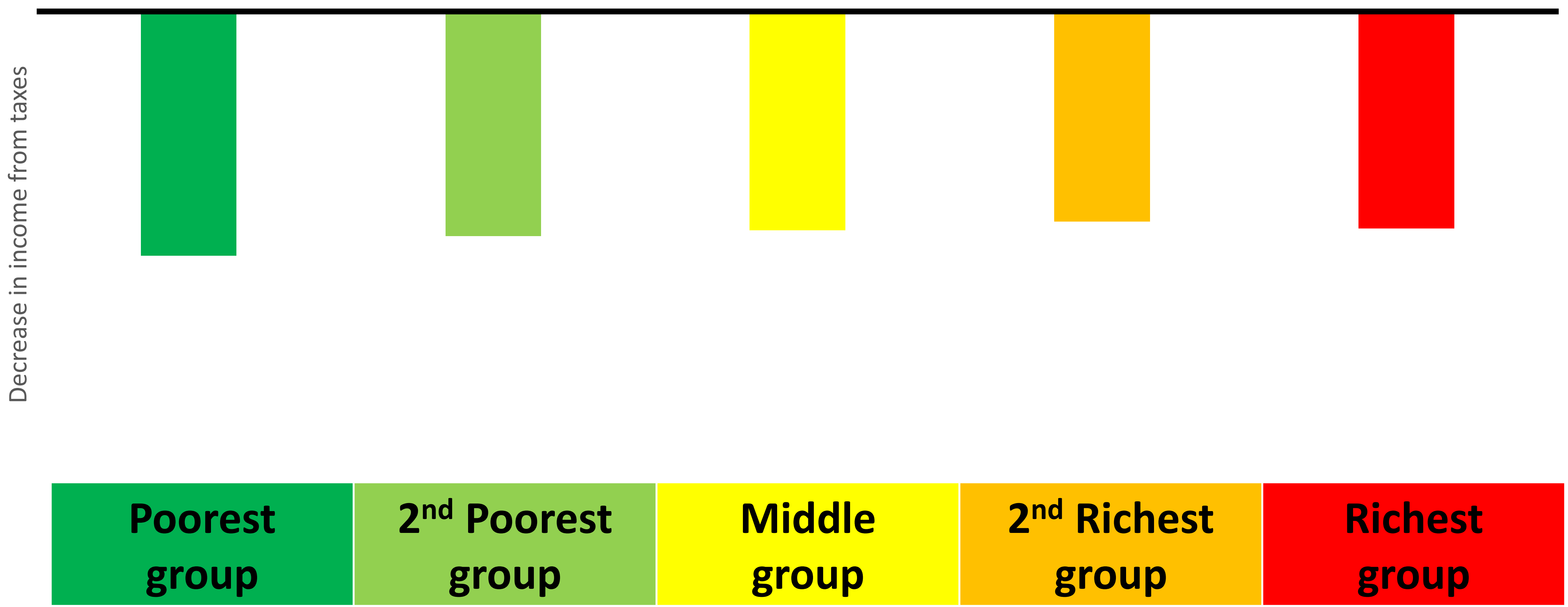


*This information recently became publicly available online through a collaboration between universities, civil society and international organisations.

Transfers treatment - Jordan

*Recent research* in Jordan shows:* Poorer households receive a much larger share of their income in government transfers than Richer households

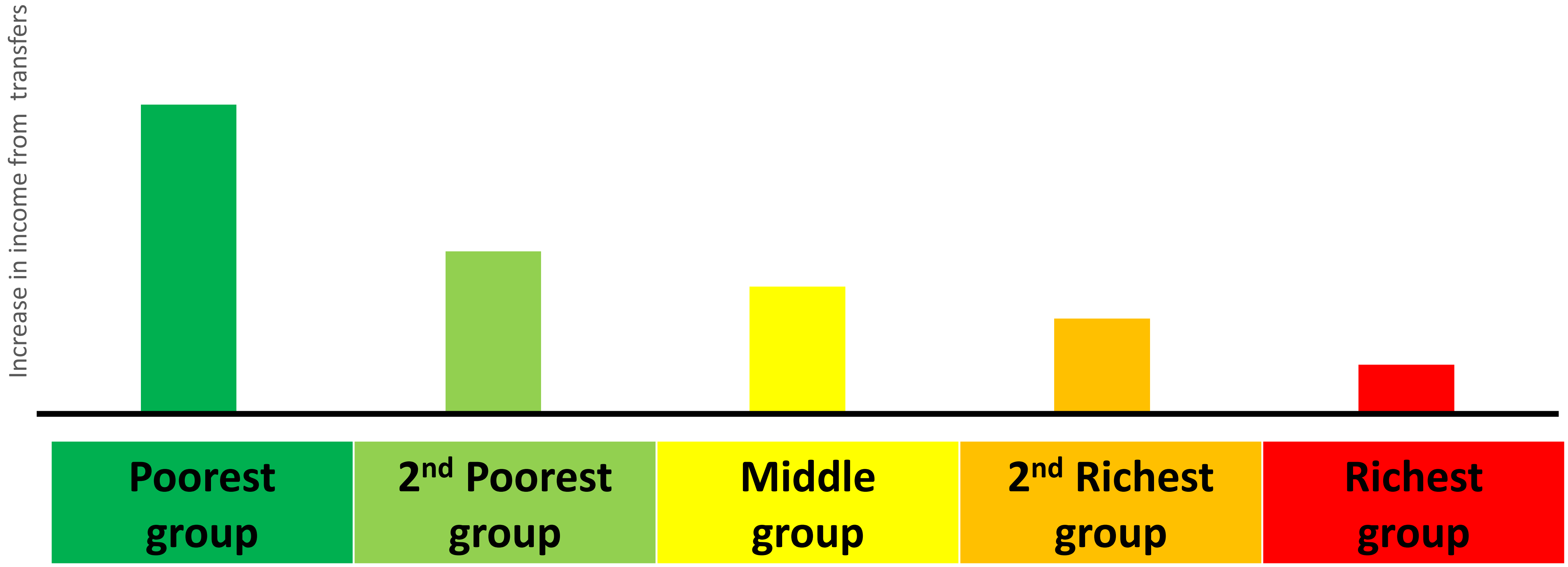


*This information recently became publicly available online through a collaboration between universities, civil society and international organisations.

**Taxes and transfers treatment - Jordan**

*Recent research* in Jordan shows:* Richer households pay more in taxes than they receive in government transfers, whereas Poorer households receive more in government transfers than they pay in taxes

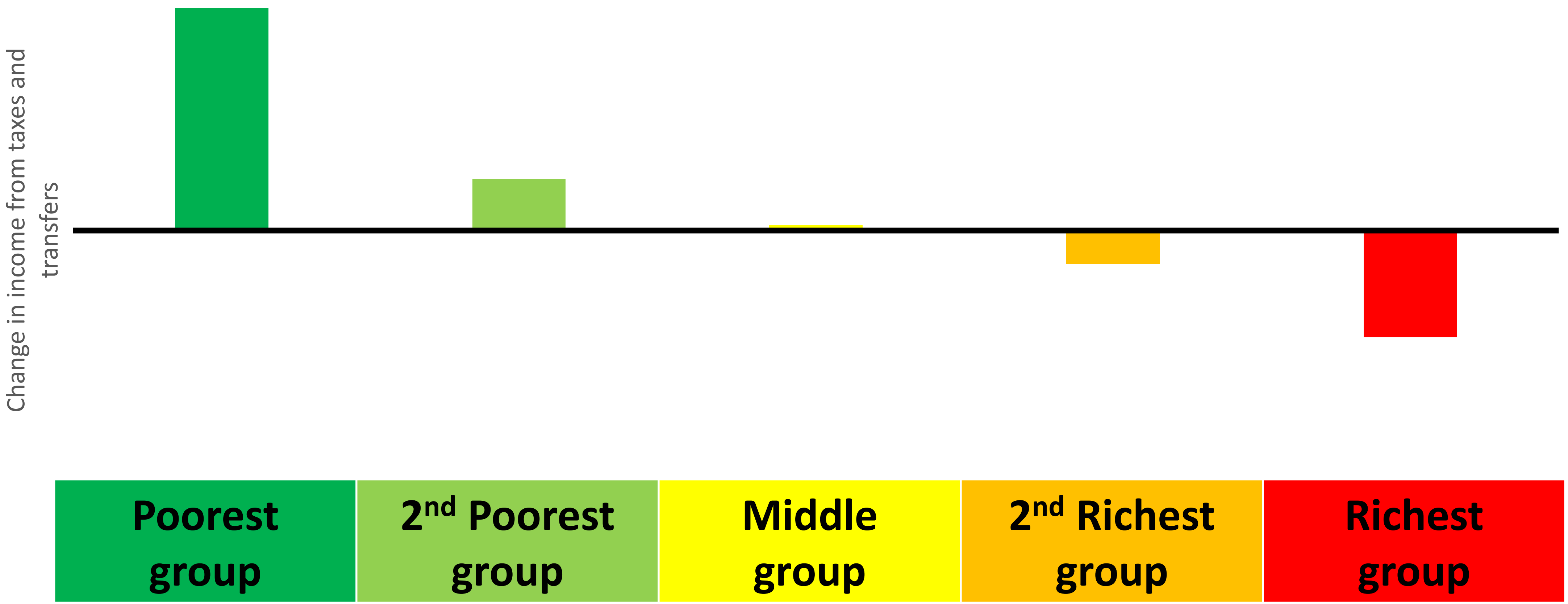


*This information recently became publicly available online through a collaboration between universities, civil society and international organisations.

**Taxes treatment - Mexico**

*Recent research* in Mexico shows:* Richer households pay a larger share of their income in taxes than Poorer households

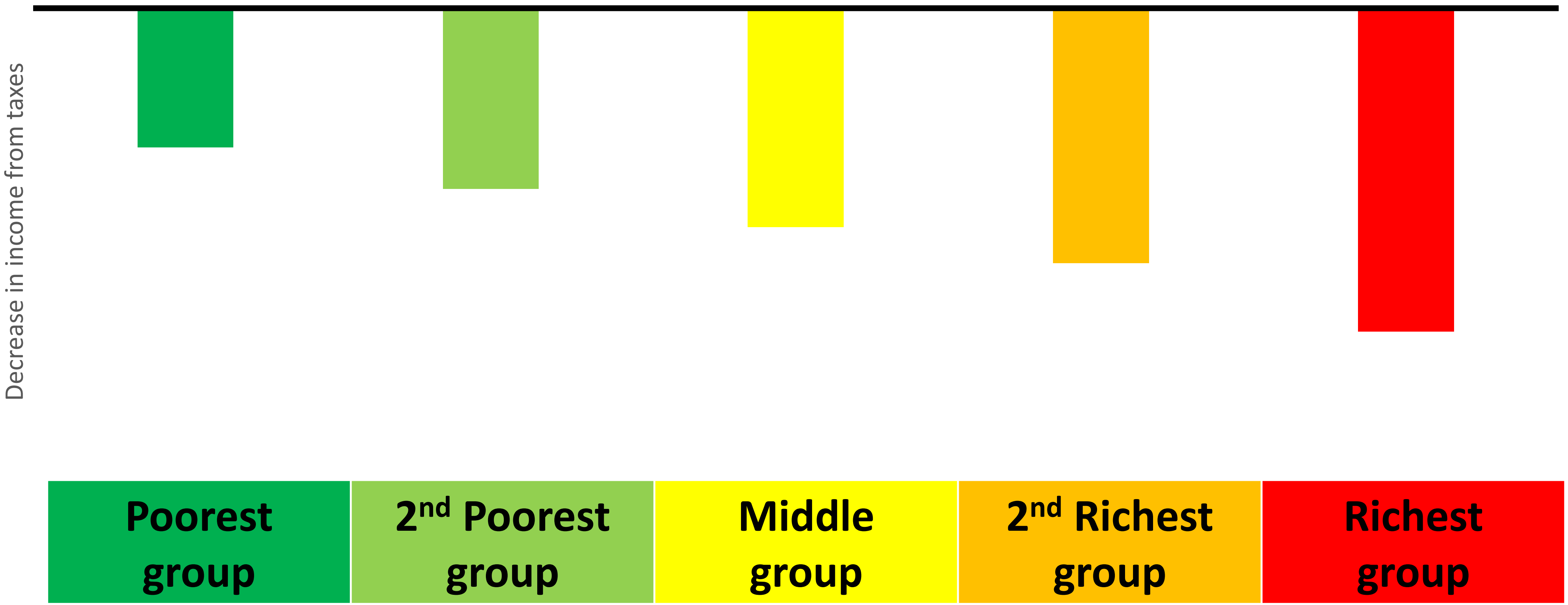


*This information recently became publicly available online through a collaboration between universities, civil society and international organisations.

**Transfers treatment - Mexico**

*Recent research* in Mexico shows:* Poorer households receive a much larger share of their income in government transfers than Richer households

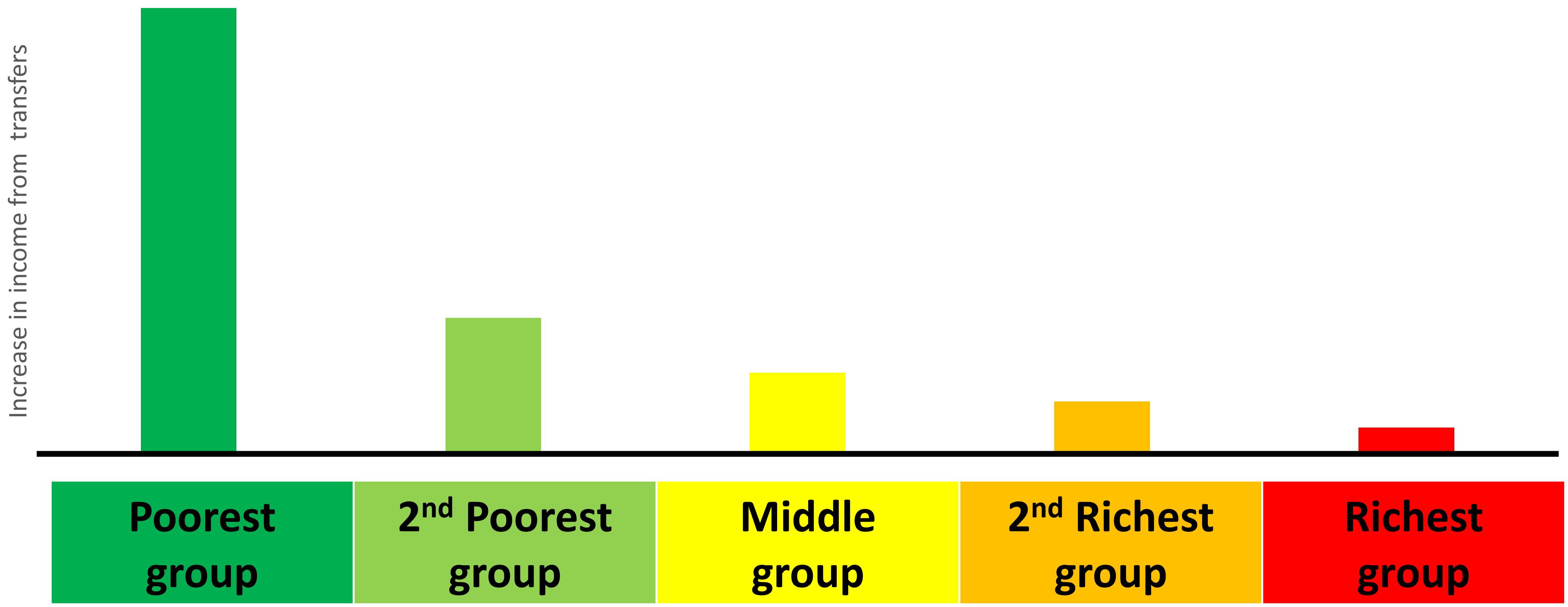


*This information recently became publicly available online through a collaboration between universities, civil society and international organisations.

**Taxes and transfers treatment - Mexico**

*Recent research* in Mexico shows:* Richer households pay more in taxes than they receive in government transfers, whereas Poorer households receive more in government transfers than they pay in taxes

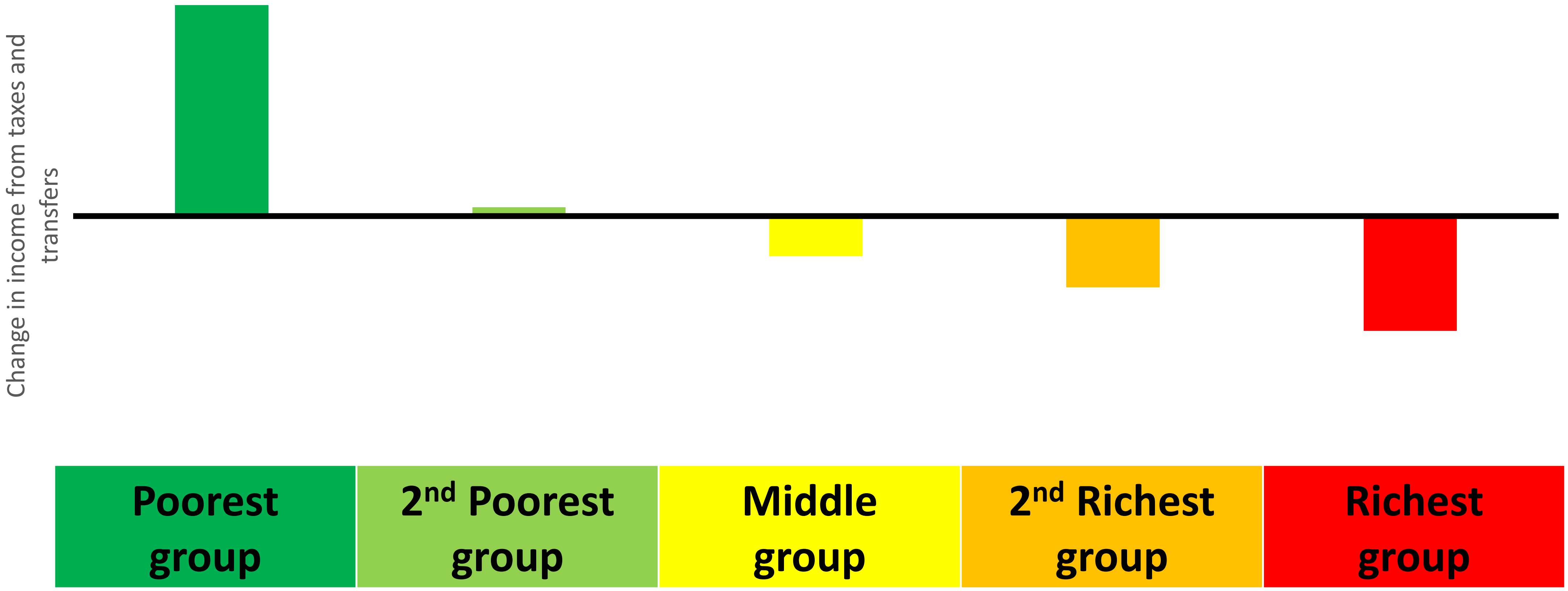


*This information recently became publicly available online through a collaboration between universities, civil society and international organisations.

# *Recent research* in Sri Lanka shows:* Richer households pay a similar share of their income in taxes as Poorer households

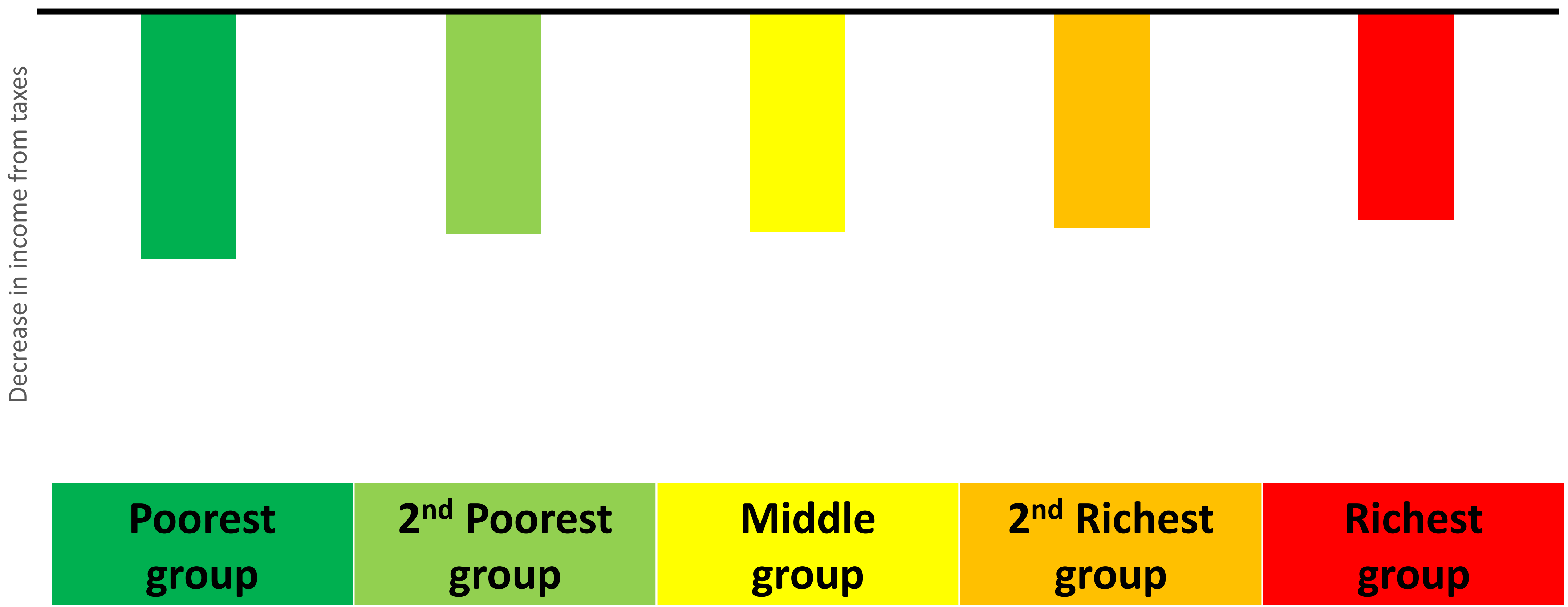


*This information recently became publicly available online through a collaboration between universities, civil society and international organisations.

# *Recent research* in Sri Lanka shows:* Poorer households receive a much larger share of their income in government transfers than Richer households

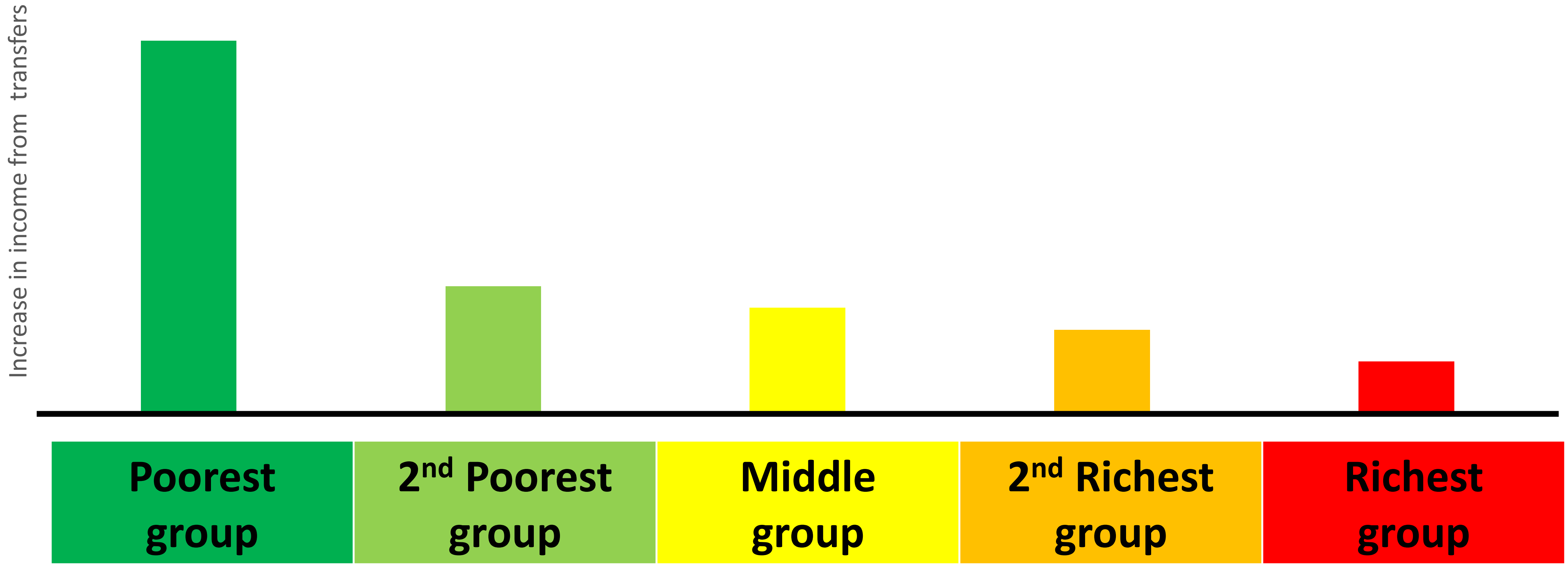


*This information recently became publicly available online through a collaboration between universities, civil society and international organisations.

**Taxes and transfers treatment - Sri Lanka**

*Recent research* in Sri Lanka shows:* Richer households pay more in taxes than they receive in government transfers, whereas Poorer households receive more in government transfers than they pay in taxes

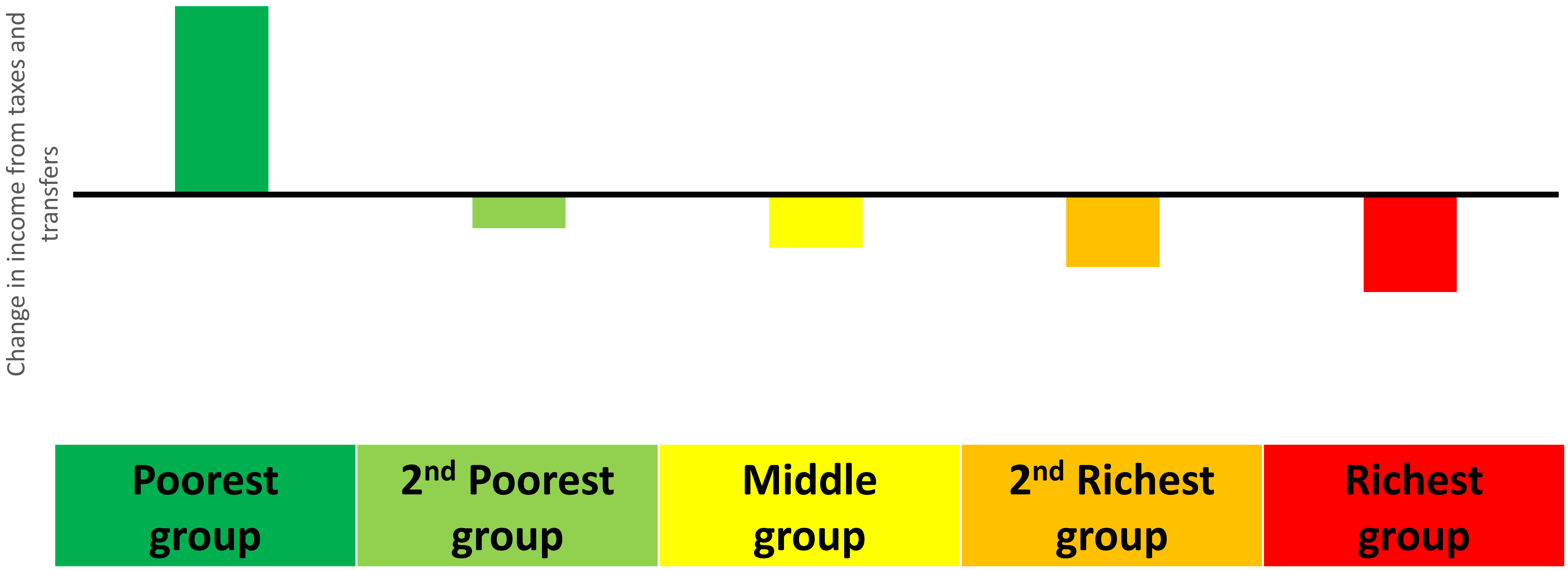


*This information recently became publicly available online through a collaboration between universities, civil society and international organisations.

Taxes treatment - Tanzania

# *Recent research* in Tanzania shows:* Richer households pay a much larger share of their income in taxes than Poorer households

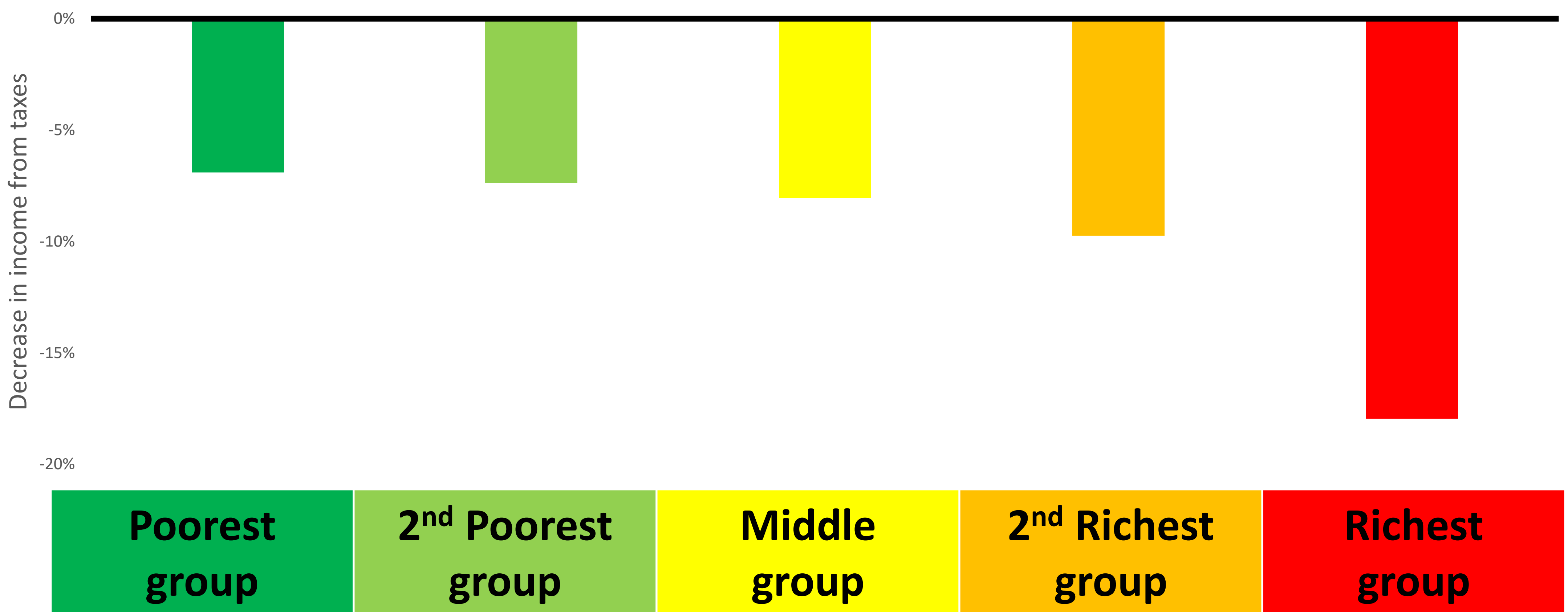


*This information recently became publicly available online through a collaboration between universities, civil society and international organisations.

**Transfers treatment - Tanzania**

## *Recent research* in Tanzania shows:* Poorer households receive a similar share of their income in government transfers as Richer households

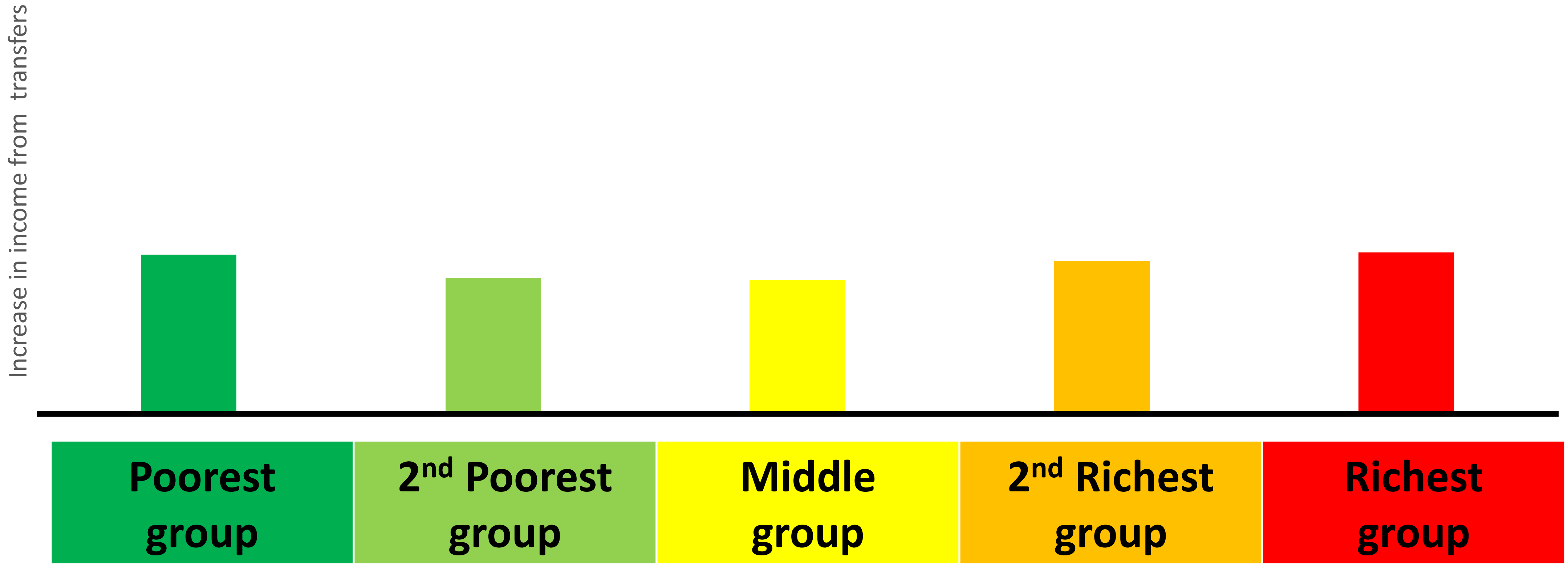


*This information recently became publicly available online through a collaboration between universities, civil society and international organisations.

**Taxes and transfers treatment - Tanzania**

# *Recent research* in Tanzania shows:* Most households pay more in taxes than they receive in government transfers and Richer households pay more than Poorer households

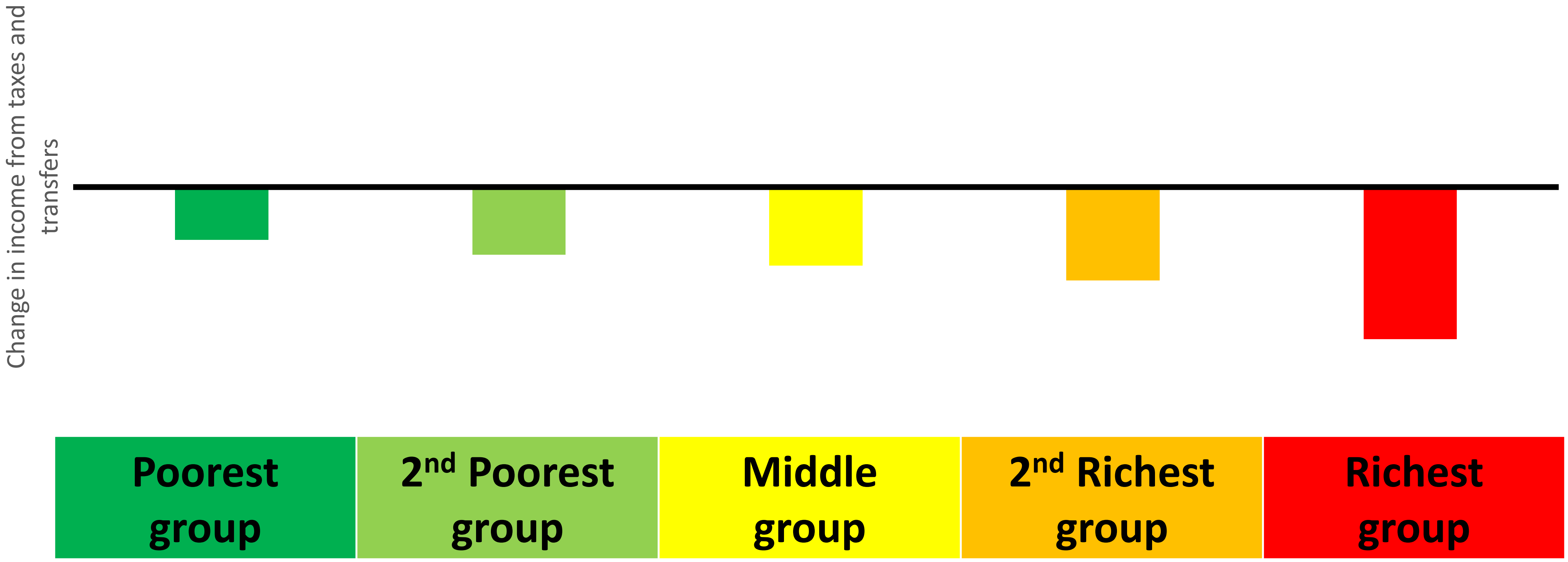


*This information recently became publicly available online through a collaboration between universities, civil society and international organisations.

**Taxes treatment – South Africa**

# *Recent research* in South Africa shows:* Poorer households pay a much larger share of their income in taxes than Richer households

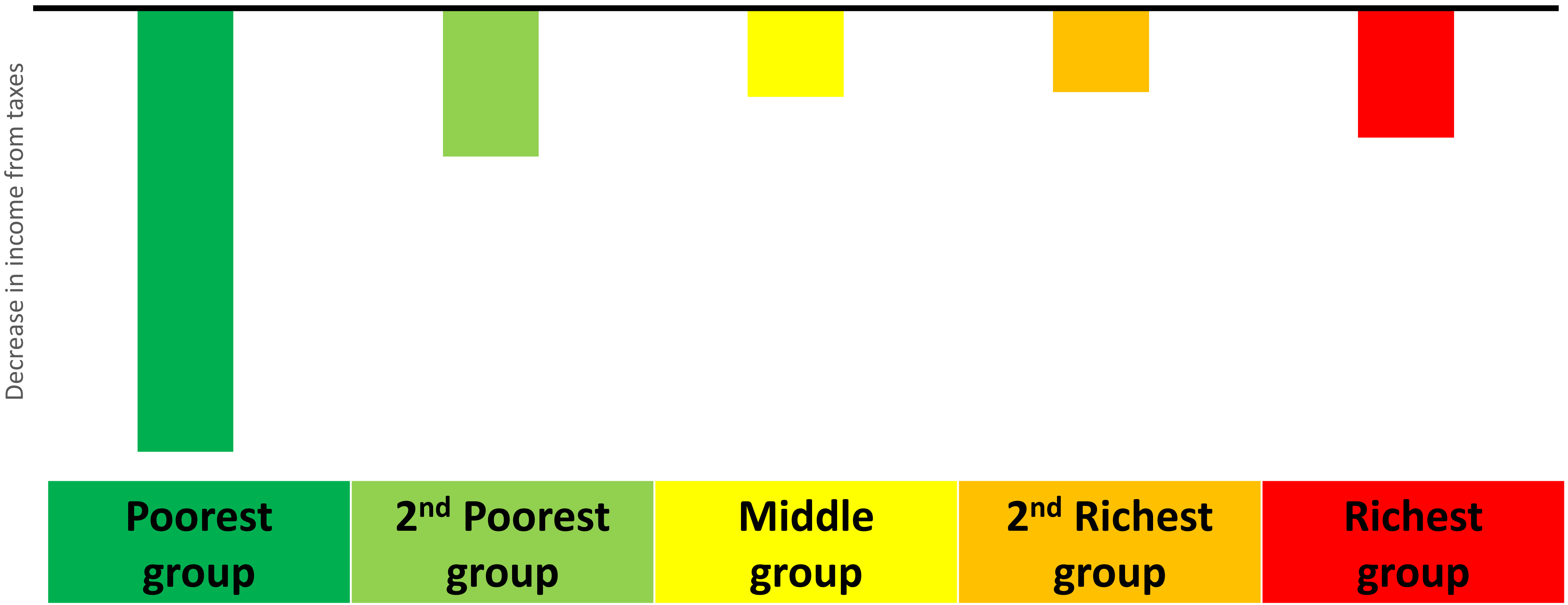


*This information recently became publicly available online through a collaboration between universities, civil society and international organisations.

**Transfers treatment - South Africa**

*Recent research* in South Africa shows:* Poorer households receive a much larger share of their income in government transfers than Richer households

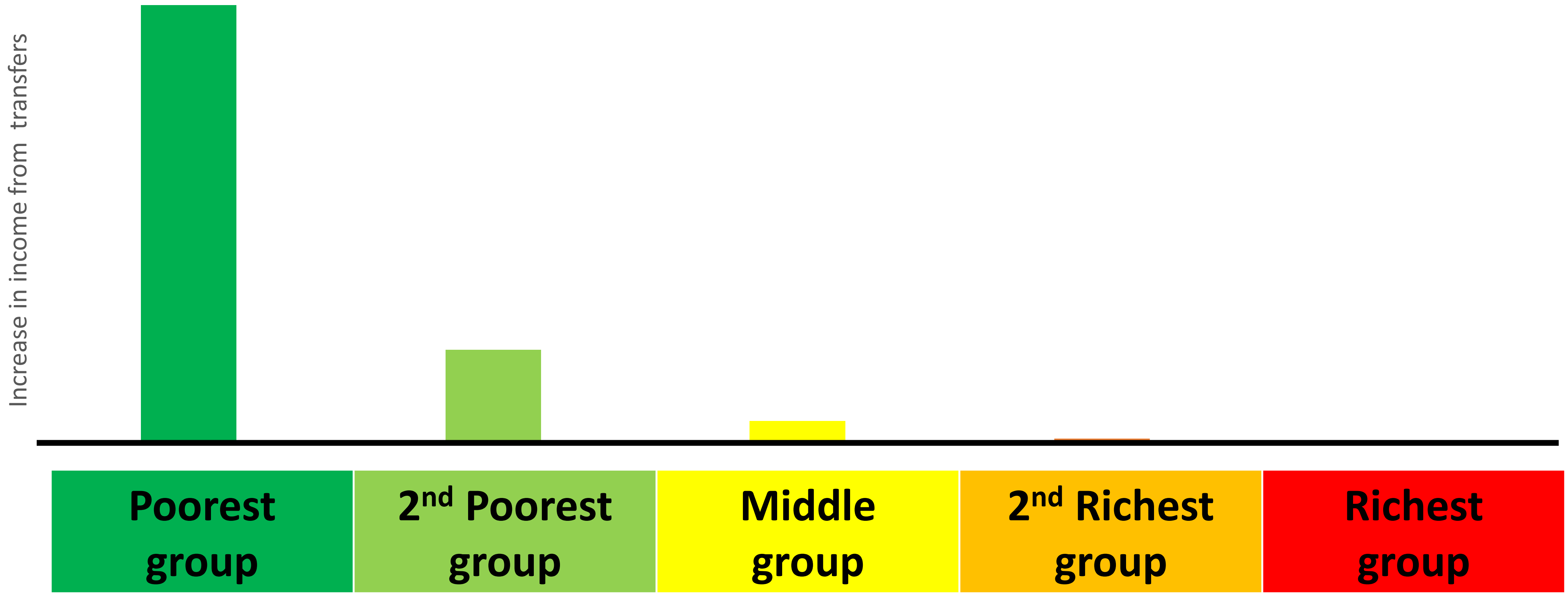


*This information recently became publicly available online through a collaboration between universities, civil society and international organisations.

**Taxes and transfers treatment - South Africa**

*Recent research* in South Africa shows:* Richer households pay more in taxes than they receive in government transfers, whereas Poorer households receive more in government transfers than they pay in taxes

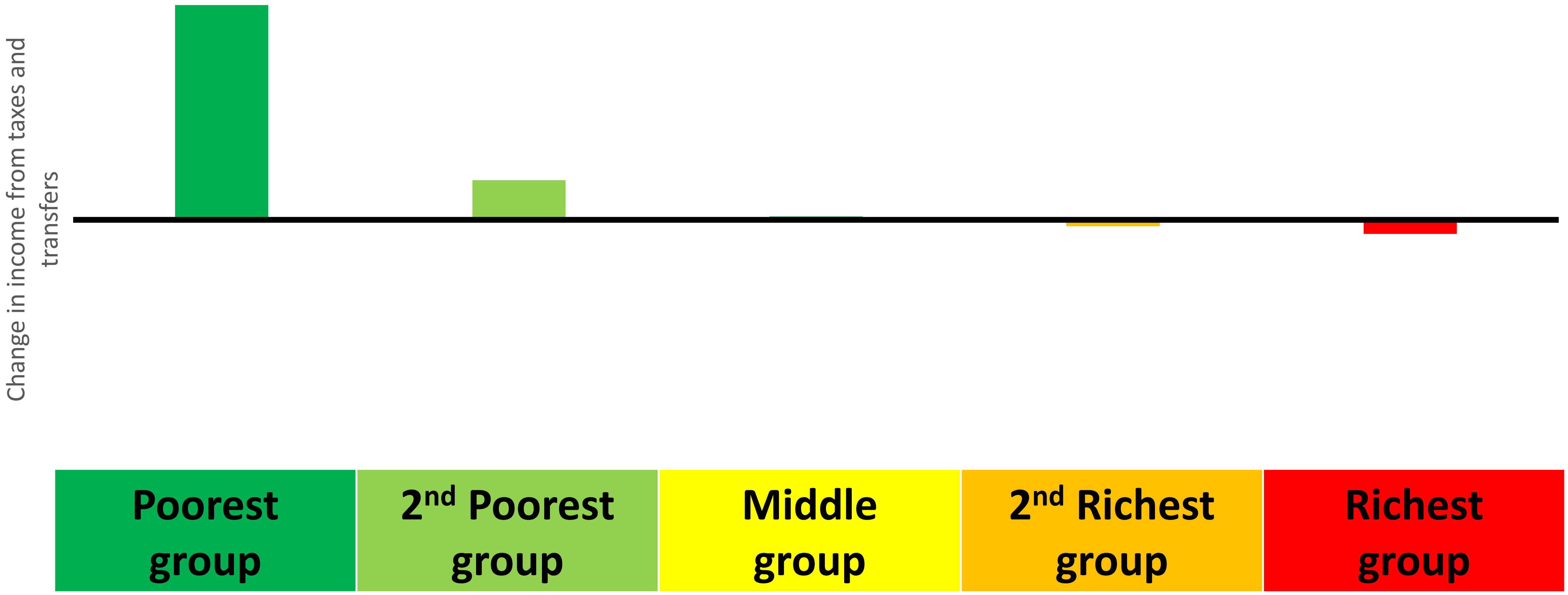


*This information recently became publicly available online through a collaboration between universities, civil society and international organisations.
